# Changing Rainfall Drives Locally Asynchronous Reproduction of Tropical Birds via Modular Trophic Pathways

**DOI:** 10.1111/gcb.70790

**Published:** 2026-03-26

**Authors:** Felicity L. Newell, Ian J. Ausprey, Scott K. Robinson

**Affiliations:** ^1^ Florida Museum of Natural History & Department of Biology University of Florida Gainesville Florida USA; ^2^ Division of Conservation Biology, Institute of Ecology & Evolution University of Bern Bern Switzerland; ^3^ Department of Ecology & Conservation Biology Texas A&M University College Station Texas USA

**Keywords:** Andes mountains, breeding ecology, changing rainfall, ecological threshold, food webs, insectivorous birds, Tropical montane cloud forest, tropical phenology

## Abstract

Phenological shifts are a pervasive response to climate change but remain poorly understood in the hyperdiverse tropics. Combining comprehensive multitrophic datasets and in situ meteorological data, we test classic hypotheses linking reproduction to the timing and magnitude of rainfall across trophic levels in tropical birds. In low‐latitude mountains, breeding was primarily seasonal and varied based on diet. Consistent with the regional timing of wet and dry seasons, bird species that consume primarily nectar or fruit timed breeding to dry season flowering or wet season fruiting with limited variation across elevation and rainfall gradients. In contrast, species that consume arthropods shifted breeding locally, five months in less than a hundred kilometers, as the intensity of the dry season increased. Spatially asynchronous reproduction was repeated in more than 30 insectivore species as the main nesting season switched from before to after the dry season at a threshold in dry‐season insects. Reversed seasonality magnified the short‐term effects of drought as insectivore communities that nested after the dry season reduced reproductive effort—skipping breeding during resource‐limited dry years—whereas communities that nested before the dry season adapted by breeding up to one month earlier. Strong spatial to temporal variation at a ratio of 5:1 suggests limited short‐term behavioral flexibility within restricted breeding seasons timed based on the long‐term magnitude of seasonal rainfall. At higher trophic levels, similar within‐group but different between‐group responses to rainfall magnitude demonstrate quasi‐independent trophic pathways for how tropical food webs link to rainfall. Cumulatively, these results support an ecological tipping point tied to dry season intensity in which rainfall‐mediated ecological constraints compartmentalized functional groups into vertical trophic modules, which responded differently to changing rainfall. Compared with the seasonal stability of nectar‐fruit consumers, the rapid response of insectivores provides an early warning for changing rainfall.

## Graphical abstract

In low‐latitude mountains, we found insectivorous bird communities switched from breeding before to after the dry season at a threshold in dry‐season insects. Localized breeding season reversal of insectivores across wet‐to‐dry gradients magnified short‐term effects of drought and contrasted with the seasonal stability of nectar‐fruit consumers. These results demonstrate modular trophic pathways linked to rainfall magnitude versus timing as rapid response of insectivores provided an early warning for changing rainfall.

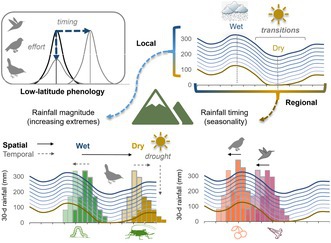

## 1 Introduction

Changing rainfall regimes remain one of the biggest unknowns to human‐induced climate change. Warming temperatures are leading to intensification of the hydrologic cycle as increasing rainfall extremes, including both drought and heavy rain, contribute to ongoing fires and floods. The prevailing hypothesis that the ‘wet get wetter and the dry get drier’ (Held and Soden 2006) can result in increasing seasonal extremes (Newell et al. 2022a). Changes in rainfall magnitude are likely to have profound impacts on tropical ecosystems where biomes are strongly structured by rainfall (Beck et al. 2018). Despite supposedly global syntheses, most tropical systems lack data, especially at low latitudes (Feeley et al. 2016), and the few long‐term studies that exist on birds in undisturbed ecosystems suggest that climate change may be contributing to enigmatic population declines of tropical species (Stouffer et al. 2021; Neate‐Clegg et al. 2021; Blake and Loiselle 2024). Studies also document local adaptation, for example, through changes in morphology (Jirinec et al. 2021; Neate‐Clegg et al. 2024), although the range of ways that species adapt to a changing climate, such as through changes in phenology, remains poorly understood.

Changes in phenology are one of the most pervasive effects of human‐induced climate change (Scheffers et al. 2016). Phenology, or the timing of biological events in relation to weather (short‐term variability) and climate (long‐term means), links vertebrate populations to climate‐mediated resource availability. Reproductive timing is especially important for income‐breeding birds that depend on concurrent food resources to provision growing chicks (Drent and Daan 1980; Williams et al. 2017). Linking consumer‐resource responses, food has been experimentally shown to initiate reproduction of tropical birds both in the field (Komdeur 1996) and the lab (O'Brien and Hau 2005). However, food‐mediated hormonal pathways leading to reproduction (Williams et al. 2017) have often been considered supplemental, as even 1‐h changes in daylength can initiate reproduction in a rainforest antbird (Thamnophilidae) (Hau et al. 1998). Thus, there is limited understanding of phenology at low latitude where the seasonal variation in day length (photoperiod) and temperature that synchronize ecological systems are relaxed.

In the tropics, rainfall becomes the primary driver of seasonal growth and reproduction (Skutch 1950; Wolda 1988; Van Schaik et al. 1993), mediating the availability of food resources across trophic levels. In most of the tropics, rainfall varies throughout the year with wet and dry seasons related to the north–south movement of the Intertropical Convergence Zone (ITCZ), a band of clouds and rain that tracks the thermal equator. In seasonal dry forest and savanna, birds initiate breeding as biotic resources increase with returning rain (Poulin et al. 1992; Hidalgo Aranzamendi et al. 2019), leading to models that predict reproduction coincides with the onset of rain after the dry season (Quintero et al. 2014). However, this model is inconsistent with records of extended or even year‐round breeding of birds in equatorial rainforest (Wyndham 1986; Johnson et al. 2012; Stouffer et al. 2013), where reproductive activity may increase with sunshine (Berman et al. 2023). To examine phenology, studies often pool data regionally (Padmanabhan and Yom‐Tov 2000; Duursma et al. 2019; Moreno‐Palacios et al. 2025), which may be problematic if breeding and/or climate vary locally. The role of rainfall in regulating phenology across complex food webs from plants to insects to vertebrates is a frontier in understanding the long‐term consequences of climate change on tropical ecosystems.

**FIGURE 1:**
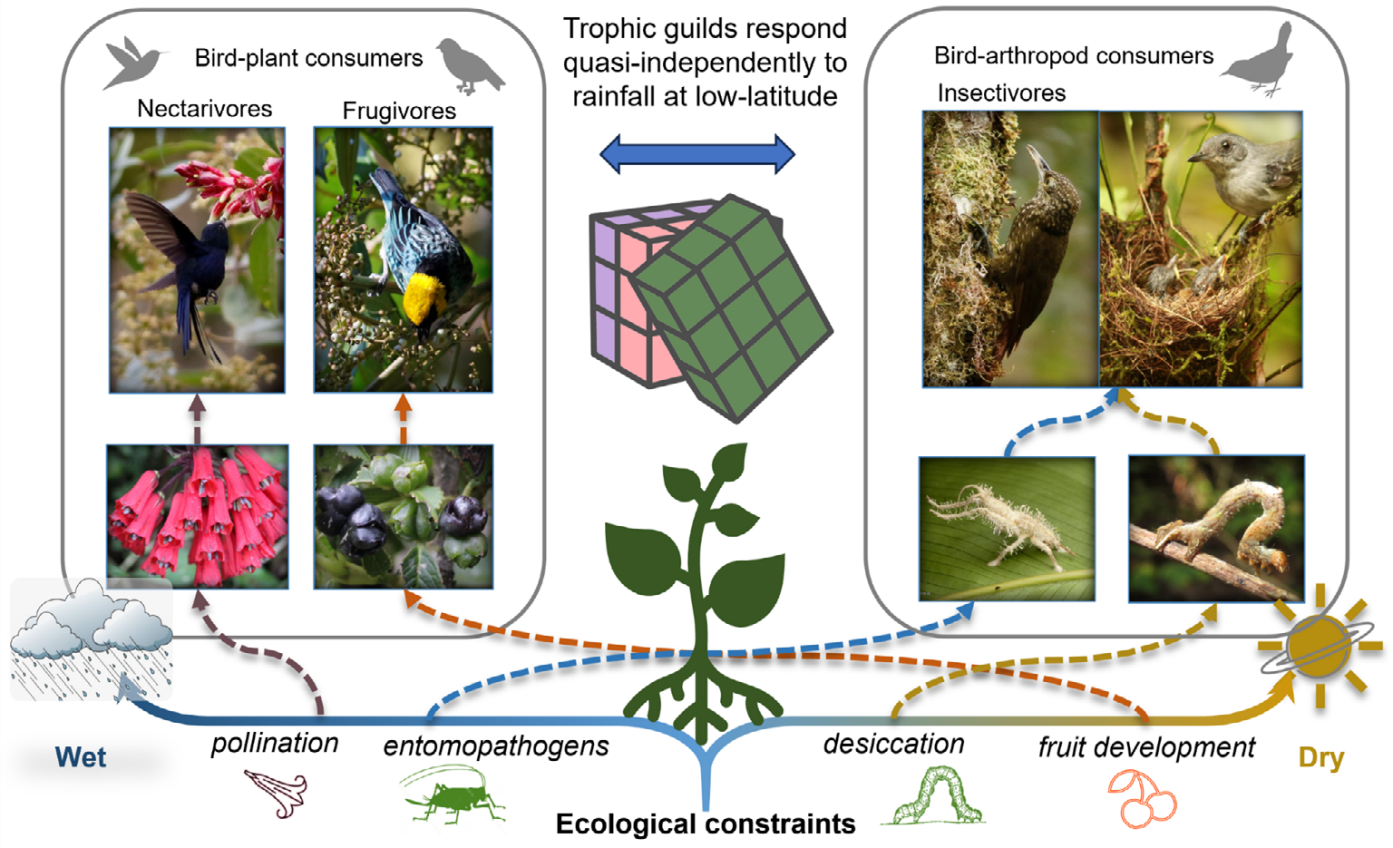
We hypothesize that the phenology of higher trophic levels (birds) responds quasi‐independently to changing rainfall based on different ecological constraints that regulate their primary resources: flowers, fruit, and arthropods (insects plus spiders). Different rainfall‐mediated constraints are predicted to result in “block” or compartmentalized responses within food webs as ecological communities adapt phenology to changing rainfall at low latitudes. Modular trophic pathways (arrows) connect consumers to ecological constraints along a gradient of spatiotemporal variation in rainfall as both wet and dry extremes are predicted to be limiting. Photo credits: Felicity Newell, Ian Ausprey, Florida Museum of Natural History (FLMNH), Andreas Kay.

**FIGURE 2:**
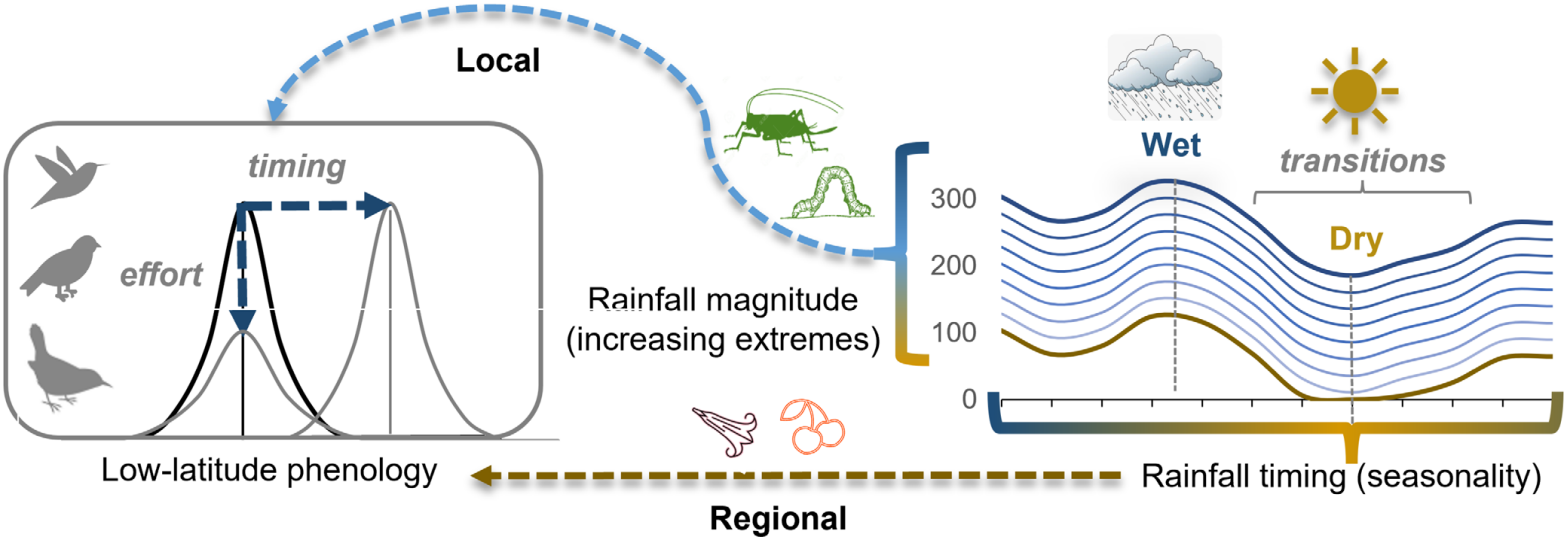
We disentangle how the phenology of different trophic guilds relates to short and long‐term variability in the timing and magnitude of seasonal rainfall via effects of rainfall accumulation during wet, dry, and transition seasons on resource availability (flowers, fruit, arthropods). Sampling local variation in climate at a small spatial scale in low latitude mountains, our study design controlled for rainfall seasonality, which varied spatially by < 2 weeks. For reproductive timing, we used peak dates because of low levels of breeding year‐round (~1%). To measure effort, we quantified the maximum percentage of reproductive events within the population. Dashed phenology arrows indicate timing shifts and/or effort decreases.

The complex topography of tropical montane systems provides a unique opportunity to examine the effects of climate at small spatial scales with limited biogeographic change in species composition. Here, for the first time, we examine how food web phenology varies over space and time across low‐latitude elevation and rainfall gradients. Hypotheses for bird nesting in the tropics developed out of classic natural history observations by Skutch in Costa Rica and Moreau in Africa (Moreau 1950; Skutch 1950), including often unrecognized fieldwork by natives such as Salimu Asmani. Skutch proposed that species nest during rainfall‐mediated floral, fruit, or insect maxima depending on their diet, while Moreau observed that although latitude was important across the African continent, timing relative to rainfall varied locally with no general driver. Expanding on the concept of trophic guilds, which classifies animal consumers based on similar resource use often involving competition (Blondel 2003), we directly quantify the availability of seasonal resources at basal trophic levels. This invokes network approaches that identify vertical subunits or modules within food webs based on strong within‐group but weak between‐group interactions (May 2001; Grilli et al. 2016). In complex food webs, these interconnected, quasi‐independent consumer‐resource blocks are predicted to differ in their response to changing rainfall based on different ecological constraints that regulate the abundance and biomass of often taxonomically unrelated groups (Figure 1). Thus, as ecosystems transition along climatic gradients at low latitudes, we predict that modular trophic pathways compartmentalize and drive differences in phenology at higher trophic levels (Figure 2).

Based on the modular breeding hypothesis, we made several predictions. Even at low latitudes, income‐breeding birds are expected to reproduce seasonally if resources are regularly limiting during part of the year (Martin 1987). Thus, we expected reproductive timing to relate to 30‐year means (long‐term climate), whereas we expected reduced reproductive effort during years when resources are scarce (interannual variability) (Grant et al. 2000). Recent synthesis of avian reproductive data across Colombia supports the idea that tropical breeding seasons are primarily seasonal, especially at higher elevation (Moreno‐Palacios et al. 2025). For bird‐plant consumers, main floral and fruiting seasons are predicted to be mediated by opposing constraints in which flowers require dry conditions for pollination (Lawson and Rands 2019), whereas fleshy fruits require moisture for development (Mendoza et al. 2017). Thus, we expected nectarivores and frugivores to reproduce in opposite seasons, concurrent with dry season peaks in bird‐pollinated flowers and wet season peaks in bird‐dispersed fleshy fruit. Additionally, we examined evidence for staggered breeding which has been proposed to reduce resource competition in hummingbirds (Stiles 1985) although also potentially related to morphological specialization (Tinoco et al. 2017). For bird‐insect consumers (2/3 of tropical bird communities), insects may be sensitive to both desiccation and entomopathogens (Newell et al. 2023a). From available arid system models, we expected insectivorous species to nest at the beginning of the rainy season as insects increase with returning rain. Omnivory can be complex as many species depend on insects for feeding young (e.g., diet switching). Here, we expected a few omnivorous species to be generally similar to insectivores, while granivorous species were poorly represented in tropical montane rainforest.

We focused our analyses on food web hypotheses that link rainfall‐mediated resources to ecosystem function at local to regional scales. We examined and controled for alternative hypotheses primarily related to latitude (Baker 1939; Moreau 1950) by working at small spatial scales. In mountain systems, we also examined the hypothesis that timing varies with elevation, because at higher latitudes breeding seasons are delayed and shorter at higher elevation (Boyle et al. 2016). However, in low‐latitude evergreen forest, we did not expect birds to nest later at higher elevations because of limited seasonal variation in temperature. Here, we do not address leaf phenology because our previous research on arthropod biomass (insects plus spiders) quantitatively showed that weak plant‐level effects of leaf flush do not extend to landscape scales because of limited seasonal variation (Newell et al. 2023a), contrary to previous hypotheses for equatorial rainforest (Fogden 1972).

## 2 Methods

### 2.1 Montane Climate in the Andes of Northern Perú

**FIGURE 3:**
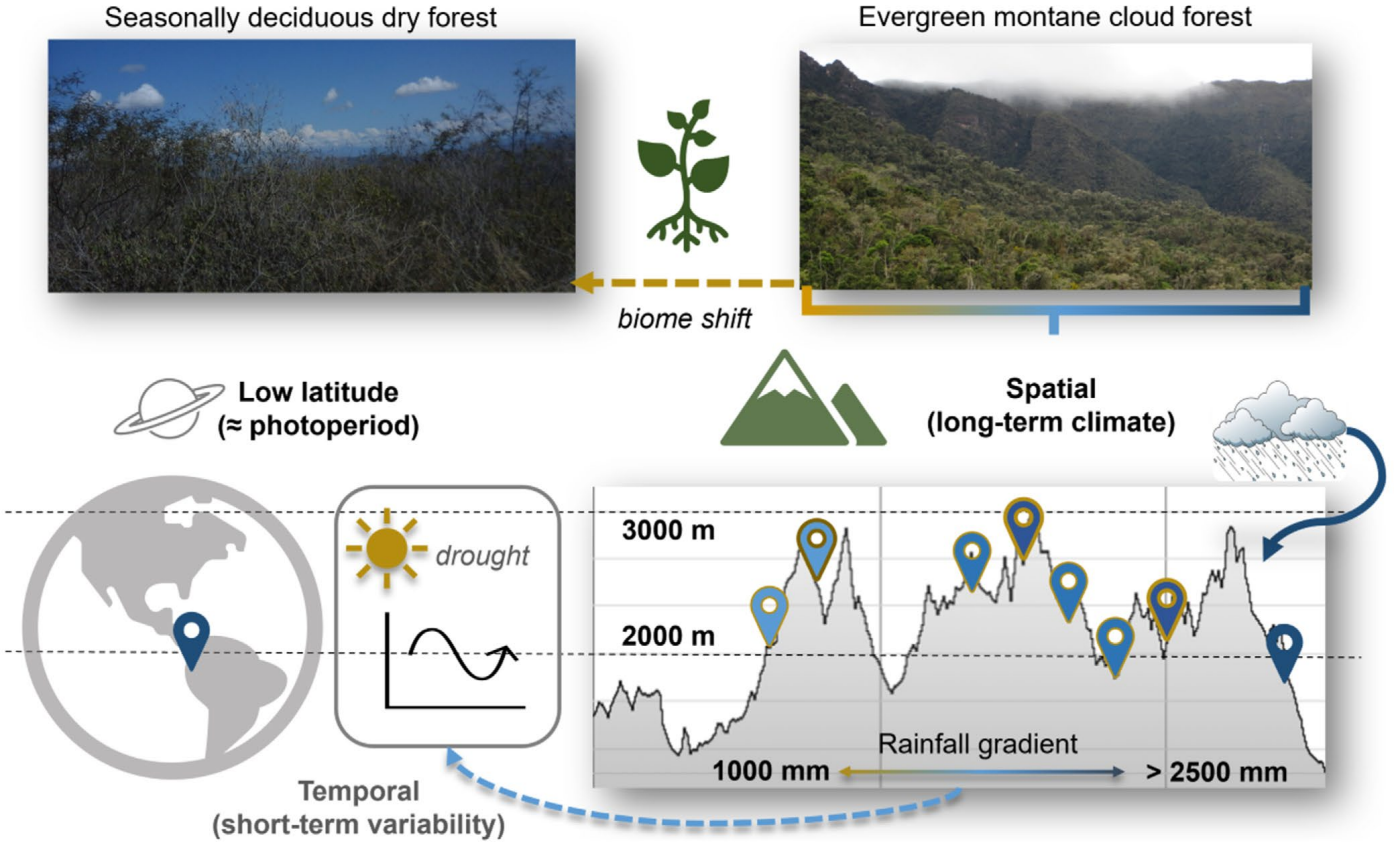
To test the modular breeding hypothesis, we used a network of sites in tropical montane cloud forest as a natural experiment. We examined spatiotemporal variation in phenology of diverse communities (8 landscapes over 5 years) across elevation and rainfall gradients at 5°–6° S in the Andes of northern Perú (Yungas). Collecting concurrent multitrophic datasets along with in situ meteorological data, we examined short‐ and long term effects of changing climate across a 10,000 km^2^ area in which mean annual rainfall (1000–2500 mm) was uncorrelated with elevation (1700–3100 m). Cloud forest sites spanned the eastern slopes of the Andes to interandean valleys before evergreen forests transition to seasonally deciduous dry forest along the Utcubamba and Marañon rivers. Photo credits: Felicity Newell, FLMNH.

Here, we use spatiotemporal variation in montane rainfall across a 10,000 km^2^ area along the eastern slope of the Andes as a natural experiment to examine how local changes in rainfall magnitude influence phenology while controlling for variation in timing of wet/dry seasons, which varied spatially < 2‐weeks across the region (Figure 3, Figure S2a). Located between 5°–6° S and 77°–78° W in the Andes of northern Peru (Amazonas/San Martin), our cloud forest study system (8 landscapes over 5 years) spanned a 1700–3100 m elevation and 1000–2500 mm rainfall gradient (Figure 3, Figure S1, Table S1). In this region, March/April are the rainiest and July/Aug the driest months of the year (Figure S1b). Daylength varied seasonally by 41–46 min with < 5‐min differences among landscapes while temperature varied seasonally by < 2°C. Key to separating climatic drivers, mean annual temperature and precipitation were not correlated across four watersheds (Newell et al. 2022a).

In situ weather data were collected for each landscape beginning in 2015–2016. This analysis used 5 years of data, including a strong drought in 2016 related to the El Niño‐Southern Oscillation (ENSO) (Newell et al. 2022a). We calculated short‐ and long‐term climate for each landscape based on the normal‐ratio method (Newell et al. 2022a). Local data were scaled to interannual variability over 50 years from regional weather stations by applying a mean ratio between stations to fill missing data; daily rainfall values for each landscape were then averaged across stations. We averaged the timing and magnitude of seasonal rainfall across years to obtain long‐term climate for each landscape (rainfall normals). For the spatial analysis, we examined variation in mean 90‐day rainfall accumulation by month and then focused model selection on seasonal rainfall periods with the best fit (Figure S2a). For temporal analysis, we examined previous rainfall accumulation at different time lags. Prior to analysis, we examined correlations among climatic variables. We present key variables here, although a broad range of time lags and metrics (temperature, humidity, rainfall, cloud cover) were examined during initial exploratory analyses. See published paper for additional details on local and regional climate (Newell et al. 2022a).

### 2.2 Seasonal Sampling of Spatiotemporal Variation

Along with in situ meteorological data, we collected comprehensive multitrophic datasets on phenology of montane birds, as well as the seasonal abundance of flowers, fruit, and arthropods (Figure S3). To quantify spatiotemporal variation, landscape‐scale fieldwork was conducted at different times of year during six 3–6‐month sampling “snapshots” over 5 years, 2015–2019 (Figure S2b). During each “snapshot,” landscapes were visited consecutively every 1–2 months for year‐round coverage across all months except March/April when landslides and road closures limited our ability to travel safely. Sampling was also reduced at the lowest elevation site (Venceremos) when the park guard station was closed due to safety concerns from neighboring communities. A “snapshot” approach allowed us to examine seasonal, interannual, and spatial variability at as broad a spatiotemporal scale as possible, given limited time and funding constraints. We collected data during dry and transition dry‐to‐wet seasons from Jun/Jul–Nov for 3 years, 2015–2017. Additional transition wet‐to‐dry season data were collected from May–Jul for 2 years, 2018–2019, and we collected data for one wet season from Dec–Feb 2018/19. Within each landscape 3–9 sub‐sites were located < 5 km apart within a 300‐m elevation band as part of a broader study examining the interaction of climate and land use on Andean bird communities (Ausprey et al. 2022, 2023; Mamani‐Cabana et al. 2023). Overall, we collected data during 129 visits to eight landscapes over 5 years, including data on phenology and resources from 60 subsites (see Supporting Information: Methods).

### 2.3 Abundance of Flowers and Fruit

We quantified the seasonal abundance of flowers and fruit, surveying point‐transects on each visit from 2016 to 2019; results are presented elsewhere for phenology of new leaves (Newell et al. 2023a). Points were located at 30‐m intervals along transects, and we surveyed a fixed area and/or a fixed number of plants (see Supporting Information: Methods). At each point, we recorded the number of flowers (buds, open, dead) or fruit (unripe, ripe, rotten) per plant by phenological phase. For high numbers, we counted aggregate units and then multiplied by the average number of flowers per inflorescence or fruits per infructescence. On each visit, we sampled an approximate area of 0.5 ha (12.5‐m fixed‐radius × 10 points) consistent with previous line‐transect approaches (Weinstein and Graham 2017); all transects were surveyed by the same observer (F.L.N.).

To examine resource availability for nectarivores and frugivores, we focused on bird‐pollinated and bird‐dispersed plants, which we documented and identified using photographs (see Supporting Information: Methods for classification using a syndrome‐based approach). As part of our surveys, we took > 10,000 photos of plants for identification purposes. Across elevations in a plant biodiversity “hotspot” with > 2000 species (Tropicos 2021), we identified 238 species of plants in 89 genera from 56 families likely used by birds (Table S8). Common plant genera at one or more landscapes included bird‐pollinated: *Aphelandra* (Acanthaceae), *Brachyotum* (Melastomataceae), *Centropogon* (Campanulaceae), and *Tillandsia* (Bromeliaceae); bird‐dispersed: *Miconia* (Melastomataceae), *Psychotria* (Rubiaceae), *Solanum* (Solanaceae), and *Viburnum* (Adoxaceae); or both: *Palicourea* (Rubiaceae), *Psammisia* (Ericaceae), *Fuchsia* (Onagraceae), and *Tristerix* (Loranthaceae).

To model the seasonal abundance of flowers and fruit (Figures S9 and S10), counts were summed by visit and landscape. We used polynomial regression with a Gamma distribution and log‐link function, which provided the best fit to the data; points without detections of flowers or fruit were assumed to represent low counts somewhere on the landscape, and zeros were replaced with 10, the smallest number that converged. Each visit was considered to be a replicate, and we used quadratic mixed models with the number of points as an offset; we included a random intercept by sampling method to incorporate less intensive sampling in 2016 (see Supporting Information: Methods), as well as a repeated measure by sampling period. For regional analysis, the landscape was included as a random effect. We present abundance of flowers and fruit per 0.5 ha (10 points) back‐transformed from the log scale.

### 2.4 Foliage Arthropod Biomass

We quantified seasonal changes in arthropod biomass according to our published arthropod biomass model (Newell et al. 2023a). We sampled foliage arthropods using modified branch beating to rapidly capture a broad spectrum of taxa dwelling on leaves (Cooper and Whitmore 1990; Basset et al. 1997; Leather 2005). Sampling was conducted from 2015 to 2019, and on each visit, 20 points were sampled at 30‐m intervals along transects, half by the same observer (F.L.N.) and half by two trained observers that varied among years. At each point, we vigorously beat the nearest shrub several times over a sweep net, sampling a 0.5‐m^2^ area with > 50% foliage (calibrated to sweep net poles). Using a sweep net instead of an open cloth to catch falling insects greatly reduced the escape of active taxa that had difficulty propelling themselves over the side of the net, while we were also able to close the net upon completion while counting. This approach avoided the typical problem that branch beating samples mobile species poorly (Cooper and Whitmore 1990) while also rapidly capturing 50%–70% of Lepidoptera larvae compared to visual counts (Bodner 2011).

We modeled changes in arthropod biomass in response to spatiotemporal variation in rainfall across landscapes and years (Newell et al. 2023a). In evergreen moist forest, biomass responded to increasing and decreasing rainfall on different time scales with an additive effect of dry air, especially during periods of low rainfall. Rainfall‐driven biomass models with a Gamma distribution and log‐link function included (i) a curvilinear response to 90‐day rainfall during the rainy to dry season and (ii) linear increases with 30‐day rainfall < 133 mm at the beginning of the rainy season. This field‐based model was used to output arthropod biomass by month from 1970 to 2019 (Newell et al. 2023a) based on long‐term regional weather data scaled to each cloud forest landscape by the normal ratio method (Newell et al. 2022a). We then used monthly biomass as the input to model long‐term seasonal means (biomass normals). Seasonal models on a daily time step were run using generalized additive mixed models (GAMMs) with a cyclic cubic regression spline and initial knots set at 12 months, a temporal smooth by date, plus a random intercept by year for each landscape (Newell et al. 2023a).

To examine any effects of rain on foraging activity, we ran models for both “available” and “effective” biomass (Figure S11), multiplying monthly estimates by the proportion of daylight foraging hours per landscape assuming rain limits foraging activity (Öberg et al. 2015). To calculate reductions in foraging time based on rain, we averaged hours of daytime rain per month from local rain gauges at each landscape, then divided by total daylight hours based on sunrise and sunset times each month. We included light rain < 0.1 mm when bird activity appeared to be reduced on days with constant cold wet drizzle. Light rain reduced potential foraging time by 4%–28% per wet season month, whereas moderate to heavy rain reduced foraging time < 7% except at Venceremos where heavy rain was common.

### 2.5 Phenology of Cloud Forest Bird Communities

From 2015 to 2019, we captured birds using 2 days of mist netting on each site‐visit to record evidence of reproduction, juveniles, and molt throughout the year. Birds were banded with uniquely numbered aluminum bands, including hummingbirds, as well as color bands. For each individual, we took morphological measurements including wing and tail to the nearest 1 mm; tarsus and bill to the nearest 0.1 mm; and mass to the nearest 0.1 g. We recorded breeding characteristics (BP = brood patch, CP = cloacal protuberance), juvenal plumage, gape, and eye color. We scored molt limits and flight‐feather molt using molt cards (Ginn and Melville 1983). We took a headshot and spread‐wing photo of all individuals and documented any breeding or juvenile characteristics with photographs; this allowed us to review and confirm activity and backdate reproductive and related events (e.g., juvenile age). We developed species‐specific criteria to age birds (Chumpitaz et al. 2018) based on standard ageing characteristics including molt limits, skull ossification, and bill striations or corrugations (Mulvihill 1993; Pyle 1997). We used Wolfe–Ryder–Pyle (WRP) molt‐cycle ageing codes developed for tropical systems (Wolfe et al. 2010; Johnson et al. 2011) as well as available tropical guides (Guallar and Galles 2009; Johnson and Wolfe 2017).

In hyperdiverse tropical communities where species often occur at low abundance, we developed an integrated approach to examine avian phenology using sparse data (see Supporting Information: Methods). To maximize the utility of intensive and systematically sampled reproductive and related events documented by photographs and molt cards, we combined different phases of the annual cycle (phenophases) for current plus backdated reproductive events into a single model controlling for sampling effort. This avoided the common approach of a priori pooling across taxa with small sample sizes (Poulin et al. 1992; Berman et al. 2023) and allowed us to quantitatively combine multiple sources of data to independently model annual cycles for different populations of the same species across climatic gradients at the community level (Figures S12–S15). In the combined‐breeding model, we viewed each visit to a landscape as a sampling window representing reproductive activity in current and previous months. Reproduction is followed by an increase in juveniles (Poulin et al. 1992), while molt typically follows breeding in tropical rainforest (Johnson et al. 2012). Although molting strategies can vary among species (Jenni 2020), in our system, molt primarily followed breeding (Figure S4c), with the exception of young birds prior to their first breeding season, which we excluded from our analysis (see Supporting Information: Methods). During molt initiation (≤ 30 days), 26% of molting individuals retained breeding characteristics (BP or CP), while during advanced stages of molt, only 2% of individuals overlapped breeding and molt in the majority of the cloud forest bird community. This combined approach might be less effective in drier systems if molting cycles and the relationship between breeding and molt are more variable (Schondube et al. 2003; Nwaogu et al. 2019).

We classified species into trophic guilds (Tables S9 and S10) based on ≥ 50% of a single resource type using currently available dietary percentages from the Elton trait database 1.0 (Wilman et al. 2014). For species with 50–50 splits, we used nectar or fruit to classify by trophic guild, as most species also consume insects. According to broad diet categories from the database, many of the frugivores in our system consume 50% insects, but we observed substantial fruit in fecal samples and classified these species as frugivores. One exception was *Turdus fuscater* , which we classified as an omnivore because we detected minimal evidence of fruit in fecal samples. We defined omnivores as species that consume < 50% of a single resource type. Although common in agriculture, omnivorous diets were uncommon in cloud forest (Ausprey et al. 2022), and inside the forest, we captured few omnivores, primarily Passerellidae (New World sparrows, brushfinches, bush tanagers). We captured only two granivores (> 50% seeds), which were grouped with omnivores (Passerellidae: *Zonotrichia capensis* , Columbidae: *Zentrygon frenata* ). In the final analysis, omnivores were pooled with insectivores because of generally similar timing.

### 2.6 Statistical Analysis

#### 2.6.1 Model Selection of Functional Traits, Climate, and Resources

All statistical analyses were conducted using Program R v4.3.1 (R Core Team 2024), and we ran models using the R package gamm4 v0.2.6 (Wood and Scheipl 2020). To examine spatiotemporal variation in phenology across communities, we ran separate models for each species (spp.) by landscape, which we refer to as a population (pop.) (see Supporting Information: Methods). This approach combined active plus backdated phenophase observations to run cyclical generalized additive models for monthly counts of reproductive events weighted by sampling effort. From each model, we extracted peak timing (Figure 2b, Figure S4), occurrence of any secondary peaks in a different season (bimodal), as well as reproductive effort, which we quantified as the maximum percentage of reproductive events within the population. For spatial analyses, we used population‐level phenology metrics pooled across years as the replicate. For temporal analyses, we ran models by year for 48% of populations over 5 years to examine interannual variation in the timing and magnitude of reproduction for local populations (species‐by‐landscape‐by‐year).

We related population‐level phenology to rainfall and resources using linear and generalized linear mixed models, including spatial and phylogenetic repeated measures. Phenology metrics were analyzed using either a Gaussian or a Gamma distribution based on the best fit to the data; occurrence of bimodal breeding was analyzed using a binomial distribution with a log‐link function; plots were also visually inspected for outliers. To analyze peak timing as a linear model including random effects for repeated measures (landscapes, species), we first scrolled through Julian start dates by month using model selection to find the best fit for each trophic guild. For subsequent analyses, we used start dates of 1 April and 1 October for insectivores and frugivores, respectively, which were consistent with solar equinoxes and regional rainfall cycles. For nectarivores, this was not an issue in this region as breeding did not cross the calendar year.

We used Akaike's Information Criterion for small sample sizes (AIC_C_) to select the most parsimonious model compared to a null model (Tables S11–S16); models with ΔAIC_C_ < 2 were considered equivalent. We examined models for functional traits prior to including climate data by trophic guild. We focused our analysis on diet, which was the only functional trait with strong support (see Newell 2021 for other traits examined). Finally, we ran models to examine interactions between diet and monthly rainfall across the cloud forest bird community. To control for any phylogenetic structure in our analysis, we used a mixed‐model approach because other methods to incorporate correlation structure based on phylogenetic trees do not allow for intraspecific variation, and our dataset included multiple phenology metrics for the same species across different landscapes (local adaptation). For the initial null model, we included a crossed random intercept for repeated measures by species and landscape (1|species) + (1|landscape). To examine how phenology was structured by phylogeny, we compared the null model to nested models including family (1|family/species) and genus (1|family/genus/species). For all functional and climate models, we included the full nested random intercept for phylogeny and landscape (1|family/genus/species) + (1|landscape).

Model fits were examined using adjusted *R* ^2^, and we present parameter estimates with 95% confidence intervals (CI) for top models. For analysis before and after the dry season, we ran models with and without two transitional sites (Berlin, San Lorenzo), which were less seasonal (Figure S6). Group‐level effect sizes (means) were calculated using the R package emmeans v1.8.7 (Lenth 2020), which outputs asymptotic confidence intervals for generalized linear models on the response scale; regression models were visualized using the R package visreg v2.7.0 (Breheny and Burchett 2017). To examine rainfall‐mediated resource thresholds, we used the R package segmented v1.6.4 (Muggeo 2008) to fit breakpoint models for insectivore families.

#### 2.6.2 Abundant Versus Rare Bird Species

Simulations show that retaining species improves the interpretation of trait‐based analyses in hyper‐diverse tropical systems, which are often dominated by a few common and many rare species (Beck et al. 2024). We systematically collected phenological data on the full understory bird community across eight landscapes over 5 years, but input sample sizes varied among models (populations) depending on the number of reproductive or related events observed, the number of individuals captured, and the span of months with captures. To examine species‐level responses and avoid pooling, we analyzed sparse data and ran community analyses with and without less abundant species with small sample sizes. This approach allowed us to compare our results based on the most robust models for abundant species as well as maximize phylogenetic diversity with the inclusion of rare species, which contribute substantially to tropical diversity. To examine the effects of this variation in sampling effort on our results, we output parameter estimates and model fit using a set of four different filters (Table S3), which we developed based on initial exploratory analysis examining how phenology metrics changed with sampling effort.

Overall, the directionality of our results and conclusions was similar for populations with large to small sample sizes, although for more intensive metrics, large sample sizes reduced variability. We primarily present results for Filter1, which included the largest number of species for plant‐based trophic guilds [*N*_F1_ = 86]. In general, frugivores were more variable than other trophic guilds because (i) diets often include a high proportion of insects, and (ii) many species move through the canopy in large flocks and are less likely to be captured in understory mist‐nets. For spatial shifts in insectivore timing, dropping models with reduced sample sizes in our analysis increased the variance explained from 22% to 43% but did not change the results. For bimodal breeding, we present results for Filter2 because the probability of detecting bimodality increased with greater sampling coverage, but the number of species with high sampling effort decreased for both nectarivores and frugivores. For comparison of trade‐offs between sampling coverage and the number of species, parameter estimates, and model fit are output for different sampling filters (Tables S4–S6).

#### 2.6.3 Confirmatory Path Analysis to Examine Trophic Linkages

**FIGURE 4:**
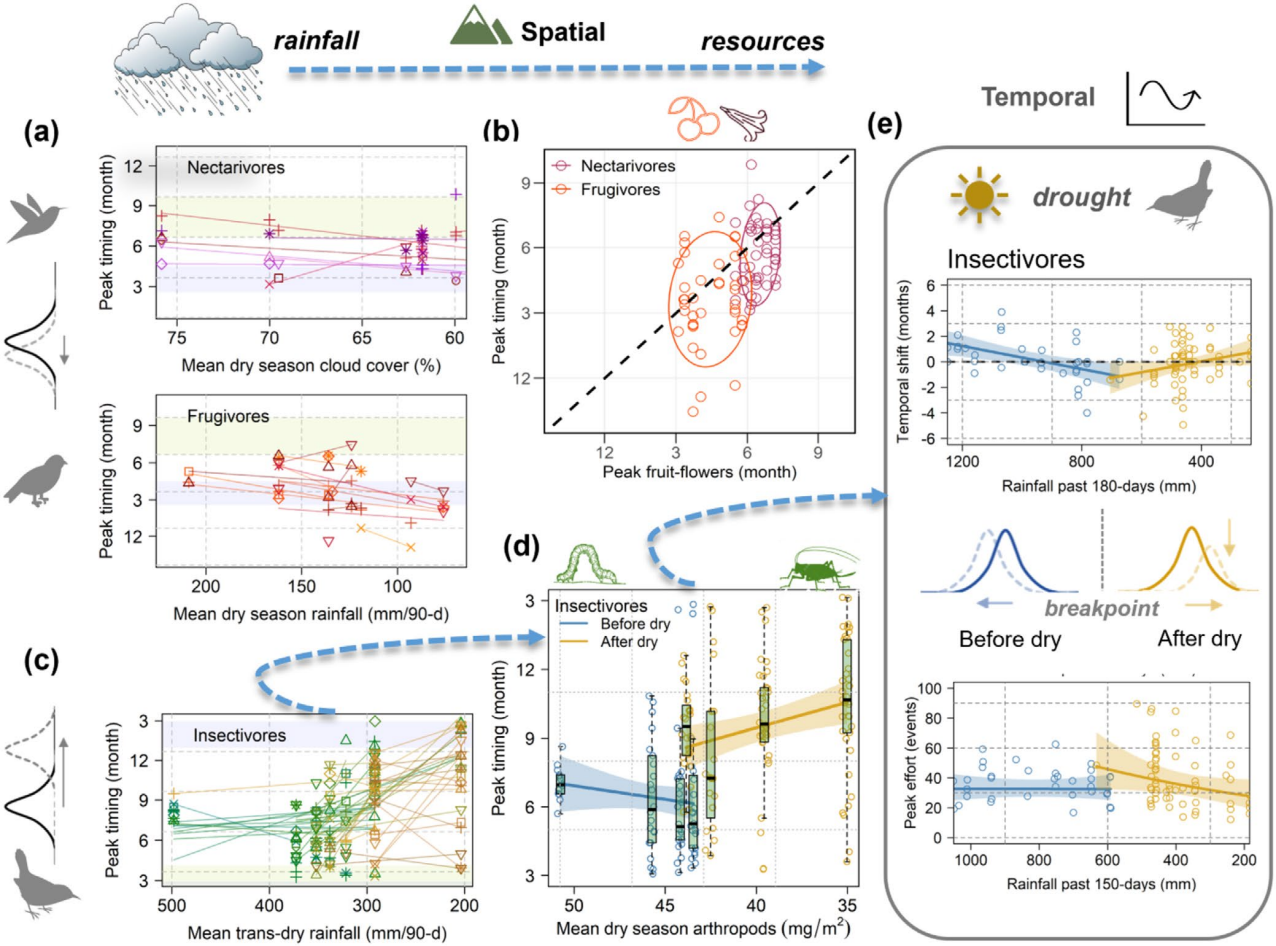
Breeding compartmentalized by diet varied with rainfall‐mediated resources. (a) Across elevation and rainfall gradients, nectarivores [*N* = 50 pops., 24 spp.] nested at the beginning of the dry season (tan), whereas frugivores [*N* = 55 pops., 23 spp.] centered on the wet season (blue). Within dry/wet seasons, weak spatial shifts in timing were explained by changes in dry season cloud cover and rainfall, respectively; *y*‐axes shifted to show linear relationships. (b) Consistent with hypothesized rainfall constraints, nectarivores clustered with dry season flowers while frugivores were dispersed around wet season fruit; for comparison, ellipses enclose 80% CI with dashed 1‐to‐1 line shown in black. (c) Insectivores and omnivores [*N* = 175/21 pops., 77/9 spp.] were locally seasonal while spatial shifts in peak timing were predicted by changes in transition dry season rainfall. (d) Insectivore nesting shifted between transition seasons at a threshold in dry season arthropod biomass. (e) During drought, insectivore nesting shifted away from the dry season, 1 month earlier or later in drier years. Long‐term reductions in rainfall, which extended the dry season, magnified short‐term effects of drought, and when insectivores nested after a stronger dry season, reproductive effort was reduced during dry years, although populations that nested before the dry season did not change [*N* = 97, 42 pops., 22 spp.]. Points in spatial figures (a–d) represent local populations modeled over 5 years; 55%–63% of species connected by lines spanned > 1 landscape. Points in temporal figures (e) represent annual differences relative to the mean timing of local populations.

We used confirmatory path analysis to relate avian phenology to climate and resources allowing us to identify linkages across trophic levels (Figure 4, Tables S7 and S17). We fit models using *d*‐separation tests (Shipley 2013) in the R package piecewiseSEM v2.3.0 (Lefcheck 2016) to select a combination of direct effects of topography on climate and resources, as well as indirect plus direct effects of climate‐mediated resources on birds. Input variables were standardized prior to analysis, and output beta coefficients were standardized by range. To reduce issues with collinearity among variables (Table S1), we focused on one to two key climate variables that related to resources and birds based on the results of our AIC_c_ model selection analyses. Models included phylogenetic and landscape repeated measures as a crossed‐random intercept, which we used in our other analyses. For insectivore shifts, we included direct effects of wet season rainfall to predict peak timing, as the model failed to converge when including transition dry rainfall. To summarize and compare modular responses to changing rainfall based on diet, we used standardized climatic variables from the top AIC_c_ model by trophic guild, which were also supported by SEMs. For species occurring in multiple landscapes, we calculated intraspecific shifts across the gradient based on the key climatic predictor (*x*‐axis). Species were weighted by the proportion of the gradient that they spanned (0–1), which reduced the effect of extreme outliers in which variation was magnified for species at two landscapes with similar climatic conditions (regression estimated unrealistic shifts greater than a calendar year).

## 3 Results

Our systematic sampling included 8225 bird captures with 3849 reproductive or related events, including juveniles and post‐breeding molt, as well as 318 nests. Based on 5 years of systematically sampled data, we modeled phenology for abundant to rare bird species for *N* = 75–301 populations representing phylogenetic diversity of montane bird communities for 40–133 species, 29–91 genera and 13–21 families (Figures S5 and S6, Tables S2 and S3); this included intraspecific spatial variation for 60% of species modeled separately at > 1 landscape. Sample sizes by trophic guild included 22–50 nectarivore, 10–55 frugivore, 37–175 insectivore, and 6–21 omnivore populations. The three most common species captured in mist‐nets included a nectarivore (Trochilidae: *Adelomyia melanogenys* , Speckled Hummingbird) with 536 captures at six landscapes, a frugivore (Tyrannidae: *Mionectes striaticollis* , Streak‐necked Flycatcher) with 503 captures at six landscapes, and an insectivore (Parulidae: *Myiothlypis coronata* , Russet‐crowned Warbler) with 959 captures at eight landscapes.

### 3.1 Bird‐Plant Consumers: Breeding Timed to Regional Dry Season Flowers or Wet Season Fruit

#### 3.1.1 Spatial Variation

At regional scales, nectarivores and frugivores (17% of the community each) nested in different rainfall seasons (*N* = 105 pop., *t* = 3.7, *p* < 0.001). Breeding peaks of nectarivores clustered at the beginning of the dry season, whereas frugivores centered breeding on the wet season (Figure 4a). Across species and families, nectarivores and frugivores timed breeding to community‐wide floral and fruiting maxima (Figure 4b). Consistent with mechanistic hypotheses for seasonal rainfall constraints, bird‐pollinated flowers were most abundant during the dry season, whereas bird‐dispersed fruit were most abundant during the wet season (Figure S3). We found no relationship with elevation despite diverse and changing plant communities across elevations, with > 150 interacting bird‐plant species identified in 33 families and 42 genera (Table S8).

**FIGURE 5:**
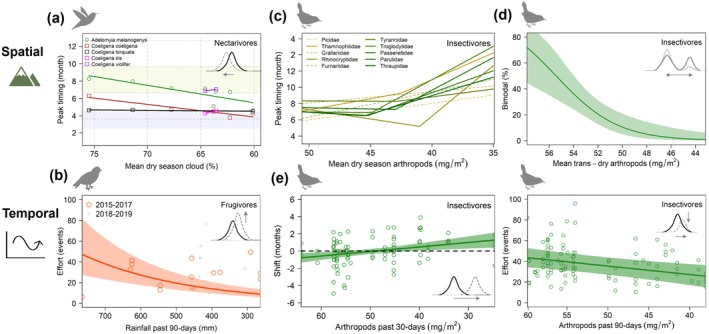
Spatiotemporal links to rainfall and resources by trophic guild. (a) For nectarivores, forest hummingbird genera (Trochilidae) staggered breeding along a gradient of dry season cloud cover; points represent spatial models [*N* = 20 populations]. (b) For frugivores, reproductive effort increased during wet years with no apparent change in timing. Rainfall explained > 30% of the variation but fit the data poorly when including sampling effort at different times of year (gray symbols); points represent temporal models [*N* = 15 populations]. (c) For insectivores, spatial non‐linear breakpoints (solid lines) were repeated across multiple families compared to only three linear models (dashed lines); phenological breakpoints from segmented regression. (d) Bimodal breeding, including peaks in different seasons, increased in insectivore populations at landscapes with greater arthropod biomass during the transition to the dry season. (e) Interannual variability in timing and effort of insectivores related linearly to arthropod biomass at different lag times; points represent temporal models [*N* = 97, 42 populations, 22 species].

Although at regional scales peak timing occurred within restricted rainfall windows, nectarivore and frugivore populations showed weak spatial shifts related to local dry season cloud cover and rainfall, respectively (*R* ^2^ = 8%–30%, Figure 4a). For nectarivores tightly clustered around floral resources (Figure 4b), intraspecific spatial shifts were apparent in two common hummingbird genera (Trochilidae) that were consistently offset and shifted timing in tandem relative to each other across the same climatic gradient (staggered pattern): short‐billed *Adelomyia melanogenys* nested on average 2.2 months (CI 1.2–3.2) later than long‐billed *Coeligena* spp. ( *C. coeligena* , *C. torquata* , *C. iris* ) (Figure 5a). Few frugivores spanned > 1 landscape, and peaks were less tightly clustered around resources than nectarivores (Figure 4b) either because of spatial aggregation of fruiting resources, a higher proportion of insects consumed, or greater resource segregation (Jordano et al. 2003; Wilman et al. 2014; Blendinger et al. 2015; Bender et al. 2018). Especially for bird‐plant mutualists, resource specialization based on trait matching (e.g., longer bills access longer corollas or gape size limits consumption of large seeds) could contribute to fine‐scale temporal compartmentalization (Jordano et al. 2003).

#### 3.1.2 Temporal Variation

**FIGURE 6:**
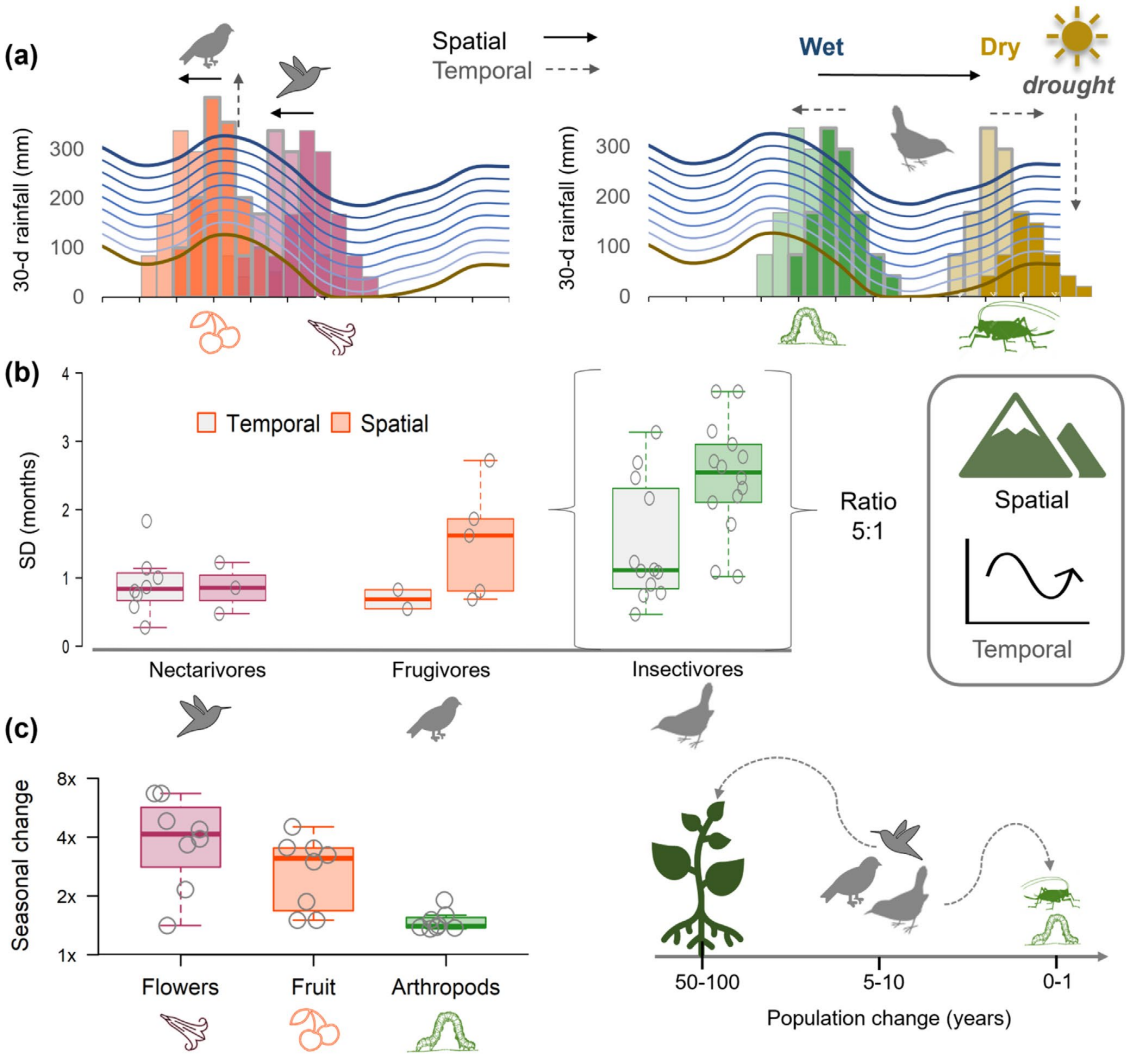
Changing rainfall affects bird‐insect consumers before bird‐plant consumers. (a) Across the same climatic gradient, insectivore phenology shifted abruptly between seasons, whereas nectarivore and frugivore phenology shifted weakly within seasons; solid arrows indicate spatial shifts. Dashed arrows temporal shifts. (b) Limited spatiotemporal variation (SD = standard deviations) of nectar‐fruit consumers contrasted with insectivorous diets, which varied spatially up to 5 months in < 100 km despite temporal variation of only 1 month. (c) Spatiotemporal variation of different trophic guilds was consistent with reduced change in seasonal resources; abundance on a log scale for flowers/fruit, whereas arthropods were measured as a change in biomass. At higher trophic levels, phenological adaptation to a changing climate depends on the rate of population change (generation length, longevity), as small insects responded dynamically to wet and dry extremes, whereas longer‐lived plants access deeper soil moisture through their roots.

Similar to limited spatial variation, there was also limited interannual variation in timing of nectar‐fruit consumers (Figure 6b), and population‐level standard errors over > 3 years averaged ±0.5 months (CI 0.3–0.7) [*N* = 29 pops.]. For frugivorous birds [*N* = 15, 10 pops.], reproductive effort increased 39% (CI 25–56) during wet years with greater wet season rainfall, although sample sizes were small, and model fit depended on the timing of sampling which was challenging during the rainy season (Figure 5b). Despite weak spatial shifts, nectar‐fruit consumers timed breeding to seasonal flowering and fruiting maxima consistent with prevailing patterns across the tropical Andes (Kessler‐Rios and Kattan 2012; Weinstein and Graham 2017) and matching regional timing of wet and dry seasons, which varied spatially by < 2 weeks across the region (Newell et al. 2022a).

### 3.2 Bird‐Arthropod Consumers: Asynchronous Breeding Explained by Dry Season Insects

#### 3.2.1 Spatial Variation

At small spatial scales in low‐latitude mountains, we documented multi‐month differences in breeding seasons of insectivores (66% of the bird community). Although at regional scales insectivores and omnivores appeared to nest throughout the year, timing, in fact, varied locally related to topographically driven changes in seasonal rainfall magnitude (Figure 4c). Asynchronous breeding seasons were explained by local variation in rainfall accumulation during the transition to the dry season (mean 90‐day rainfall 450–200 mm), which decreased with latitude (*AICc w*_*i*_ = 66%, 33%), but not elevation, annual rainfall, or timing of wet and dry seasons. Insectivores nested during the transition to the dry season at the wettest landscapes, whereas they nested during the transition to the wet season at the driest landscapes, an offset of 4.9 months (CI 2.4–7.5, *R* ^2^ = 18%–41%) in < 100 km. Omnivores were similar and grouped with insectivores (Figure S7). This abrupt shift in breeding resulted from lagged effects of transition rainfall (Apr–Jun) on dry season arthropods (Jul–Aug) as mean biomass shifted from dry‐to‐wet season maxima (Figure 3). Climate‐mediated shifts in phenology were predicted by our published biomass model based on dynamic response of foliage arthropods to wet and dry extremes using 5 years of intensive field sampling, in situ rain gauges, and 50 years of regional rainfall (Newell et al. 2023a).

Long‐term reductions in dry season arthropods (e.g., biomass normals) created a rainfall‐driven resource threshold where insectivore populations shifted breeding abruptly between seasons at an invisible climatic barrier (Figure 4d). Thresholds occurred at dry season resource minima of 43 dry mg/m^2^, a hygric limit which implies energetic constraints on reproduction (e.g., to support developing young or juvenile survival). A breakpoint, in which breeding shifted away from the dry season (either 1–2 months earlier or later at drier sites), provided a better fit than a linear relationship with rainfall or arthropods alone, especially for less common species (*R* ^2^ = 27%–43%). Segmented regression showed repeated breakpoints in multiple insectivore families where breeding of local populations shifted abruptly between rainfall transition seasons (Figure 5c). Intraspecific spatial shifts for populations of the same species at different landscapes were evident in > 30 species (Figure 4c), including both suboscines and oscines (Figures S14 and S15).

As breeding shifted locally across low‐latitude mountains, the occurrence of a smaller secondary peak increased, and > 20% of insectivore populations split reproduction between different seasons (bimodal breeding) (Figure 6d, Table S5). Primary and secondary peaks occurred during wet and dry season transitions, and bimodality was best explained by mean arthropod biomass during the dry season transition (*R* ^2^ = 11%). Overall, reductions in seasonal climatic extremes resulted in greater year‐round breeding at two transitional landscapes with weak opposing effects of reduced wet season rainfall and greater dry season cloud cover.

#### 3.2.2 Temporal Variation

Spatially asynchronous breeding seasons of insectivores remained timed based on the long‐term magnitude of seasonal rainfall, and spatial to interannual variability occurred at a ratio of 5:1 months (Figure 6b). Local breeding shifted ±1.0 months (CI 0.4–1.7) earlier or later in drier years [*N* = 97, 42 pops.], depending on the timing of the main breeding season, either before or after the dry season (e.g., breeding shifted away from the dry season) (Figure 4e, Table S6). Overall, insectivore nesting varied linearly with abundance of insect resources during each transition season (Figure 5e). Temporal variation of 1 month is consistent with other tropical systems in which bird breeding tracks annual variation in insect resources (Hidalgo Aranzamendi et al. 2019), whereas abrupt phenological disjuncts of 5 months, which we found between nearby communities across a rainfall gradient, were explained by long‐term reductions in dry season resources (e.g., minima instead of maxima; Figure 4e).

Combined spatiotemporal variation resulted in a positive feedback loop in which reproductive timing buffered or magnified short‐term effects of drought depending on the long‐term magnitude of transition dry season rainfall, which extended the dry season (Figure 4e). Specifically, when breeding before a shorter, wetter dry season, insectivores nested earlier during drier years and maintained reproductive effort. In contrast, reproductive effort was reduced 42% (CI 1–66) during drier years when insectivores nested later after a longer, more intense dry season. Temporal variation was explained by lagged effects of rainfall in the previous 5–6 months, although linear relationships with arthropod biomass received greater support (Figure 6e), either 30‐day means for timing or 90‐day means for reproductive effort. Models explained 5%–9% of the variation in the full dataset, and rainfall explained 10%–18% of the variation for earlier breeding before and reduced reproductive effort after the dry season. Omnivores did not follow this pattern, and two common genera (Passerelidae: *Arremon*, *Atlapetes*) were excluded because of poor fit to the data.

### 3.3 Community Synthesis: Modular Trophic Pathways Link Low‐Latitude Food Webs to Changing Rainfall

At small spatial scales in the same ecoregion (Yungas), diet was the only functional model to explain variation in peak timing across low‐latitude mountains (*AICc w*_*i*_ = 100%, Figure S7, Table S4). Across a complex gradient in which rainfall was not correlated with elevation, we found no relationship with elevation for any trophic guild (Figure S8, Tables S13–S15), consistent with our previous findings that arthropod biomass did not change with elevation (Newell et al. 2023a). Diet interacted with the magnitude of local seasonal rainfall, explaining spatial shifts within Andean bird communities and reshuffling the breeding seasons of different trophic guilds (*R* ^2^ = 22%–43%, Figure 6a, Figure S7). At landscapes with a less intense dry season, insectivores nested with nectarivores during the most climatically stable conditions after the rainy season (Newell et al. 2022a), and consistent with breeding seasons in Southeast Asian rainforest (Fogden 1972; Berman et al. 2023). However, at landscapes with a stronger dry season, the main breeding season of insectivores shifted to the transition wet season, consistent with seasonal dry forest and savanna (Poulin et al. 1992; Hidalgo Aranzamendi et al. 2019). Abrupt local shifts of insectivores contrasted with the seasonal stability of nectarivores and frugivores, which timed breeding regionally to nectar‐fruit resources. The strong spatiotemporal variability in reproductive timing of insectivores was consistent with reduced seasonal change in insects, and arthropod biomass increased 1.5× from minima to maxima, whereas the abundance of flowers and fruit increased 3–4× on a log scale (Figure 6c); however, it is important to note that these metrics are not fully comparable.

**FIGURE 7:**
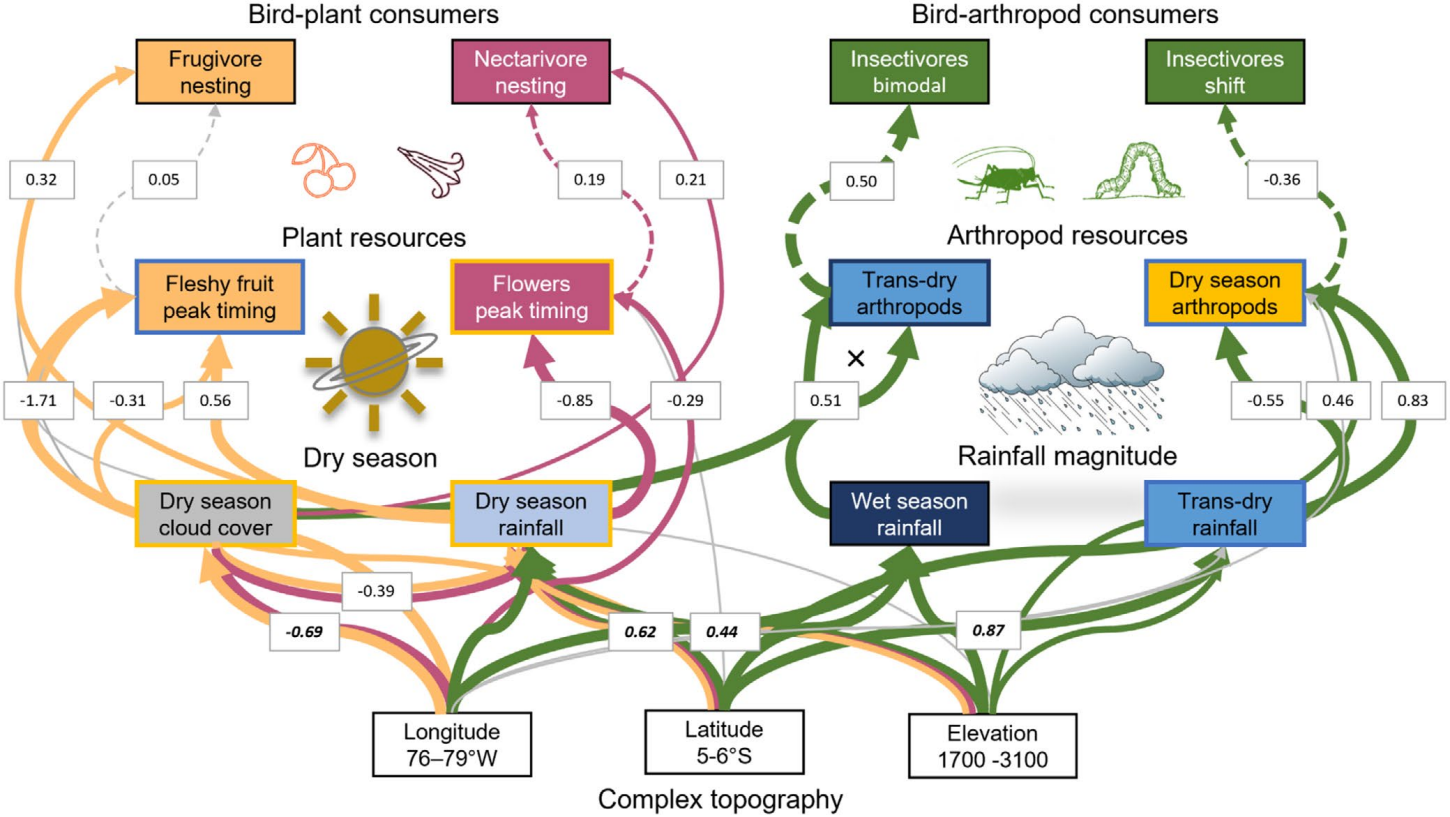
Phenology links multitrophic food webs to a changing climate. Confirmatory path analysis demonstrated cascading effects of changing rainfall from resources to birds. Along the same climatic gradient, modular trophic pathways related to different rainfall constraints on plants versus insects. Bird‐plant consumers varied weakly with subtle changes in dry season cloud cover and rainfall, whereas spatially disjunct breeding of insectivores was explained by long‐term changes in seasonal rainfall magnitude, which reduced dry season arthropods and shifted the timing of biomass maxima (Newell et al. 2023a). Solid arrows indicate direct effects; dashed arrows indicate indirect effects with non‐significant pathways shown in gray. Arrow width indicates the strength of different drivers based on path coefficients standardized by range; univariate correlations for rainfall and topography are shown in bold italics.

Confirmatory path analysis supported coordinated effects of rainfall based on different energetic pathways as reproductive timing of consumers hinged on ecological constraints limiting their resource base. Local climatic drivers differed among trophic guilds (Figure 7, Table S7): insectivores showed strong linkages from transition dry season rainfall to insects to birds (insects: *β*_std_ = 0.30, *t*_6_ = −9.6, *p* < 0.001, insectivores: *β*_std_ = 0.48, *t*_86_ = −5.3, *p* < 0.001), whereas timing of nectarivores and frugivores related weakly to dry season cloud cover and rainfall consistent with irradiance influencing plant phenology in the absence of water stress (Van Schaik et al. 1993). We found strong support for timing differences between nectarivores and frugivores related to food resources and dry season rainfall (food: *β*_std_ = 0.43, *t*_42_ = 3.81, *p* < 0.001, rain: *β*_std_ = 0.30, *t*_42_ = 2.38, *p* = 0.02). However, based on monthly sampling, limited spatial variation within each trophic guild did not clearly link to resource timing, perhaps because of finer temporal scales and/or resource specialization. Across models, there were also differences between marginal and conditional fit, indicating random variation among landscapes (Table S7).

**FIGURE 8:**
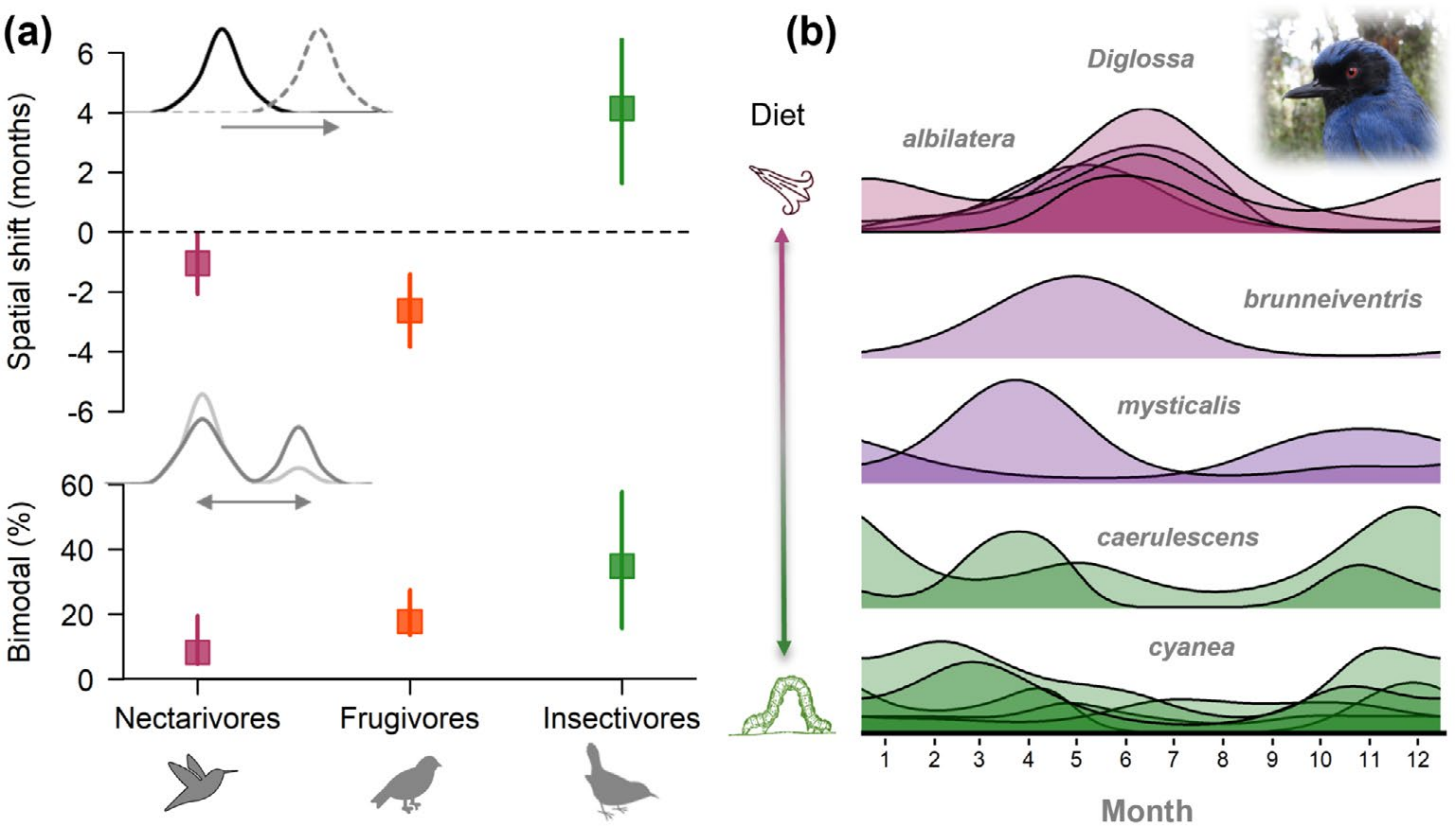
Strong support for modular responses to changing rainfall across trophic levels. (a) Multiple lines of evidence showed that insectivorous bird communities adapted spatially and temporally to changing rainfall magnitude before related species consuming nectar or fruit, including the occurrence of bimodal peaks in different seasons. Error bars indicate 95% CI scaled to equal sample size [*N* = 12–18, 12–15, 47–77 spp.]. (b) Resource‐based adaptations were evident in flowerpiercers (Thraupidae: *Diglossa*) as phenology shifted with dietary transitions. Species that primarily consume nectar nested at the beginning of the dry season (purple), whereas bimodal breeding was common in larger, more insectivorous species (green). Photo credit: Felicity Newell, FLMNH.

Overall, multiple lines of evidence (population‐level asynchrony, bimodal breeding, temporal variability) show compartmentalized responses to climate of different consumer‐resource blocks across spatiotemporal scales (Figures 6b and 8a). Comparing spatial shifts, we estimated that insectivores responded strongly to long‐term changes in seasonal rainfall magnitude with variation 2–8× greater for insectivores compared to nectar‐fruit consumers. Although we detected bimodal breeding in all trophic guilds, the probability of distinct peaks > 4 months apart increased with the percentage of insects in the diet, and insectivores were 7× more likely to nest bimodally than nectarivores (*R* ^2^ = 7%, Figure 8a). Related flowerpiercer species (Thraupidae: *Diglossa*) demonstrate how diet underlies shifts in reproductive timing, and smaller species that primarily consume nectar nested at the beginning of the dry season at the same time as hummingbirds (Trochilidae), whereas larger species that consume more insects nested bimodally with local insectivores (Figure 8b). Asynchrony and bimodality are not restricted to this region, and have been documented in montane species in Ecuador, Colombia, and Mexico (Schondube et al. 2003; Moore et al. 2005; Greeney 2010; Castaño et al. 2023).

## 4 Discussion

Concurrent multitrophic datasets at the landscape scale demonstrate the uniqueness of phenology at low latitudes unconstrained by seasonal changes in day length and temperature. Across nearby mountains with differing rainfall, we documented localized breeding seasons of insectivores offset by up to 5 months. Here, within 5°–6° S of the equator, breeding seasons did not vary with elevation nor relate to leaf‐flush (Newell et al. 2023a). These results contrast with previous studies (Boyle et al. 2016) and show that the well‐documented pattern that breeding seasons are attenuated at higher elevation likely varies with latitude. Thus, phenology did not vary with mean annual temperature, lacking seasonal differences in temperature that delay warming at higher elevations. With limited seasonal variation in daylength (45 min), temperature (< 2°C), or leaf‐flush (≤ 35%), seasonal variation in rainfall (≥ 75%) became the primary driver of bird‐insect phenology across elevations (Newell et al. 2023a). The magnitude of phenological variation we found was 10–30× greater than at higher latitudes, where nest initiation dates often vary spatially 3 days per degree latitude (James and Shugart 1974) and temporal shifts of 3–5 days are a common response to warming, including evidence for trophic mismatch (Youngflesh et al. 2023). Collectively, our results demonstrate a unique contextual framework for food web phenology at low latitudes. Although consistent with the diet‐breeding hypothesis proposed by Skutch (1950) that phenology links to rainfall‐mediated food resources in the tropics, our results highlight the complexity of how food webs adapt to a changing climate around the globe. Moreau (1950) suggested that avian nesting varied extensively within 10° of the equator, and further work is needed to examine how the role of rainfall changes as seasonal variation in day length and temperature becomes more pronounced.

Phenology indicated a critical point at which water stress differed for insectivores, contrasting with the seasonal stability of nectar‐fruit‐based diets. At a dry season tipping point, small changes in rainfall resulted in large changes in phenology, and the main breeding season of multiple insectivore species shifted spatially between rainfall transition seasons. In the expected pattern, nectarivores and frugivores timed breeding to resource maxima, whereas abrupt phenological shifts of insectivores were explained by dry season resource minima. In the absence of water stress, plant‐based food webs linked to rainfall seasonality including solar insolation (Newell et al. 2023a), whereas insect‐based diets responded strongly to local rainfall magnitude resulting in breeding season reversal across wet‐to‐dry gradients. Locally variable montane breeding seasons found in this study show that previous models predicting that birds initiate breeding as resources increase with returning rain (Quintero et al. 2014) primarily apply to seasonally limited higher latitude or arid systems (Poulin et al. 1992; Hidalgo Aranzamendi et al. 2019).

We suggest differences in phenology among trophic guilds relate to a combination of rainfall‐driven resource thresholds underlying the water stress dynamics of plants versus insects, combined with rates of population change tied to body size (e.g., generation length, longevity) (Figure 4c). Longer lived plants access deeper soil moisture through their roots, and although perhaps less immediately susceptible to dry conditions, extreme water stress may be lethal. In contrast, small arthropods responded dynamically to rainfall extremes at short time‐scales in evergreen montane cloud forest (Newell et al. 2023a). Although interannual variability in breeding tracked short‐term resources, insectivorous birds timed breeding seasons to mean arthropod biomass, which shifted between transition seasons based on the long‐term magnitude of seasonal rainfall. Strong spatial variation, which was 5× temporal variation, suggests that birds were not adapting the timing of breeding at short time scales. The consequence of asynchronous reproduction, in which populations differ in timing at local scales, remains unknown. Some evidence suggests differences in reproductive seasonality contribute to genetic divergence in tropical birds (Moore et al. 2005; Quintero et al. 2014). Further work is needed to examine the role of reproductive asynchrony in population isolation compared to other dispersal‐limiting factors such as topography and distance.

In a positive feedback loop, phenological asynchrony magnified the short‐term effects of drought in drier landscapes. For insectivores, communities that nested before the dry season adapted to dry conditions by breeding earlier, whereas communities that nested after the dry season reduced reproductive effort during dry years. Thus, long term reductions in rainfall magnitude—which intensified and extended the dry season via effects on arthropods (Newell et al. 2023a)—shifted breeding to coincide with ENSO‐related drought in the second half of the year. Based on recaptures of the same individual, we believe reproductive effort was reduced because females failed to come into reproductive condition, skipping breeding during dry years similar to arid‐system species (Grant et al. 2000) presumably related to limited food (O'Brien and Hau 2005). Here, we show insectivores are strongly affected by short‐term drought as dry conditions rapidly reduce the biomass of arthropod prey (Newell et al. 2023a). This is consistent with previous research showing that tropical birds reduce reproductive effort during drought (Grant et al. 2000; Martin and Mouton 2020), and, in fact, most species in these studies consume a high percentage of insects (Wilman et al. 2014). Thus, localized breeding season reversals could contribute to differing population‐level responses to extreme wet/dry years, with magnified effects of drought increasing variability in drier systems.

These results have important implications for the effects of climate change in tropical systems. In our system, the prevailing hypothesis that the “wet‐get‐wetter and dry‐get‐drier” due to intensification of the hydrological cycle (Held and Soden 2006) has resulted in greater seasonal extremes, including both increasing wet and decreasing dry season rainfall, as well as greater interannual variability during ENSO‐related drought (Newell et al. 2022a). Our results show that arthropods and birds were both negatively affected by short‐term periods of drought and heavy rain, which might increase the spatiotemporal variability of insectivore populations. Long‐term changes in climate, which reduce intermediate rainfall and increase seasonal extremes, are predicted to reduce population viability in both wet and dry systems (Newell et al. 2023a). For example, in the drier eastern Amazon in Brazil, drought increased bird mortality (Wolfe et al. 2025), potentially explaining long‐term population declines of rainforest birds (Stouffer et al. 2021). In contrast, in the wetter western Amazon in Ecuador, decreasing populations of some rainforest insectivores could relate to increasingly wet ENSO events (Blake and Loiselle 2024), although in southern Perú rainforest communities appear unchanged (Martínez et al. 2023). Additionally, the length of the dry season explained the demography of rainforest birds in Panama (Brawn et al. 2017) while in montane systems in the Afrotropics, population declines were explained most directly by temperature (Neate‐Clegg et al. 2021). In our system, high humidity buffered variation in temperature, while with year‐round rainfall, rainfall magnitude was more critical than the duration of dry conditions. Across the tropics, increasingly extreme ENSO events characterized by both drought and heavy rain (Yun et al. 2021; Petry et al. 2025) have been linked to decreasing arthropod diversity and function (Sharp et al. 2025) while changes in biomass are likely to have cascading effects on insectivorous vertebrates (Newell et al. 2023a). Because of their rapid response and sensitivity to change, insectivorous food webs may provide advance warning, whereas plant‐based food webs might be affected by extreme drought, potentially increasing the risk of ecosystem collapse. The long‐term consequences of increasing rainfall extremes will depend on the magnitude, duration, and frequency of both drought and heavy rainfall, as well as the ability of communities to adapt to changing conditions.

At small spatial scales in the same ecoregion, we found consumer‐resource blocks rotated phenology along different rainfall axes as ecosystems transitioned along a gradient of changing rainfall magnitude (Figure 1). Ecological constraints of rainfall on different taxonomic groups partitioned regional food web phenology via modular trophic pathways that responded quasi‐independently to changing rainfall. These vertical modules align with the generalized concept of trophic guilds, although further work is needed to examine seasonal variation in diet as well as a broader range of omnivorous species with diverse diets. Further work is also needed to examine if and how reversed seasonality applies in the lowland tropics. In drier systems, bird communities breed during the rainy season (Poulin et al. 1992; Hidalgo Aranzamendi et al. 2019), whereas rainforest communities nest during the dry season in Southeast Asia (Berman et al. 2023), although breeding may be extended or even aseasonal in drier rainforest in the Brazilian Amazon (Stouffer et al. 2013). Across the tropics, modular phenology at low latitudes has potential implications for ecosystem stability (May 2001; Grilli et al. 2016). Interconnected consumer‐resource blocks begin to untangle Darwin's description of food webs as the “tangled bank.” This block‐response shows how climate‐ mediated energy flow underlies reproduction, which can be considered as providing the raw material for natural selection to act upon. Further work is needed to link phenology to population dynamics and examine the effects of interannual variability on species with different diets.

Overall, in tropical mountains, the main breeding season of insectivores centered on intermediate rainfall, especially during transitional seasons (Figure 4a). This supports a rainfall continuum in which both drought and heavy rain limit species' ecology (Boyle et al. 2020). In montane cloud forest, we estimated optimal rainfall to be between 100 and 150 mm month^−1^, although at hotter, lower elevations, high rates of evapotranspiration require greater monthly rainfall to maintain climatic water balance. People in Perú describe seasonality based on rainfall, and *campesino* farmers in the mountains refer to wet and dry seasons as *el invierno* and *el verano*, or winter and summer, respectively. The idea that intermediate rainfall brings a tropical spring with optimal conditions for raising young suggests that increasing wet and dry extremes could destabilize insectivore populations (Newell et al. 2023a). We found strong evidence for long term phenological adaptation as insectivore breeding shifted abruptly between seasons and wet tropical systems became drier and vulnerable to drought. Ultimately, the intensity of the dry season drives plant community turnover along climatic gradients, forming the basis of Köppen climate classifications in the tropics (Beck et al. 2018). In this region, evergreen cloud forests transition to seasonally deciduous dry forest (Figure 2) in narrow interandean valleys below 2000 m to the south and west, where cloud forest sites were located near dry forest. As rainfall magnitude changes, shifts in plant biomes may be preceded by an accumulation of subtle changes, such as changes in phenology. Our results show how rainfall constraints limit ecological function across food webs, and at low latitudes, abrupt nonlinear shifts in phenology provide an early warning for ecosystems approaching transition points with potentially reduced resilience to climatic extremes.

## Author Contributions

Felicity L. Newell conceived and designed the study with advice from Scott K. Robinson. Felicity L. Newell and Ian J. Ausprey collected the data, and all authors contributed funding. Felicity L. Newell analyzed the data and wrote the manuscript with significant contributions from Ian J. Ausprey and Scott K. Robinson.

## Funding

This work was supported by the University of Florida, Association of Field Ornithology, and American Ornithological Society.

## Conflicts of Interest

The authors declare no conflicts of interest.

## Supporting information


**Data S1:** gcb70790‐sup‐0001‐Supinfo.pdf.

## Data Availability

Weather data summarized from (Newell et al. 2022b) https://doi.org/10.6084/m9.figshare.18543167. Arthropod data and biomass models from (Newell et al. 2023b) https://doi.org/10.6084/m9.figshare.19107188. Avian phenology, resources, and climate datasets, as well as scripts used to generate figures available on Figshare (Newell et al. 2024) https://figshare.com/s/ff7b1e44e4346d65b1ec.
