# Supplementary material for: Changing Rainfall Drives Locally Asynchronous Reproduction of Tropical Birds via Modular Trophic Pathways
Source: Glob Chang Biol. 2026 Mar 26;32(3):e70790. doi: 10.1111/gcb.70790 (PMC13019422; doi:10.1111/gcb.70790)
Supplement: Supplementary file 1 — Data S1: gcb70790‐sup‐0001‐Supinfo.pdf. [file GCB-32-e70790-s001.pdf]

Supporting information for:

Changing rainfall drives locally asynchronous reproduction of tropical birds via modular trophic pathways

Felicity L. Newell, Ian J. Ausprey, and Scott K. Robinson

Email: felicity.newell@tamu.edu or felicity.newell@gmail.com

This PDF file includes:

SI Methods………………………………………………………………… Pages 2–3
Combined breeding model……………………………………. Pages 3–6
SI References………………………………………………… Page 7

SI Figures S1 to S8………………………………………………….. Pages 8–15

SI Tables S1 to S7…………………………………………………….Pages 16–21

SI Extended Figures
Modeling seasonal resources
Figures S9 to S11……………………………………. Pages 22–24
Bird species by taxa and diet
Figures S12 to S15…………………………………... Pages 25–28

SI Extended Tables
Taxonomic scope
Tables S8 to S10……………………………………...Pages 29–33
Model selection results
Tables S11 to S17…………………………….........Pages 34–40

SI Extended Acknowledgements/Collaborators in Perú…………………Pages 41–42

SI Methods

Montane climate in the Andes of northern Perú

Spanning a rainfall gradient from the eastern slopes to interandean valleys, seven cloud forest landscapes were located 10–100 km apart in the department of Amazonas with one landscape in neighboring San Martin. Approximately half of 60 subsites were located in contiguous forest (N=28) and half in forest fragments (N=25) with a few in successional habitat (N=7). Forest fragments primarily ranged from 3–30 ha in size with a surrounding matrix of mixed agriculture and pasture. Similar to fragments, contiguous forest included sampling <500 m from the forest edge, and all sampling was conducted in forest >50 m from a hard edge (pasture or crops). Forests tended to become intermixed with shrubs at higher elevation, often including the presence of bamboo (*Chusquea* spp.) at 2500 m and above. Based on terrestrial ecoregion maps from The Nature Conservancy (TNC), sites represented a single ecoregion, Peruvian Yungas, with the study system located south and east of the Marañon Gap, a well-known biogeographic divide where cloud forest transitions to seasonal dry forest below 2000 m.

Abundance of flowers and fruit

To quantify seasonal abundance of floral and fruiting resources, we combined several approaches to survey plants. Beginning in 2015–2016, we conducted fixed-count point-transects recording plant phenology for the 20 nearest understory shrubs at 5 points on each visit. After we became familiar with common species, we added additional fixed-area point-transects in 2017 to comprehensively quantify seasonal abundance of flowers and fruit. On each landscape-visit we systematically sampled an average of 10 points (range: 5–22 depending on time) located every 30 m along net-lane transects. At each point we recorded all flowers and fruit within a 12.5-m radius. During 104 visits to 7 landscapes over 4 years, we recorded presence/absence of flowers and fruit on 14 331 shrubs at 564 points, as well as phenology of 13 123 trees. In 2017, we added additional fixed-area counts on 51 visits recording 4 025 flowering or fruiting individuals at 562 points. Flowers were detected on 100% and fruit on 92% of visits for fixed-radius transects, whereas observations of 100 shrubs detected flowers on 79% and fruit on 51% of visits. To control for variability in sampling effort, visits were weighted by effort (≥10 points = 1) and plant-based observations contributed a tenth compared to fixed-radius counts. For AIC_c model selection, we used predicted values for Venceremos which lacked fixed-radius counts after 2016, although scaled percentages showed a similar monthly pattern to other sites with flowers peaking after fruit in May–Aug (flowers: 21, 100, 27, 5, fruit: 100, 5, 18, 1); we also ran model selection without Venceremos.

Classification of bird-pollinated and bird-dispersed plants

We applied a syndrome-based approach to classify pollinator species using floral morphology and color combined with information available from the literature as well as field observations. We identified primarily bird-pollinated species based on their tubular shape, and/or bright colors, especially red-to-orange, and we considered flowers of other species used by nectivorous birds to be secondarily bird-pollinated, offering opportunistic resources (Fenster et al. 2004, Rosas-Guerrero et al. 2014, Abrahamczyk and Kessler 2015). We identified primarily bird-dispersed fleshy fruit based on bright colors such as red, blue and blue-black, as well as soft pulp and short peduncles; we considered primarily green fruit, generally harder and larger, to be opportunistically used by birds as well as bats or other mammals (Gautier-Hion et al. 1985,

Wheelwright and Janson 1985, Valenta and Nevo 2020). Initial analysis showed no difference between primarily or opportunistically used plants, and both were included in our analysis. We excluded fruit counted on large canopy trees such as *Ceroxylon* and Lauraceae spp. which would not generally be eaten by small understory frugivores. Fruiting trees were also less consistently seasonal contributing to large, skewed counts when detected.

Mist-netting and banding of cloud forest bird communities

We spent three days at a site during each visit including set-up and take-down of mobile banding stations. On the first day, we installed 20–30 nylon mist-nets (30-mm gauge), generally 12-m in length with a few 6-m or 18-m nets depending on vegetation structure. The objective was to maximize the number of captures, controlling for effort based on net hours scaled to a 12-m net. We ran mist-nets for two consecutive days opening nets at first light and closing them around midday to allow time for travel between sites or other field work. For logistical reasons mist-nets were located either (i) linearly along trails generally parallel to the slope or (ii) in a circular loop so that they could be checked quickly and easily to prioritize bird and human safety. On cold or wet mornings before 9am, net lanes were checked every 15 min and sometimes continuously so that small hummingbirds could be processed quickly; net checks were reduced to every 30 min later in the morning, and nets were closed during rain. To maximize safety, birds were processed rapidly within <1 hour of capture, and banding data were collected or overseen primarily by a single bander with >10 years of experience (FLN processed >50% of birds over 5 years). Experienced banders and trainees learned and processed common species, contributing important observations during 1–2 seasons. Banding assistants also helped to collect >10 000 field samples, including feather, fecal, and blood samples deposited at the Florida Museum of Natural History. All banding assistants managing and checking mist-nets were trained and experienced in safe extraction and handling methods according to the North American Banding Council (NABC 2003). Many were part of the Programa de Anillamiento CORBIDI in Perú.

For the cloud forest bird community, we banded 7 422 individuals of 225 species during 43 986 mist-net hours. An additional 1 344 individuals were recaptured on another visit, and 6 041 individuals were identified as adults (~73%). We also made 318 observations of breeding activity or nests. To describe annual cycles, we considered current breeding events as either a brood patch (score 1–3, including ventral feather loss to full vascularization) or cloacal protuberance (score 2–3, moderate to heavy swelling) (Pyle 1997). We considered molting events based on sequentially growing flight-feathers as part of the definitive pre-basic molt in which flight-feathers are generally completely replaced; we also included a few birds initiating their pre-basic molt. When possible, we excluded individuals undergoing the first pre-basic molt (e.g. incompletely ossified skull or hummingbirds with bill striations >10%) which would not have been previously breeding; also timing of the first pre-basic molt appeared to be earlier than the post-breeding molt for some species. During intensive fieldwork at a site, we recorded any observations of breeding activity including active nests, adults carrying material or food, and dependent juveniles (Vickery et al. 1992).

Combined breeding model

Phenological events controlling for effort

Combining all evidence for local population-level reproductive timing, we analyzed monthly counts of current plus backdated reproductive events per population (species-by-landscape) controlling for monthly effort based on captures. To calculate monthly effort, we summed

captures of a species for the current plus previous months in which we might capture juveniles or molting adults which we then merged with backdated breeding counts by month; juveniles and molt were backdated at most 3.5 to 5.3 months prior to capture respectively. Because the probability of detecting previous reproductive events decreases over time, we multiplied previous effort by a fraction of the difference in months which resulted in a similar proportion of reproductive events compared to raw data. We used the following steps to format model inputs: (i) we summed counts of active plus backdated reproductive events by month; (ii) we summed effort for current captures plus a decreasing percentage of backdated captures; (iii) to reduce variability given small sample sizes we applied a centered 3-month running mean to smooth counts (Stouffer et al. 2013); (iv) to calculate monthly event rates we included effort as an offset in the model to output results per 100 individuals; for monthly counts with 1–2 individuals we used an offset of 3 to reduce outlier effects; (v) to control for variable numbers of captures, we weighted monthly rates by the proportion of effort (≥10 captures = 1). Reproductive activity rates were calculated using the following equation:

$$reproductive\ activity\ rate =$$

$$\sum_{month(i)}^{a\,+\,jb\,+\,mb\,+\,O} events\ +\ offset\left(\sum_{month\ (i+i_{n\,\times\,1/dif_n})} effort\right)$$

$$+\,weight\left(\sum_{month\ (i+i_{n\,\times\,1/dif_n})} effort\right)$$

where a = reproductive adult (brood patch or cloacal protuberance); j^b = juvenile backdated to approximate nest initiation dates; m^b = molting adult backdated to approximate nest initiation dates; o = breeding observation during banding effort (backdated if appropriate). Reproductive counts included any observations of nests or breeding adults detected during our regular mist-netting efforts to sub-sites, although we excluded systematic nest searching from hummingbird transects which was only conducted at one landscape in 2017.

Backdating related breeding observations

For phenological phases (phenophases) following reproduction (juveniles, molt), we backdated each observation to approximate nest initiation dates. Based on analysis of interannual variability, we estimated backdating precision at ±0.5 months which in an extremely variable tropical system, allowed us to quantitatively examine the occurrence of reproduction throughout the calendar year at community and landscape scales. Nest initiation dates for juveniles were estimated assuming a constant rate of change based on standard physical aging criteria from the literature (Pyle 1997, Fierro-Calderón and Martin 2007) combined with direct observations from our system. Our protocol for photo documentation was especially important for aging juveniles and allowed us to review data recorded in the field to confirm the extent of juvenile plumage and gape. We did not backdate juveniles with more extensive skull ossification or bill striations because we felt that the rate of change might be less reliable and could not be confirmed by other juvenile characteristics. Juveniles were backdated to approximate nest initiation dates based on the following equation:

$$date_{initiate\ reproductive\ event} = date_{capture\ juvenile} - (ar + c)$$

where a = aging criteria (hummingbirds: a = 60–100% bill striations; passerines: a = skull <50% ossified, score 0–2); r = rate of change (hummingbirds: r = -15% bill striations per 30 days, passerines: r = +1 skull ossification score per 30 days); and c = length of the nesting cycle (hummingbirds: c = 40 days, passerines: c = 35 days).

Adults molting flight feathers were backdated to approximate initiation of molt multiplying the molt score by an estimated rate of feather growth developed from the literature (Rohwer et al. 2009, Silveira and Marini 2012, Rohwer and Rohwer 2013), as well as based on our data. This assumed (i) a constant rate of feather growth, (ii) adults breed prior to the definitive pre-basic molt, (iii) all molting adults previously attempted to breed. Nest initiation dates from progression of molt were estimated based on the following equation:

$$date_{initiate\ reproductive\ event} = date_{capture\ molting\ adult} - (mr + c + p)$$

where m = molt score / max score; r = rate of change (hummingbirds: r = +0.65 per day, passerines: r = +1.3 per day); c = length of the nesting cycle as above; p = post-fledging care prior to initiating molt = 30 days (all species).

Cyclical generalized additive models

To describe reproductive phenology at the population-level, we analyzed input rates for reproductive events (counts) for combined-breeding models using a *poisson* distribution with an offset for effort (log scale). To model annual cycles, we used generalized additive mixed models (GAMMs) in the R package gamm4 v0.2.6 (Wood and Scheipl 2020) which integrates generalized linear mixed models using the Laplace approximation from lme4 (Bates et al. 2015) with additive modeling based on a polynomial smoothing function from mgcv (Wood 2017). We used cyclic cubic regression splines which force the polynomial smoothing function to join at each end describing the calendar year. Selecting the number of knots remains an unresolved issue, and we used the following approach to model phenology. First, we started with automatic knot placement using Generalized Cross-validation (GCV) in mgcv (k = -1). Then, we examined outputs graphically to see if the model effectively balanced simplicity and explanatory power. If the automatic knot function resulted in overfitting (squiggly model), we inspected models with a range of knots (3–12) and visually selected the best fit to the data using the following criteria: (i) for simplicity, we assumed that a unimodal model provided the most parsimonious fit (k = 3), unless other models substantially increased the variance explained; (ii) we maintained peak values within biologically meaningful ranges consistent with the raw data (<100%) (iii) to reduce overfitting, we minimized the number of peaks/knots while maximizing R^2; the majority of the models were fit manually (227/301). Annual cycles were output on a daily time-step as reproductive events per 100 individuals to extract phenology metrics. This resulted in unimodal peaks for the majority of populations (203/301) while the remainder included a second peak (bimodal). Models based on monthly counts summed across years greatly improved fit compared to original analysis of counts summed by landscape-visit (Newell 2021). Species annual cycles were graphed using the R package ggridges v0.5.4 (Wilke 2024). For individual models, see folder ***avian.cycles.graphs.zip*** (Newell et al. 2026).

In our main analysis we pooled data across habitats and years to model spatial variation in phenology across montane landscapes over 5 years which included 94% of the data. To examine any effects of habitat, we ran the same models with populations partitioned by habitat type within the same landscape (Figure S4b). In addition, we ran a temporal analysis modeling phenology by year for each population (Table S2). Because of small sample sizes by year, annual

models were fit using a minimum number of knots (*k* = 3) and we were able to run annual models for 48% of populations. For the year analysis, we used models with >9 captures but no filter for reproductive events because of reduced effort during dry conditions; the year analysis included 48% of the data. For annual analyses, we also examined differences based on timing of sampling. In 2015–2017, we conducted six months of sampling at the same time each year from dry-to-wet season transitions (May/Jun–Nov), whereas in 2018–2019 we focused 3 months of sampling on dry (May–Jul) and wet seasons (Dec–Feb).

Phenology model validation

Combined–breeding models were validated against a subset of populations with sufficient events from a single phase of the annual cycle (Figure S4c). A common approach has been to examine phenology as the proportion of individuals within the population engaged in different activities such as reproduction or molt (phenophases) (Poulin et al. 1992, Stouffer et al. 2013, Berman et al. 2023). To validate our modeling approach across the cloud forest bird community, we compared 40–60% of the output against common species with enough data to model a simple phenophase-occurrence model. We scored individuals *yes/no* for breeding or molt to input a binomially distributed response variable for each activity separately as a function of Julian date (season). We analyzed adult-*only* models for species in which we were able to determine adult age reliably while for other species we used all non-juvenal captures including individuals in the first year of life (first-after-juvenile). The model assumes an equal detection probability (capture) of different phenophases, or at a minimum unbiased detection. For species with large enough sample sizes of a single phenophase, we compared extracted phenology metrics to examine performance of the combined-breeding model. The variance structure of phenophase-occurrence models included a crossed random intercept by landscape and sampling period.
Our model evaluation included comparing peak timing estimated from combined-breeding models to phenophase-occurrence for breeding and molting adults, as well as capture rates of juveniles. Based on *N*=131–189 comparisons across the cloud forest bird community, overall combined-breeding models provided a good fit compared to traditional approaches explaining 57–76% of the variation (Figure S4c). Considering that variation also related to data exclusion (simple models included ~30% of events), we used the combined-breeding model for subsequent analyses which allowed us to integrate multiple sources of data and maximize the number of species across landscapes.

Additional validation included examining backdating, as well as habitat, to support results. For hummingbirds, comparison with nests confirmed timing of breeding (Figure S4a). Hummingbird models were based on backdated observations (juveniles, molt) as breeding characteristics cannot be reliably identified in this group. Models matched combined data for hummingbird nests found opportunistically at different times of year throughout the 5 years of the study [N = 30 nests]; as well as systematic transects searching roadside banks at Levanto [N = 63 nests] conducted separately from other sampling locations in May–Jul 2017. Reproductive timing was also identical in fragmented and contiguous forest (on the 1-to-1 line) with limited evidence for successional effects (Figure S4b). Thus, we pooled data across habitats including 7 successional subsites (8% of captures) which were sampled on the same visit as forest. Although we cannot exclude small-scale effects of habitat within landscapes (days to weeks), on the scale of monthly sampling any effect of fragmentation would be minimal compared to large-scale effects of local climate (≥1 month).

SI References

Abrahamczyk, S., M. Kessler. 2015. Morphological and behavioral adaptations to feed on nectar: how feeding ecology determines the diversity and composition of hummingbird assemblages. Journal of Ornithology 156:333–347.

Bates, D., M. Mächler, B. Bolker, S. Walker. 2015. Fitting linear mixed-effects models using lme4. Journal of Statistical Software 67:1–48.

Berman, L., D. Li, Y. Shufen, M. Kennewell, F. Rheindt. 2023. Bird breeding season linked to sunshine hours in a marginally seasonal equatorial climate. Journal of Ornithology 164:125–138.

Fenster, C. B., W. S. Armbruster, P. Wilson, M. R. Dudash, J. D. Thomson. 2004. Pollination syndromes and floral specialization. Annual Review of Ecology, Evolution, and Systematics 35:375–403.

Fierro-Calderón, K., T. E. Martin. 2007. Reproductive biology of the Violet-Chested Hummingbird in Venezuela and comparisons with other tropical and temperate hummingbirds. The Condor 109:680–685.

Gautier-Hion, A., J.M. Duplantier, R. Quris, F. Feer, C. Sourd, J.-P. Decoux, G. Dubost, L. Emmons, C. Erard, P. Hecketsweiler, A. Moungazi, C. Roussilhon, J.M. Thiollay. 1985. Fruit characters as a basis of fruit choice and seed dispersal in a tropical forest vertebrate community. Oecologia 65:324–337.

Lefcheck, J. S. 2016. piecewiseSEM: Piecewise structural equation modelling in R for ecology, evolution, and systematics. Methods in Ecology and Evolution 7:573–579.

NABC. 2003. Guía de Estudio del Anillador de NorteAmérica. North American Banding Council.

Newell, F. L. 2021. Birds, arthropods, and plants: how rainfall seasonality regulates reproduction across food webs in the Peruvian Andes. PhD Thesis, University of Florida.

Newell, F. L., I. J. Ausprey, S. K. Robinson. 2022. Spatiotemporal climate variability in the Andes of northern Peru: Evaluation of gridded datasets to describe cloud forest microclimate and local rainfall. International Journal of Climatology 42:5892–5915.

Newell, F. L., I. J. Ausprey, S. K. Robinson. 2023. Wet and dry extremes reduce arthropod biomass independently of leaf phenology in the wet tropics. Global Change Biology 29:308–323.

Newell, F. L., I. J. Ausprey, S. K. Robinson. 2026. Data from: Changing rainfall drives spatially asynchronous nesting of tropical birds via modular trophic pathways. Figshare Digital Repository.

Poulin, B., G. Lefebvre, R. McNeil. 1992. Tropical avian phenology in relation to abundance and exploitation of food resources. Ecology 73:2295–2309.

Pyle, P. 1997. Identification guide to North American birds. Slate Creek Press, Bolinas, CA.

Rohwer, S., R. E. Ricklefs, V. G. Rohwer, M. M. Copple. 2009. Allometry of the duration of flight feather molt in birds. PLoS Biology 7:e1000132.

Rohwer, V. G., S. Rohwer. 2013. How do birds adjust the time required to replace their flight feathers? The Auk 130:699–707.

Rosas-Guerrero, V., R. Aguilar, S. Martén-Rodríguez, L. Ashworth, M. Lopezaraiza-Mikel, J. M. Bastida, M. Quesada. 2014. A quantitative review of pollination syndromes: Do floral traits predict effective pollinators? Ecology Letters 17:388–400.

Shipley, B. 2013. The AIC model selection method applied to path analytic models compared using a d-separation test. Ecology 94:560–564.

Silveira, M. B., M. Â. Marini. 2012. Timing, duration, and intensity of molt in birds of a Neotropical savanna in Brazil. The Condor: Ornithological Applications 114:435–448.

Stouffer, P. C., E. I. Johnson, R. O. Bierregaard Jr. 2013. Breeding seasonality in central Amazonian rainforest birds. The Auk 130:529–540.

Valenta, K., O. Nevo. 2020. The dispersal syndrome hypothesis: How animals shaped fruit traits, and how they did not. Functional Ecology 34:1158–1169.

Vickery, P. D., M. L. Hunter, J. V. Wells. 1992. Use of a new reproductive index to evaluate relationship between habitat quality and breeding success. The Auk 109:697–705.

Wheelwright, N. T., NC. H. Janson. 1985. Colors of fruit displays of bird-dispersed plants in two tropical forests. The American Naturalist 126:777–799.

Wilke, C. O. 2024, January 23. Ridgeline Plots in “ggplot2” [R package ggridges version 0.5.6]. Comprehensive R Archive Network (CRAN). https://CRAN.R-project.org/package=ggridges.

Wood, S. N. 2017. Generalized additive models: An introduction with R. 2nd Edition. Chapman & Hall/CRC press, New York, NY.

Wood, S., and F. Scheipl. 2020. gamm4: Generalized additive mixed models using “mgcv” and “lme4”. R package version 0.2-6.

SI Figures

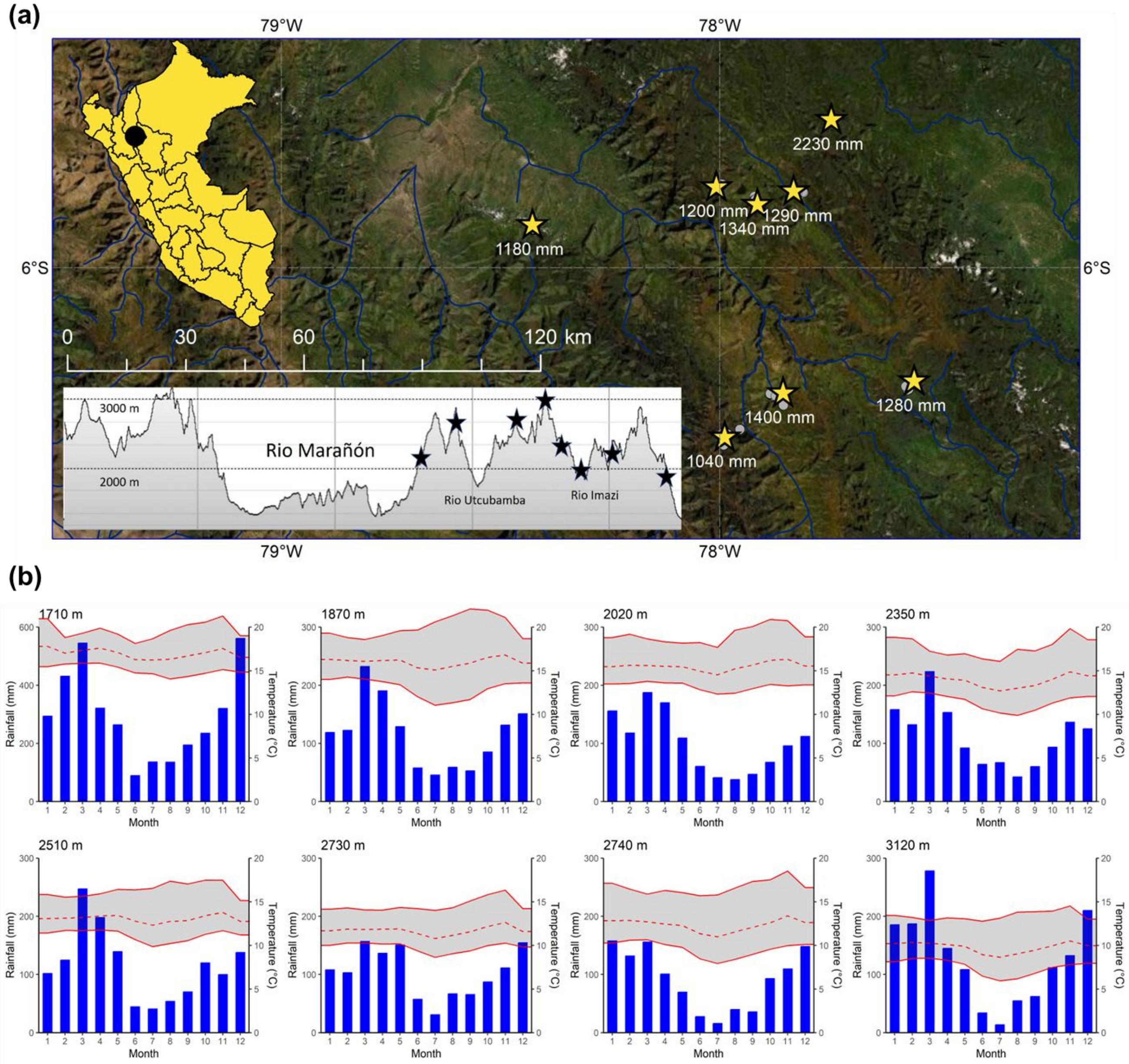

Figure S1. Elevation and rainfall gradient in the Andes of northern Perú

(a) Complex elevational gradient at 5–7°S used as a natural experiment to examine effects of changing rainfall on avian reproductive phenology using a space-for-time substitution. Landscapes spanned a 1700–3100 m elevation and 1000–2500 mm rainfall gradient (labels show 30-year annual means). Weather data were collected for each landscape from 2015–2019 using in situ tipping-bucket rain gauges (yellow stars). Multitrophic data on birds, arthropods, and plants were collected at 3–9 subsites per landscape (gray points). The horizontal cross section shows landscapes by elevation across four watersheds which reduced effects of orographic rainfall (e.g. rainfall-elevation correlations), **(b)** Climographs for each landscape; blue bars show 30-day rainfall accumulation and gray polygons show minimum, mean, and maximum temperatures for loggers located inside the forest, 2015–2019 (Newell et al. 2022).

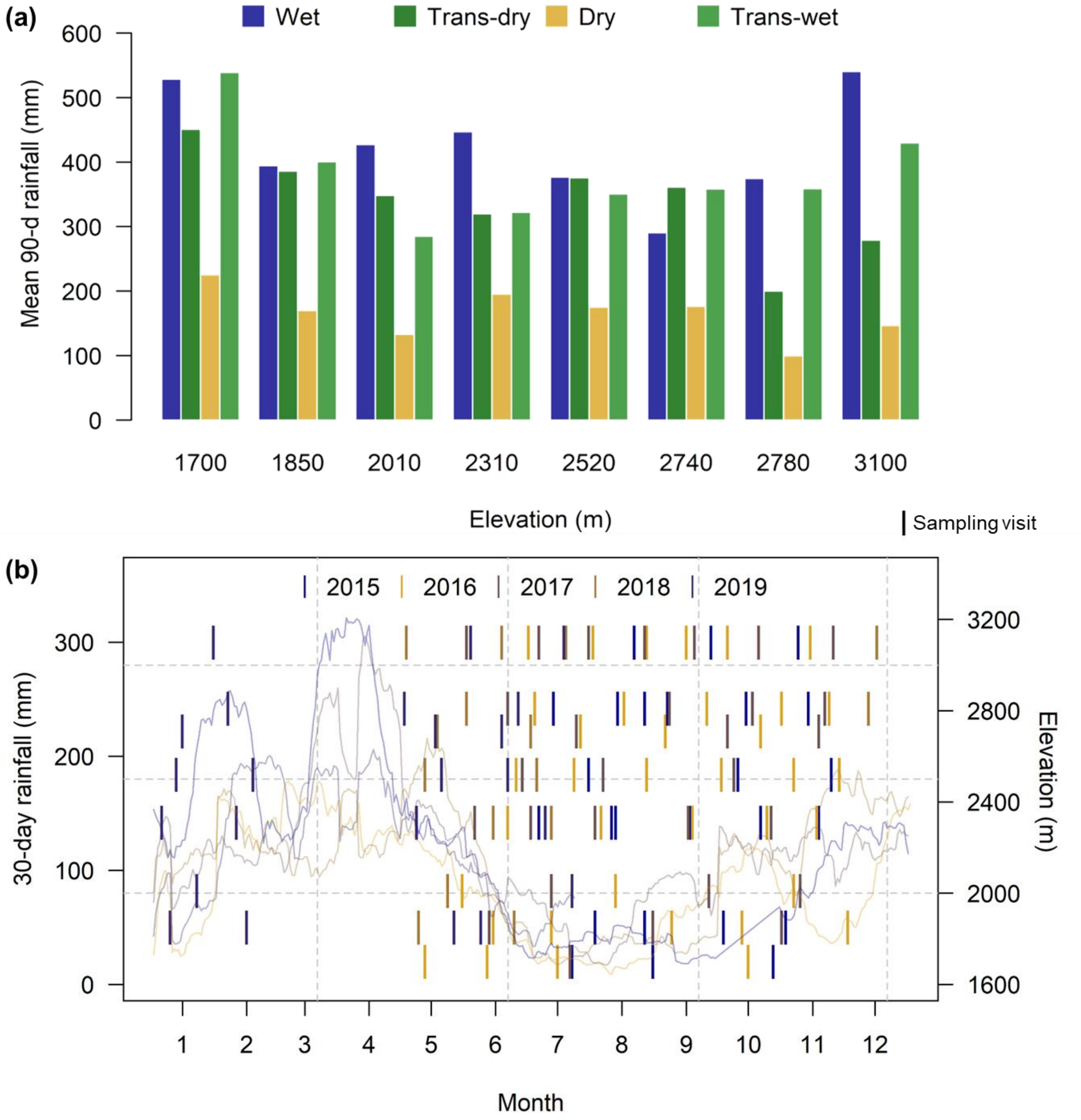

Figure S2. Seasonal sampling design across elevation and rainfall gradients

(a) Mean seasonal rainfall accumulation (magnitude) by elevation for wet (Jan-Mar), transition-dry (Apr-Jun), dry (Jul-Sep), and transition wet (Oct-Dec) seasons. Across a complex elevation gradient, rainfall was not correlated with elevation (R = 0.13); our modeling approach examined how seasonal rainfall magnitude explains spatial shifts in phenology (Table S15) **(b)** Sampling visits by month (vertical bars) compared to seasonal and interannual rainfall variability (1st y-axis); systematic sampling of 8 landscapes over 5 years spanned elevations (2nd y-axis) in northern Peru, 2015–2019. Blue bars/lines represent wetter and yellow drier years.

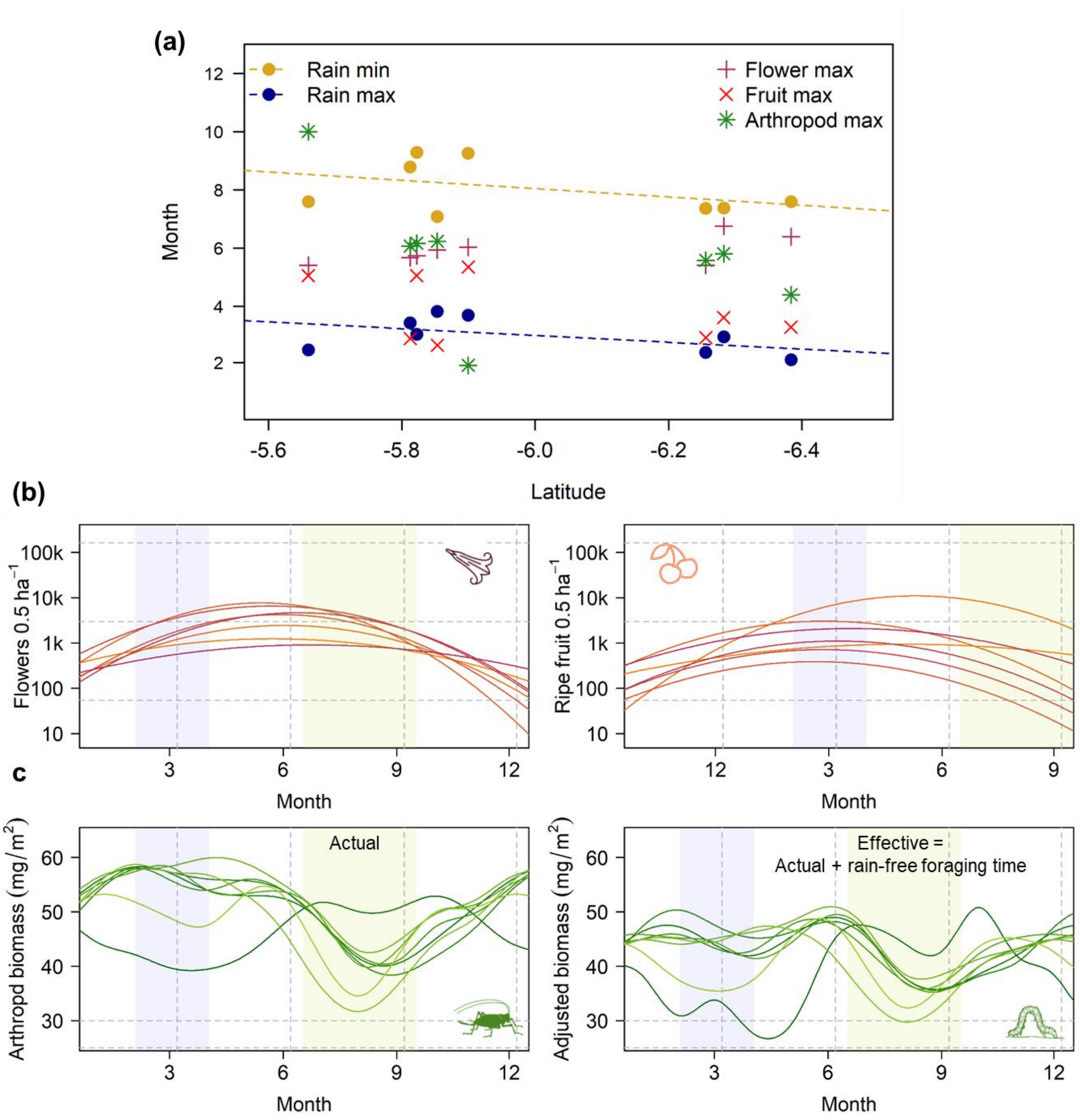

Figure S3. Timing of seasonal resources across climatic gradients

(a) Timing of seasonal resource peaks relative to rainfall by latitude; rainfall timing varied <2 weeks across the system (Newell et al. 2022). **(b)** Seasonal variation in resource availability for bird-pollinated flowers and bird-dispersed fruit (calendar month on the *x*-axis shifted to center on peaks). **(c)** Actual arthropod biomass compared to effective arthropod biomass adjusted for rain-free foraging time. Shaded areas represent wet (blue) and dry seasons (tan).

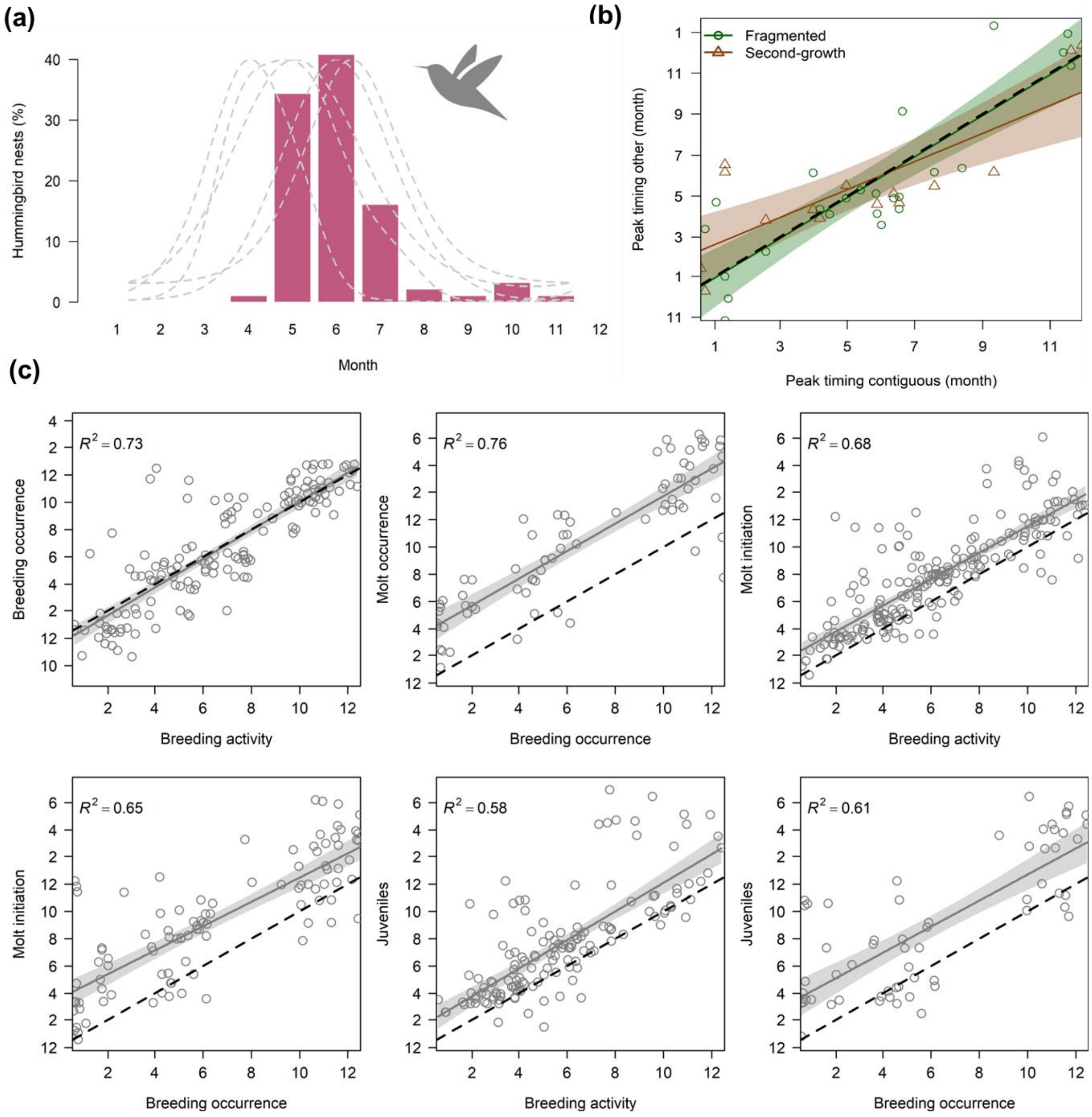

Figure S4. Avian phenology model validation

(a) Reproductive timing by trophic guild in contiguous forest compared to other habitats. Habitat-level models pooled across species by diet based on monthly sampling effort. Black-dashed line represents one-to-one line with shaded polygons showing 95% CI for each habitat type. **(b)** Percentage of hummingbird nests (Trochilidae) initiated by month compared to species models (dashed lines). Nests (N = 93) were found during roadside transects May–July 2017, and opportunistically during mist-netting. **(c)** Comparison of modeling approaches to describe avian reproductive phenology. Peak timing (month) modeled for counts of combined breeding activity (brood patch, cloacal protuberance, backdated juveniles, molt) compared to phenophase occurrence models for a single observation type. Dashed 1-to-1 line shown in black.

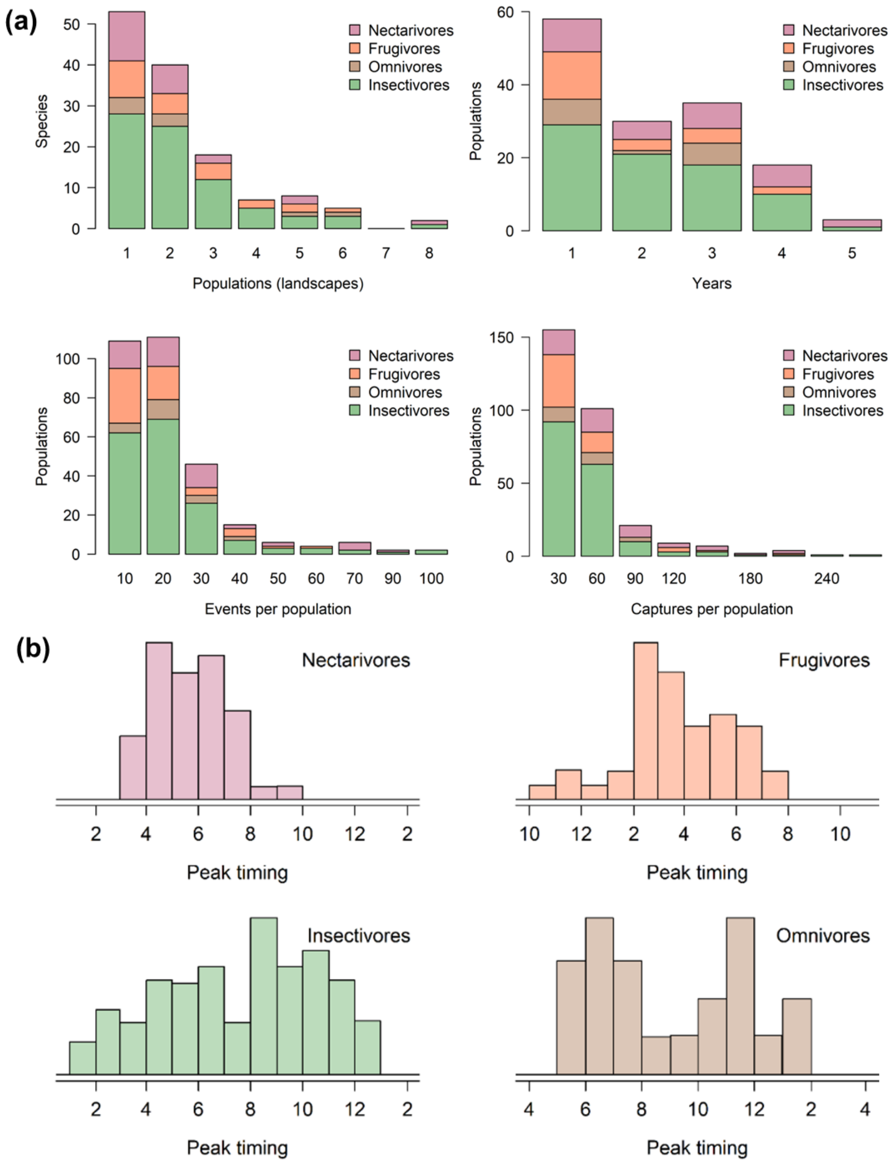

Figure S5. Sample sizes and regional distribution of peaks by trophic guild

(a) Across the same ecoregion (Yungas), bird species occurred at multiple landscapes with sufficient data to model reproductive phenology for different populations of the same species. In tropical systems most species are rare, and sampling effort (reproductive events/captures) per population were right skewed. To examine a broad range of phylogenetic diversity in montane cloud forest, phenology for each population was modeled separately contributing equally to the analysis independent of sampling effort. **(b)** The distribution of peak timing throughout the year differed among trophic guilds. For nectarivores and frugivores nesting peaks by species primarily spanned a restricted 6–8-month period centered on the transition to the dry season (nectarivores) or wet season (frugivores). At regional scales, insectivores and omnivores appeared to nest throughout the year, although few species nested during the wet season.

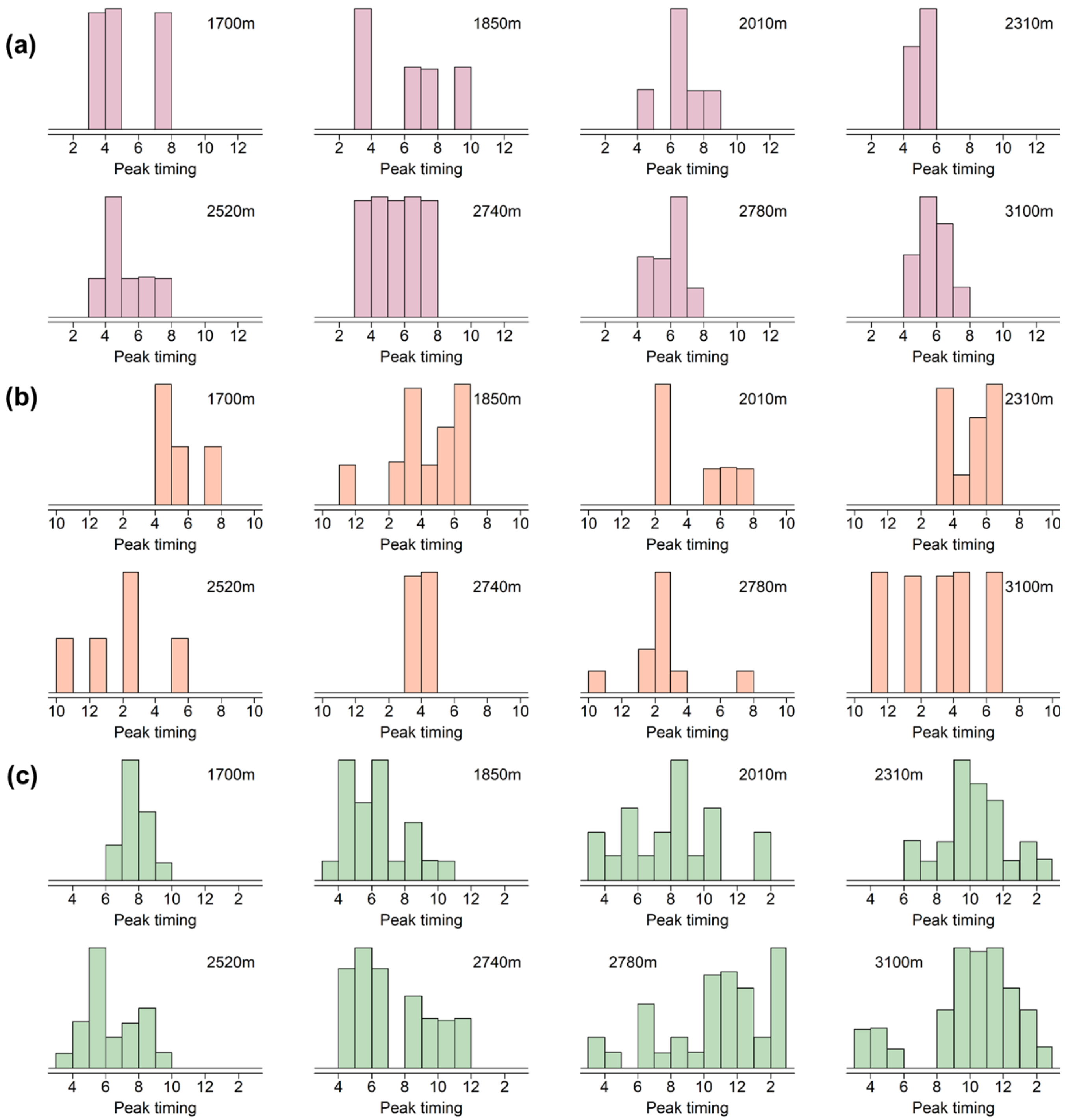

Figure S6. Local distribution of bird species peaks by trophic guild

Local distribution of bird species peaks throughout the year by trophic guild for **(a)** nectarivores, **(b)** frugivores, and **(c)** insectivores/omnivores which primarily nested during restricted breeding seasons at local scales.

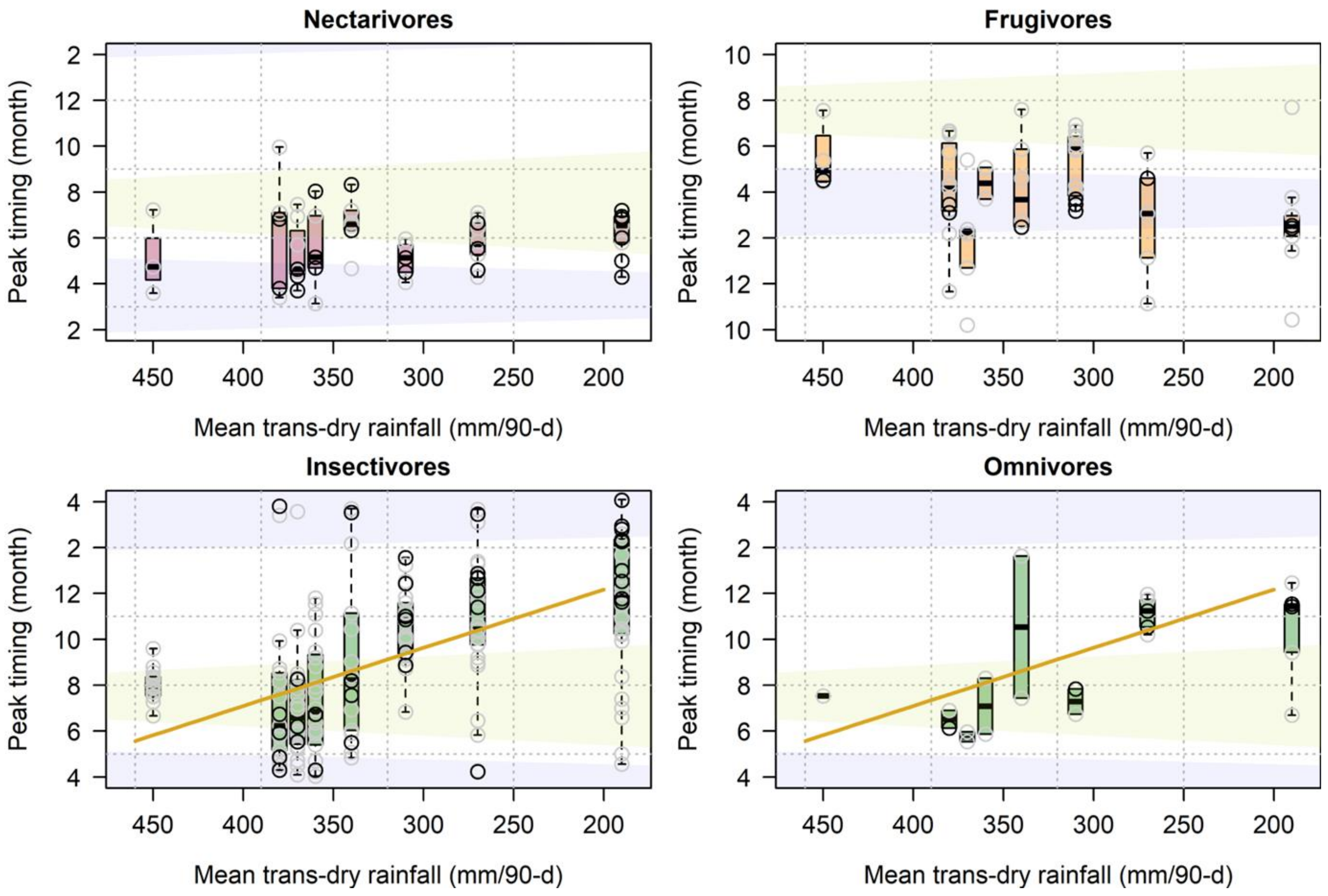

Figure S7. Modular response of cloud forest birds to seasonal rainfall

Cloud forest bird communities showed a modular response to seasonal rainfall, in which spatial variation in phenology differed among trophic guilds. Both insectivores and omnivores showing the same spatial shift in peak timing in response to changes in transition dry season rainfall (Apr–Jun). Shaded areas represent wet (blue) and dry seasons (tan).

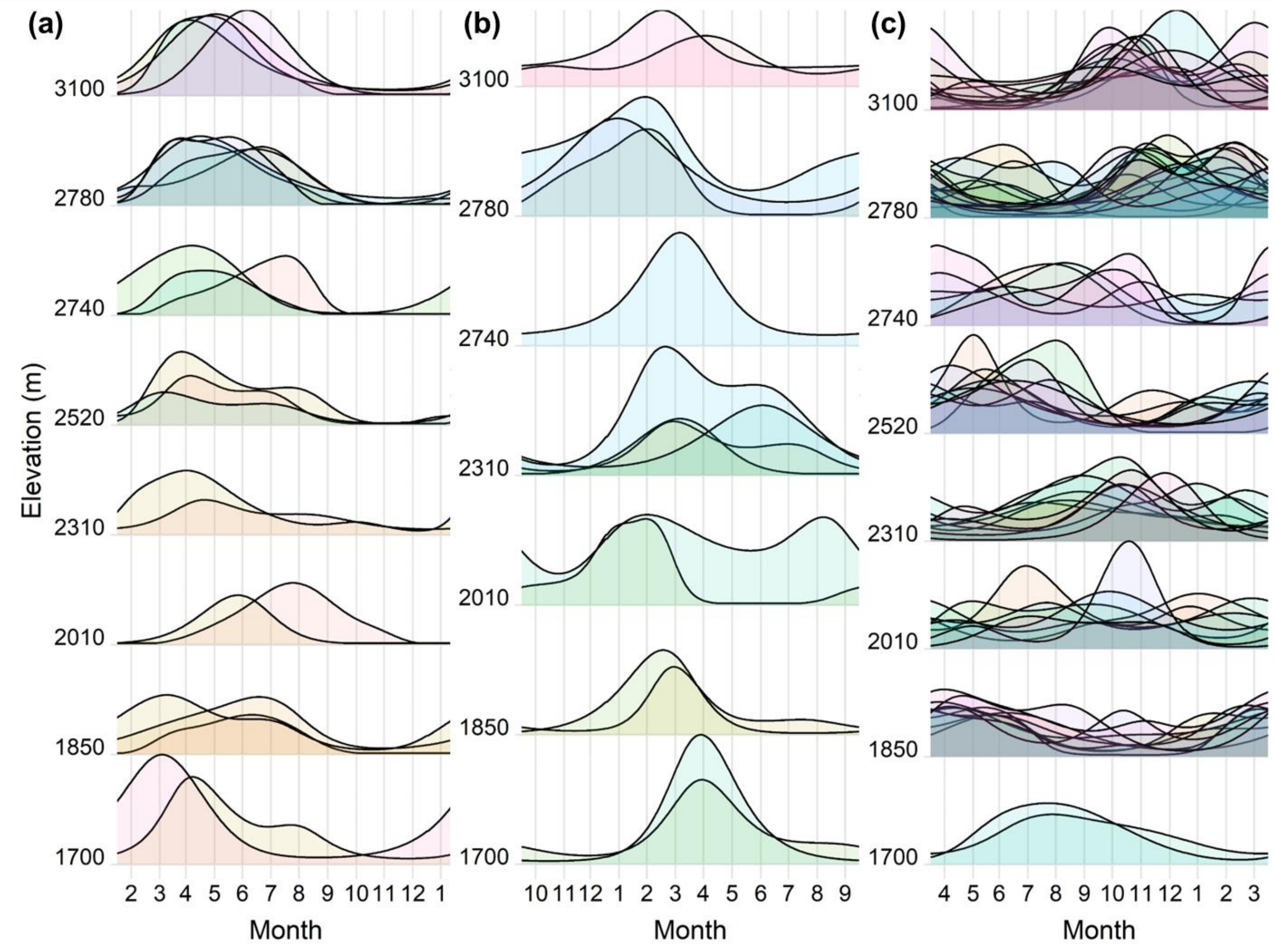

Figure S8. Annual cycles by trophic guild across elevations

Comparison of annual cycles for **(a)** nectarivores, **(b)** frugivores, and **(c)** insectivores across a 1700–3100 m elevation and rainfall gradient at 5–7 °S in the Andes of northern Perú. At low latitude with limited seasonal differences in temperature (<2°C) or day length (45 min), there was no support for effects of elevation on avian phenology and AIC_c models including elevation were equivalent or worse than a null model (Tables S12–14). Calendar year shifted on the *x*-axis to center temporal clustering among different trophic guilds.

SI Tables

Table S1. Correlation matrix for climatic and resource variables

Pearson correlation matrix of geographic, climatic, and resource variables included in final AIC_c model selection analysis and confirmatory path analysis. Ranges indicates months included in seasonal metrics. Correlations ≥ 0.70 highlighted in bold.

	Lat	Long	Elev	RainYr	Rain3	Rain6	Rain9	Rain12	Cld9	FlwrT	FruitT	Bug6
InterAndean (7 landscapes)												
Long	-0.36	-	-	-	-	-	-	-	-	-	-	-
Elev	-0.53	0.17	-	-	-	-	-	-	-	-	-	-
Rain 1-12	0.20	0.42	-0.10	-	-	-	-	-	-	-	-	-
Rain 1-3	-0.34	0.48	0.23	**0.80**	-	-	-	-	-	-	-	-
Rain 4-6	**0.89**	-0.03	-0.51	0.62	0.08	-	-	-	-	-	-	-
Rain 7-9	0.51	0.52	-0.17	0.65	0.21	**0.75**	-	-	-	-	-	-
Rain 10-12	-0.29	0.62	0.55	0.57	**0.74**	0.00	0.23	-	-	-	-	-
Cloud 7-9	0.27	**-0.76**	-0.17	-0.39	-0.61	0.01	-0.28	-0.67	-	-	-	-
FlwrTiming	-0.48	-0.29	0.61	-0.02	0.44	-0.48	-0.63	0.40	-0.08	-	-	-
FruitTiming	0.29	-0.54	-0.66	0.15	0.02	0.27	-0.32	-0.31	0.42	0.09	-	-
Bug 4-6	0.62	-0.24	-0.25	-0.21	**-0.71**	0.43	0.43	-0.53	0.62	**-0.70**	-0.10	-
Bug 7-9	**0.78**	0.10	-0.41	0.45	-0.14	**0.86**	**0.88**	-0.17	0.15	**-0.74**	-0.03	**0.74**
InterAndean to Eastern slope (8 landscapes)												
Long	-0.16	-	-	-	-	-	-	-	-	-	-	-
Elev	-0.66	-0.02	-	-	-	-	-	-	-	-	-	-
Rain 1-12	0.52	0.45	-0.51	-	-	-	-	-	-	-	-	-
Rain 1-3	0.33	0.44	-0.41	**0.95**	-	-	-	-	-	-	-	-
Rain 4-6	**0.87**	0.20	**-0.70**	**0.87**	**0.73**	-	-	-	-	-	-	-
Rain 7-9	0.62	0.42	-0.57	**0.91**	**0.88**	**0.90**	-	-	-	-	-	-
Rain 10-12	0.41	0.43	-0.40	**0.90**	**0.96**	**0.76**	**0.92**	-	-	-	-	-
Cloud 7-9	0.29	-0.69	-0.21	-0.10	-0.15	0.11	0.03	-0.07	-	-	-	-
FlwrTiming	-0.59	-0.37	**0.70**	-0.38	-0.22	-0.61	-0.60	-0.31	-0.13	-	-	-
FruitTiming	0.45	-0.35	**-0.74**	0.44	0.40	0.49	0.31	0.34	0.43	-0.12	-	-
Bug 4-6	-0.19	-0.35	0.39	**-0.80**	**-0.94**	-0.57	**-0.76**	**-0.91**	0.18	0.08	-0.42	-
Bug 7-9	**0.84**	0.25	-0.63	**0.75**	0.56	**0.92**	**0.85**	0.61	0.20	**-0.79**	0.27	-0.33

Table S2. Summary of bird sample sizes by landscape and year.

Our analysis used a spatial population-level replicate for a range of avian phylogenetic diversity across an elevation and rainfall gradient at 5–6°S in the Andes of northern Perú, 2015–2019. To limit dominance by a few common species, each population was modeled separately (species-by-landscape) and weighted equally independent of the input sampling effort (events/captures). Our analysis of 133 bird species included intra-specific variation in reproductive phenology for different populations of the same species across the climatic gradient. For analysis we used a nested mixed-effects model with a crossed random intercept for phylogenetic structure and space: (1|family/genus/species) + (1|landscape). We also ran spatiotemporal annual models (species-by-landscape-by-year) for ~50% of populations with sufficient sample sizes by year.

Phylogeny[1]	Bird community (28, 94, 133)			Nectarivores (2, 16, 24)			Frugivores (7, 15, 23)			Insectivores/omnivores (14/5, 55/8, 77/9)		
Group	*N*[2]	Events[3]	Caps.	*N*	Events	Caps.	*N*	Events	Caps.	*N*	Events	Caps.
Spatial: Elevation (m)												
1700[4]	21	169	294	3	31	52	4	42	76	14	96	166
1850	39	577	1330	5	99	275	11	119	367	23	359	688
2010[5]	32	323	665	6	54	163	6	67	150	20	202	352
2310	41	637	1471	5	118	308	12	144	348	24	375	815
2520	34	388	787	7	86	258	5	21	35	22	281	494
2740[5]	29	299	569	5	75	185	2	15	20	22	209	364
2780	56	885	1903	9	229	500	10	89	160	37	567	1243
3100	49	571	1206	10	185	394	5	38	79	34	348	733
Total	301	3849	8225	50	877	2135	55	535	1235	196	2437	4855
Temporal: Year												
2015	44	343	729	10	85	153	4	30	53	30	228	523
2016	59	407	1058	14	125	262	6	44	152	39	238	644
2017	100	832	1575	23	249	479	16	95	241	61	488	855
2018	68	518	964	8	40	98	6	53	82	54	425	784
2019	39	301	694	19	131	401	7	48	98	13	122	195
Total	310	2401	5020	74	630	1393	39	270	626	197	1501	3001

[1] Number of families, genera, species in parentheses

[2] For the spatial analysis *N* = number of populations; for the temporal analysis *N* = number of annual models.

[3] Includes reproductive and related events (breeding activity, juveniles, molt scores).

[4] Landscape discontinued in 2016 due to safety concerns.

[5] Landscape added in 2016.

Table S3. Input filters by sample size used in analysis

Filter0: all input models [N_{F0} = 301]
Filter1: ≥3 reproductive events, ≥6 individuals, ≥3-month span [N_{F1} = 251]
Filter2: ≥10 reproductive events, ≥10 individuals, ≥5-month span [N_{F2} = 120]
Filter3: ≥10 reproductive events, ≥30 individuals, ≥6-month span [N_{F3} = 74]

Table S4. Parameter estimates by sample size for peak timing shifts by trophic guild

Interactions between diet and seasonal rainfall explained phenological shifts across the full cloud forest bird community. Result for climatic variables from AIC model selection by trophic guild compared to fit for the full interactive model (diet * transition dry rainfall). Parameter estimates and model fit for shifts in peak timing by trophic guild filtered for different input sample sizes. Models with poor fit shown in gray ($R^2 \leq 0.00$). Range indicates months for seasonal metrics.

		Nesting timing (month)							
		Wetter			Drier			R^2	
N_{full}	N_{diet}	Est.	LCI	UCI	Est.	LCI	UCI	Diet	$Full_{int}$
		Nectarivores ~ Cloud 7-9 (dry season)							
301	50	6.3	3.9	8.7	6.4	4.6	8.2	-0.02	0.22
251	46	6.4	3.8	9.1	6.5	4.6	8.4	-0.02	0.25
120	27	4.9	1.9	7.9	6.1	4.2	7.9	-0.03	0.32
74	21	4.9	1.2	8.6	6.2	3.9	8.6	-0.04	0.43
168[1]	24[1]	6.0	4.7	7.2	4.3	3.1	5.5	0.08	0.04
		Frugivores ~ Rain 5-8 (dry season)							
301	55	4.7	3.5	6.0	1.5	0.4	2.6	0.17	0.22
251	40	4.3	3.2	5.5	0.8	-0.3	1.9	0.29	0.25
120	16	3.5	2.5	4.5	1.4	0.3	2.4	0.30	0.32
74	10	2.4	1.6	3.3	2.4	1.6	3.3	0.00	0.43
168[1]	36[1]	4.1	2.8	5.3	1.0	-0.3	2.4	0.17	0.04
		Insectivores ~ Rain 4-6 (transition dry)							
301	196	5.4	4.0	6.7	10.0	8.9	11.1	0.18	0.22
251	165	5.1	3.7	6.6	10.1	9.0	11.2	0.19	0.25
120	77	4.8	2.8	6.8	11.6	10.0	13.2	0.20	0.32
74	43	5.6	3.9	7.3	13.0	11.3	14.7	0.41	0.43
168[1]	108[1]	5.3	3.7	6.9	12.1	10.4	13.8	0.23	0.04

[1]intraspecific shifts filtered for species spanning > 2 landscapes.

Table S5. Parameter estimates by sample size for bimodal breeding of insectivores

The probability of detecting bimodal breeding increased at sites with greater arthropod biomass during the transition to the dry season; there was weak support for effects of dry season cloud cover and wet season rainfall. Parameter estimates and model fit for phenology metrics filtered by different input sample sizes. Models with poor fit shown in gray ($R^2 \leq 0.00$). Range indicates months for seasonal metrics.

	Probability bimodal						
		Wetter			Cloudier/Drier		
N_{diet}	Est.	LCI	UCI	Est.	LCI	UCI	R^2
Arthropods 4-6 (transition dry)							
196	0.06	0.00	0.13	0.28	0.14	0.42	0.01
165	0.07	-0.01	0.15	0.33	0.17	0.49	0.01
77	0.01	-0.01	0.03	0.72	0.49	0.95	0.11
43	0.00	0.00	0.00	1.00	1.00	1.00	-0.24
Cloud 6-9 (dry season)							
196	0.16	0.08	0.24	0.22	0.06	0.38	0.00
165	0.17	0.08	0.26	0.31	0.10	0.52	0.00
77	0.18	0.06	0.30	0.63	0.32	0.94	0.07
43	0.00	0.00	0.00	1.00	1.00	1.00	-0.05
Rain 10-3 (wet season)							
196	0.06	-0.01	0.13	0.24	0.13	0.34	0.01
165	0.06	-0.01	0.14	0.29	0.16	0.41	0.01
77	0.02	-0.03	0.08	0.50	0.29	0.71	0.05
43	0.04	-0.02	0.10	0.57	0.20	0.94	0.10

Table S6. Parameter estimates by sample size for interannual variability of trophic guilds. Interannual variability for ~50% of populations modeled by year included weak effects of rainfall and resources on insectivores and frugivores, but no difference among years for nectarivores. Although there was no evidence for changes in timing frugivores increased reproductive effort with greater wet season rainfall accumulation (Supplementary Fig. 9b). Insectivores shifted nesting away from the dry season related to reduced arthropod biomass in drier years (Newell et al. 2023) while reproductive effort was reduced for populations that nested after the dry season; effort represents change in events per 100 individuals. Models with poor fit shown in gray ($R^2 \leq 0.00$).

				Normal		Extreme (dry/wet)			
Model	Subset	*N*, Pops.	Est.	LCI	UCI	β	LCI	UCI	R^2
			Frugivores						
			Reproductive effort (events)						
Rainfall past 90-days	Full	22, 11	18.2	11.6	28.5	2.8	0.7	7.3	-0.23
	Same[1]	15, 10	8.9	5.8	13.6	4.3	2.1	8.2	0.35
			Insectivores[2]						
			Timing shift (months)						
Rainfall past 180-days	Before dry	35, 15	-0.9	-1.8	-0.1	2.6	0.9	4.3	0.18
	After dry	62, 27	-1.2	-2.4	0.0	1.9	-0.1	4.0	0.04
Arthropods past 30-days	Full	97, 42	0.0	-0.3	0.3	2.1	0.8	3.3	0.09
			Reproductive effort (events)						
Rainfall past 150-days	Before dry	35, 15	32.7	27.4	39.0	-0.01	-0.31	0.41	-0.03
	After dry	62, 27	47.7	33.6	67.7	-0.42	-0.66	-0.01	0.10
Arthropods past 90-days	Full	97, 42	38.0	30.7	47.0	-0.38	-0.53	-0.19	0.05

[1] Parameter estimates for years with the same timing of sampling in Jun/Jul–Nov, 2015–2017.
[2] Omnivores were not included because models fit the data poorly.

Table S7. Parameter estimates by sample size for confirmatory path analysis

Parameter estimates to relate reproductive phenology of avian trophic guilds to resources and climate across an elevation and rainfall gradient in northern Peru, 2015–2019. Variables selected based on support in other analyses. Models fitted using piecewiseSEM (Lefcheck 2016) with crossed random intercepts (1|family/genus/species) + (1|landscape). Landscapes weighted equally and links selected based on d-separation tests of model fit (Shipley 2013) with coefficients standardized by range. Range indicates months for seasonal metrics.

Response	Predictor	β	SE	*df*	T_{crit}	*P*-val	βstd	R^2 Marg	R^2 Cond
				Nectarivores shift (N_{F0} = 50)					
Nesting timing	Flower timing	0.39	0.18	15	3.90	0.07	0.19	0.08	0.65
	Cloud 6-8	0.42	0.14	12	7.29	0.02	0.21		
Flower timing	Rain 6-8	-0.84	0.04	4	-20.19	0.00	-0.85	0.74	1.00
	Longitude	-0.25	0.03	4	-9.30	0.00	-0.29		
	Latitude	0.08	0.03	4	2.63	0.06	0.07		
Rain 6-8	Cloud 6-8	-0.40	0.09	3	-4.30	0.02	-0.39		
	Latitude	0.39	0.10	3	3.88	0.03	0.32	0.18	1.00
	Longitude	-0.09	0.08	3	-1.04	0.38	-0.10		
	Elevation	-0.52	0.16	3	-3.23	0.05	-0.47		
Cloud 6-8	Latitude	0.04	0.14	4	0.29	0.79	0.03	0.19	1.00
	Longitude	-0.42	0.12	4	-3.49	0.03	-0.49		
	Elevation	-0.16	0.21	4	-0.78	0.48	-0.15		
				Frugivores shift (N_{F0} = 55)					
Nesting timing	Fruit timing	0.15	0.30	2	0.16	0.72	0.05	0.16	0.48
	Rain 6-8	0.89	0.31	3	5.89	0.08	0.32		
	Cloud 6-8	0.21	0.29	4	0.38	0.57	0.07		
Fruit timing	Rain 6-8	0.47	0.06	2	8.51	0.01	0.56	0.36	1.00
	Cloud 6-8	-0.27	0.03	2	-8.67	0.01	-0.31		
	Latitude	-0.52	0.09	2	-6.06	0.03	-0.51		
	Longitude	-1.28	0.05	2	-27.24	0.00	-1.71		
	Elevation	-0.07	0.04	2	-1.70	0.23	-0.07		
Rain 6-8	Cloud 6-8	0.27	0.07	3	4.03	0.03	0.26	0.85	1.00
	Latitude	0.51	0.13	3	4.08	0.03	0.42		
	Longitude	0.64	0.07	3	8.64	0.00	0.72		
	Elevation	-0.31	0.08	3	-3.94	0.03	-0.28		
Cloud 6-8	Latitude	-0.16	0.31	4	-0.50	0.64	-0.13	0.25	1.00
	Longitude	-0.52	0.14	4	-3.63	0.02	-0.60		
	Elevation	0.38	0.17	4	2.22	0.09	0.35		
				Insectivores/omnivores shift (N_{F0} = 196)					
Nesting timing	Arthropods 7-9	-1.19	0.47	5	6.80	0.05	-0.36	0.25	0.54
Arthropods 7-9	Rain 4-6	-0.54	0.03	3	-17.62	0.00	-0.55	0.22	1.00
	Latitude	0.44	0.03	3	13.23	0.00	0.34		
	Longitude	-0.06	0.09	3	-0.68	0.55	-0.06		
	Elevation	-1.57	0.11	3	-14.75	0.00	-1.30		
Rain 4-6	Latitude	0.60	0.07	4	9.07	0.00	0.46	0.21	1.00
	Longitude	0.03	0.18	4	0.14	0.89	0.03		
	Elevation	1.01	0.17	4	5.97	0.00	0.83		
				Insectivores/omnivores bimodal (N_{F2} = 77)					
Bimodal nesting	Arthropods 4-6	1.43	0.52	77	2.78	0.01	0.50	0.23	0.35
Arthropods 4-6	Rain 6-8	-0.41	0.06	4	-6.92	0.00	-0.31	0.47	1.00
	Rain 1-3	-0.30	0.04	4	-8.45	0.00	-0.23		
	Rain 6-8 * 1-3	-0.45	0.06	4	-7.05	0.00	-0.51		
Rain 6-8	Rain 1-3	-0.08	0.02	3	-4.07	0.03	-0.08	0.54	1.00
	Latitude	0.94	0.04	3	25.55	0.00	0.78		
	Longitude	1.91	0.21	3	8.96	0.00	2.14		
	Elevation	0.23	0.07	3	3.07	0.05	0.20		
Rain 1-3	Latitude	-1.34	0.16	4	-8.45	0.00	-1.09	0.38	1.00
	Longitude	-5.86	1.08	4	-5.43	0.01	-6.47		
	Elevation	2.62	0.35	4	7.60	0.00	2.31		

SI Extended Figures

Modeling seasonal resources

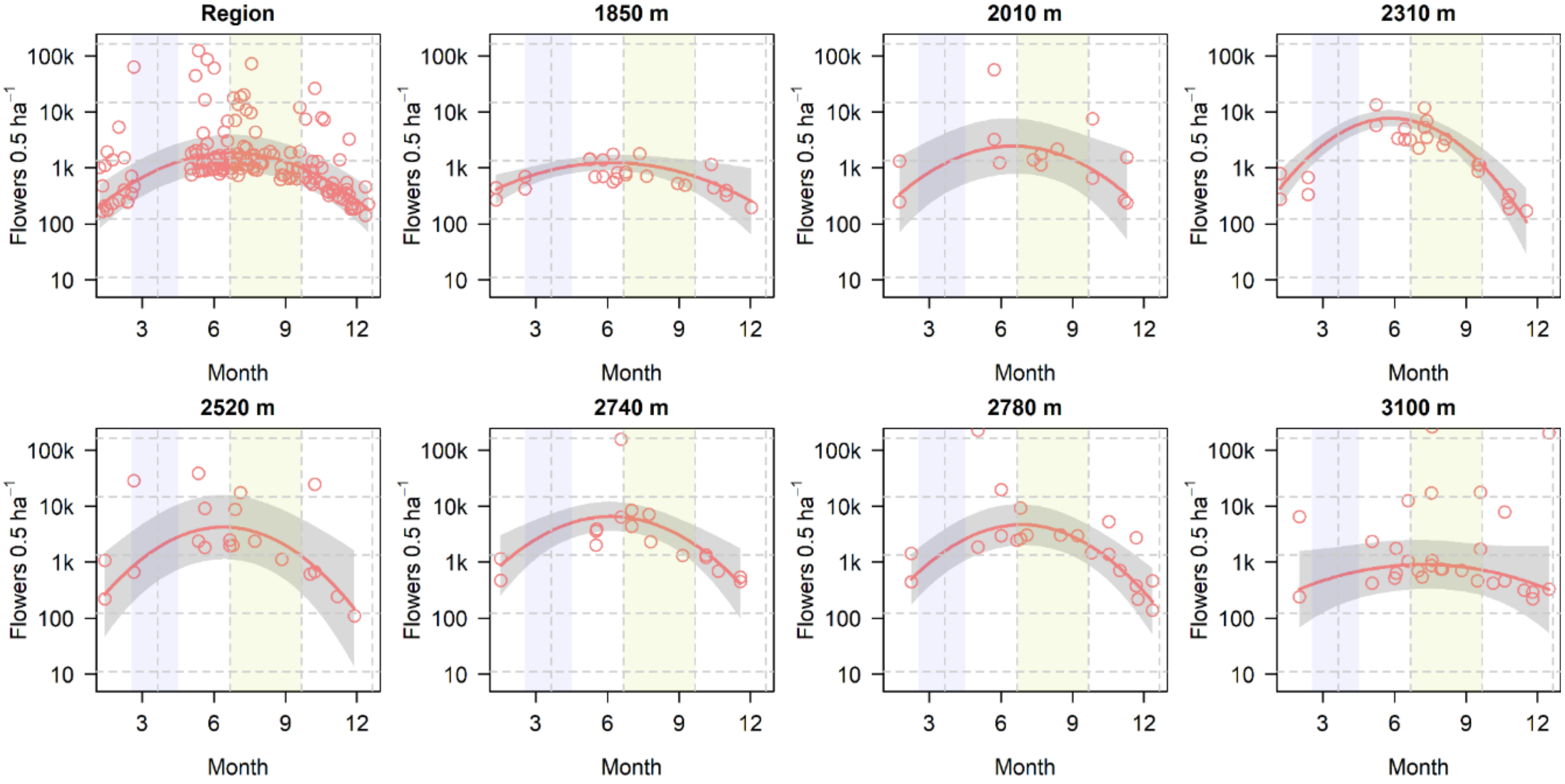

Figure S9. Seasonal abundance of bird-pollinated flowers across elevation

Seasonal abundance of bird-pollinated flowers across an elevation and rainfall gradient. Plant phenology transects across 7 landscapes in northern Peru, 2016–2019. Quadratic mixed-models integrate individual-based and area transects weighted by sampling effort. Shaded areas represent wet (blue) and dry seasons (tan).

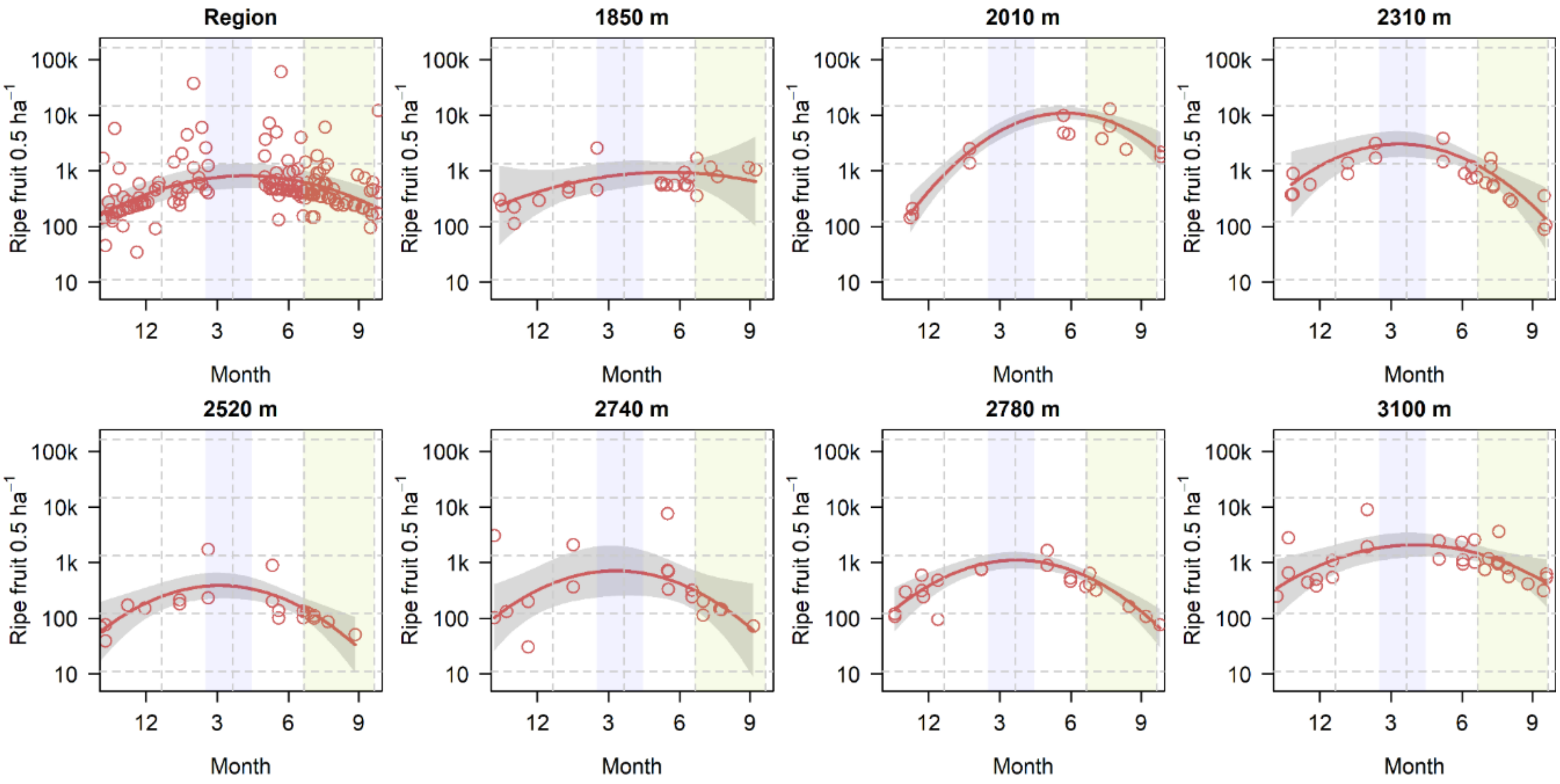

Figure S10. Seasonal abundance of bird-dispersed fruit across elevation

Seasonal abundance of ripe understory fruit across an elevation and rainfall gradient. Plant phenology transects across 7 landscapes in northern Peru, 2016–2019. Quadratic mixed-models integrate individual-based and area transects weighted by sampling effort. Shaded areas represent wet (blue) and dry seasons (tan).

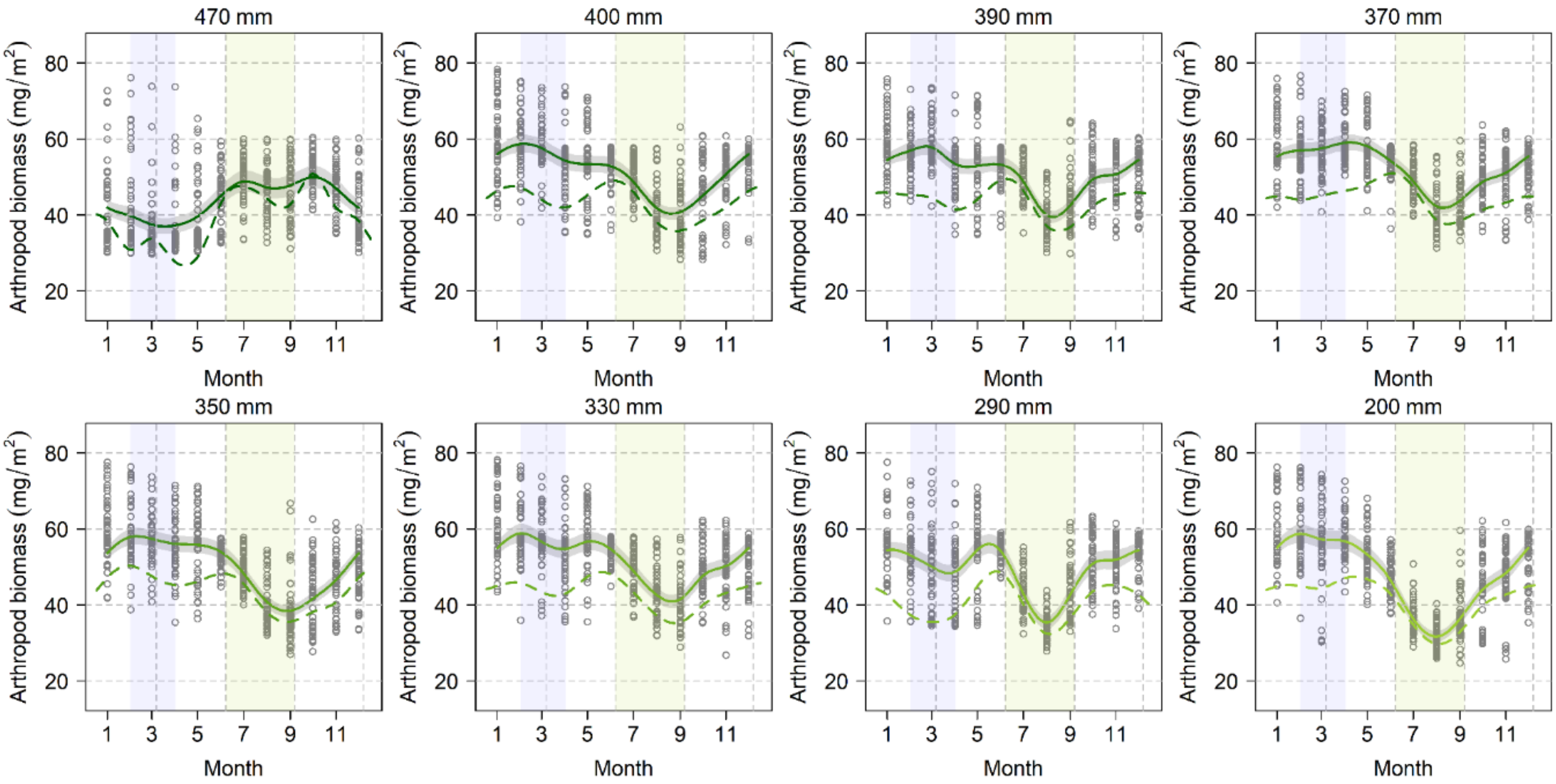

Figure S11. Seasonal arthropod biomass by transition dry season rainfall

Model of long-term seasonal changes in biomass of foliage arthropods based on intermediate rainfall. Model integrates in situ rain gauges and 50 years of regional rainfall (Newell et al. 2022) with dynamic response of arthropods to rainfall extremes (Newell et al. 2023b). Adjusted biomass (dashed lines) represents arthropod biomass multiplied by the proportion of rain-free daylight hours available for foraging (< 0.1 mm rain hr^{-1}). Landscapes ordered by transition dry-season rainfall (Apr–Jun). Shaded areas represent wet (blue) and dry seasons (tan). Figure modified from (Newell et al. 2023).

Bird species summaries by taxa and trophic guild

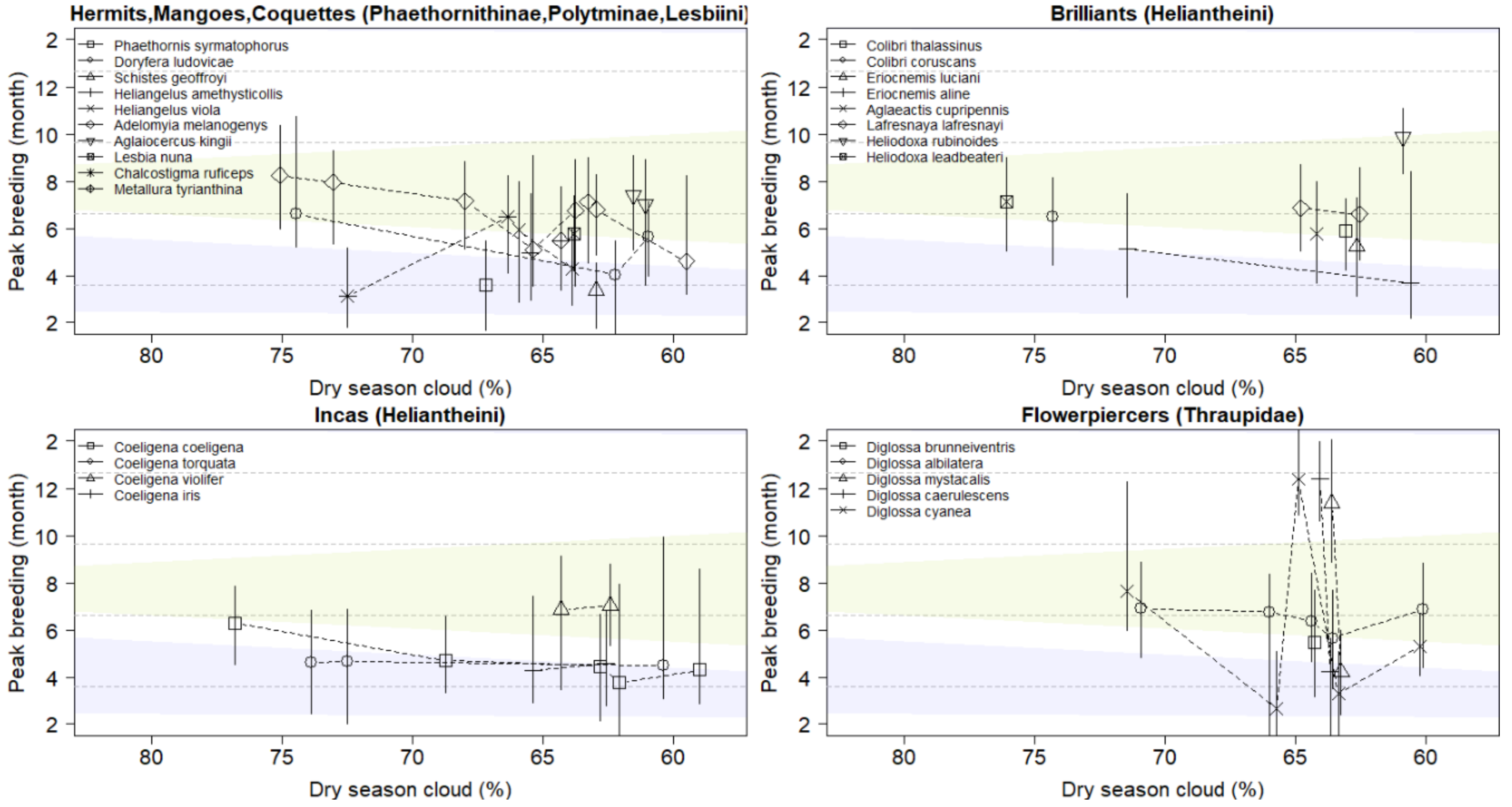

Figure S12. Spatial variation in reproductive timing of nectivorous species

Shifts in peak timing by species for nectarivores and related species. Hummingbirds (Trochilidae) grouped by subfamily while flowerpiercers (*Diglossa*) also include species with more insectivorous diets. Dashed lines connect the same species across landscapes and error bars represent peak length relative to wet (blue) and dry seasons (tan).

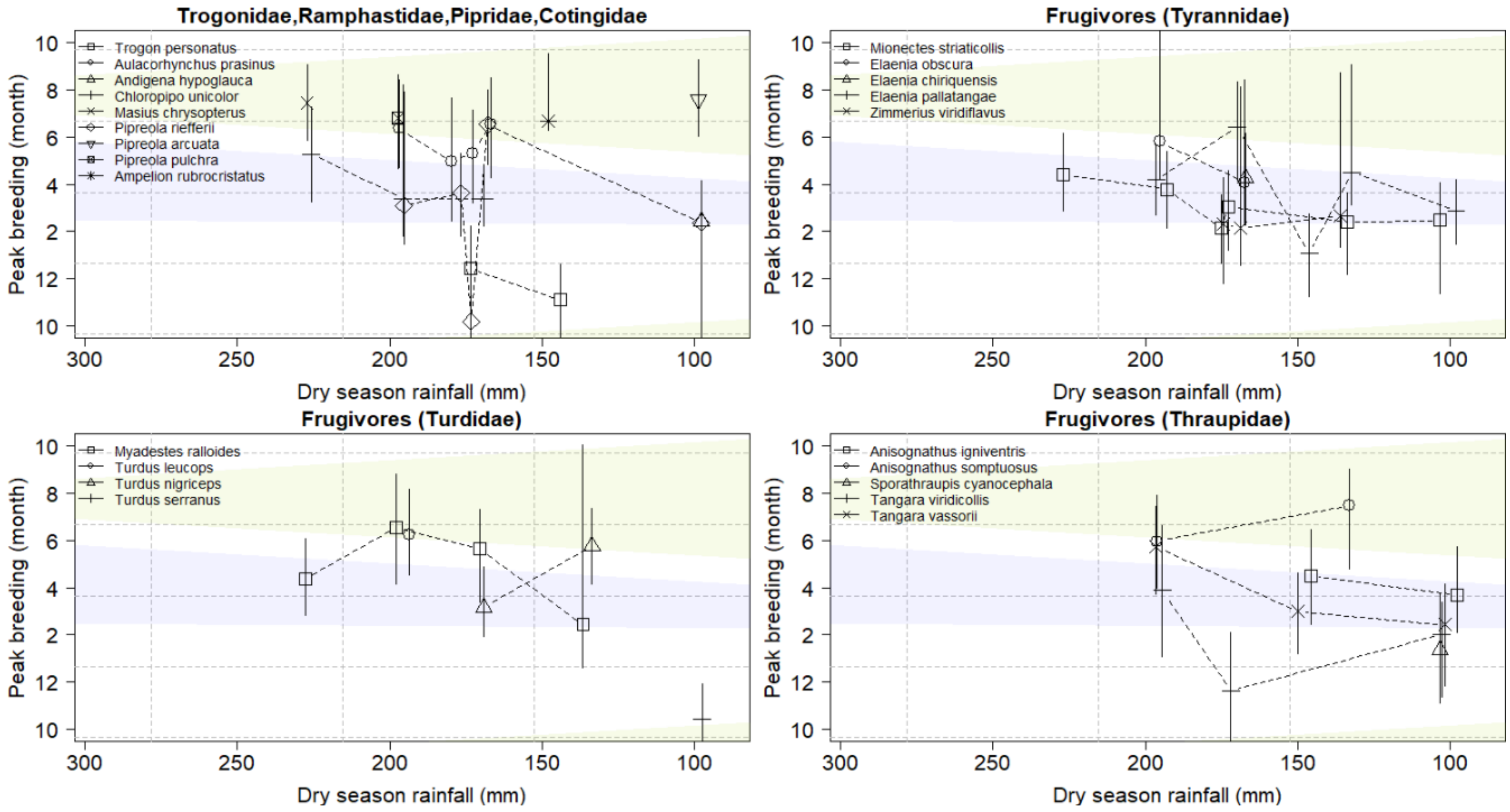

Figure S13. Spatial variation in reproductive timing of frugivorous species

Shifts in peak timing by species for frugivores. Dashed lines connect the same species across landscapes and error bars represent peak length relative to wet (blue) and dry seasons (ta

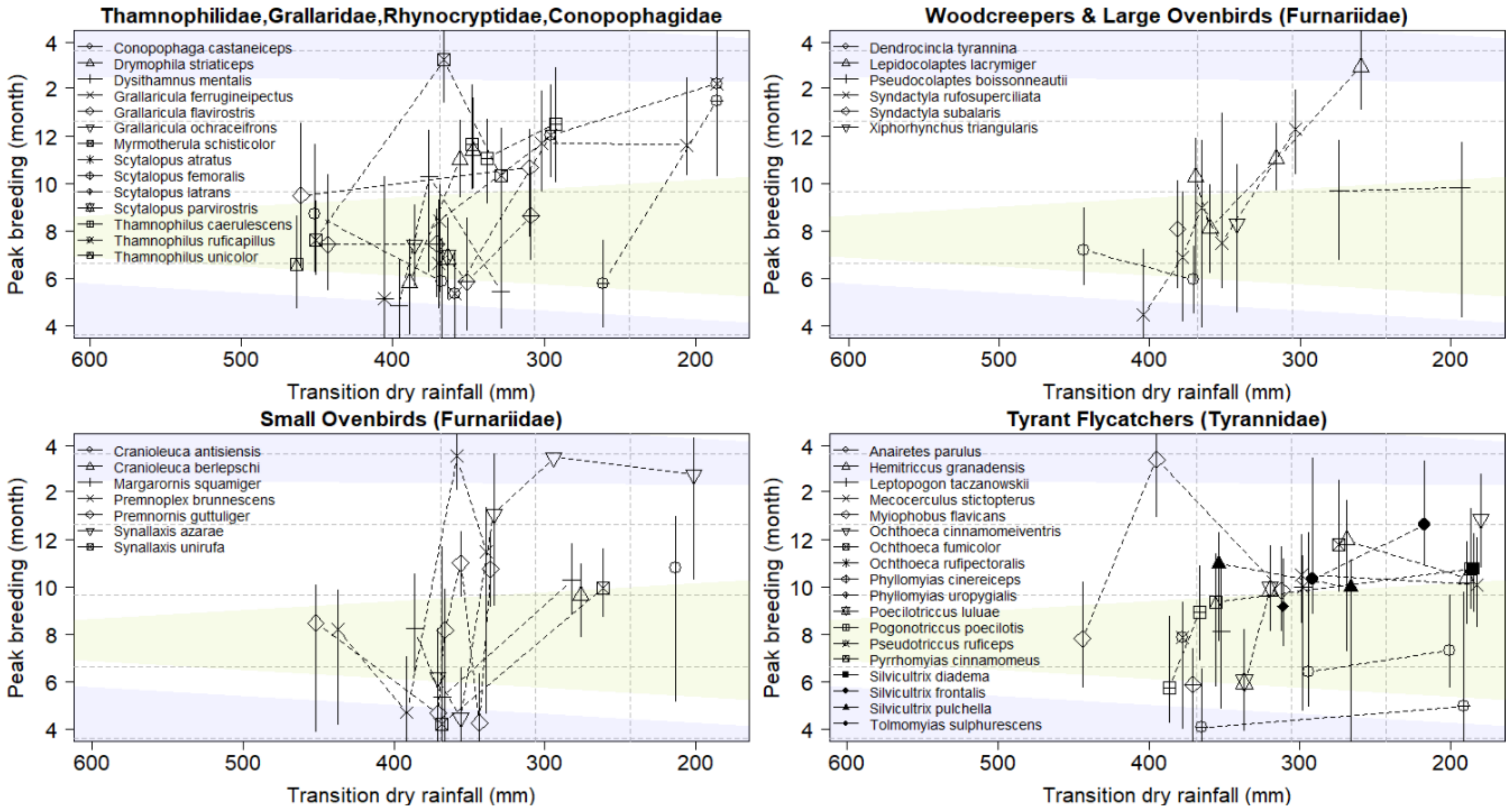

Figure S14. Spatial variation in reproductive timing of insectivorous species (suboscine)

Shifts in peak timing by species for suboscine insectivores. Dashed lines connect the same species across landscapes and error bars represent peak length relative to wet (blue) and dry seasons (tan)

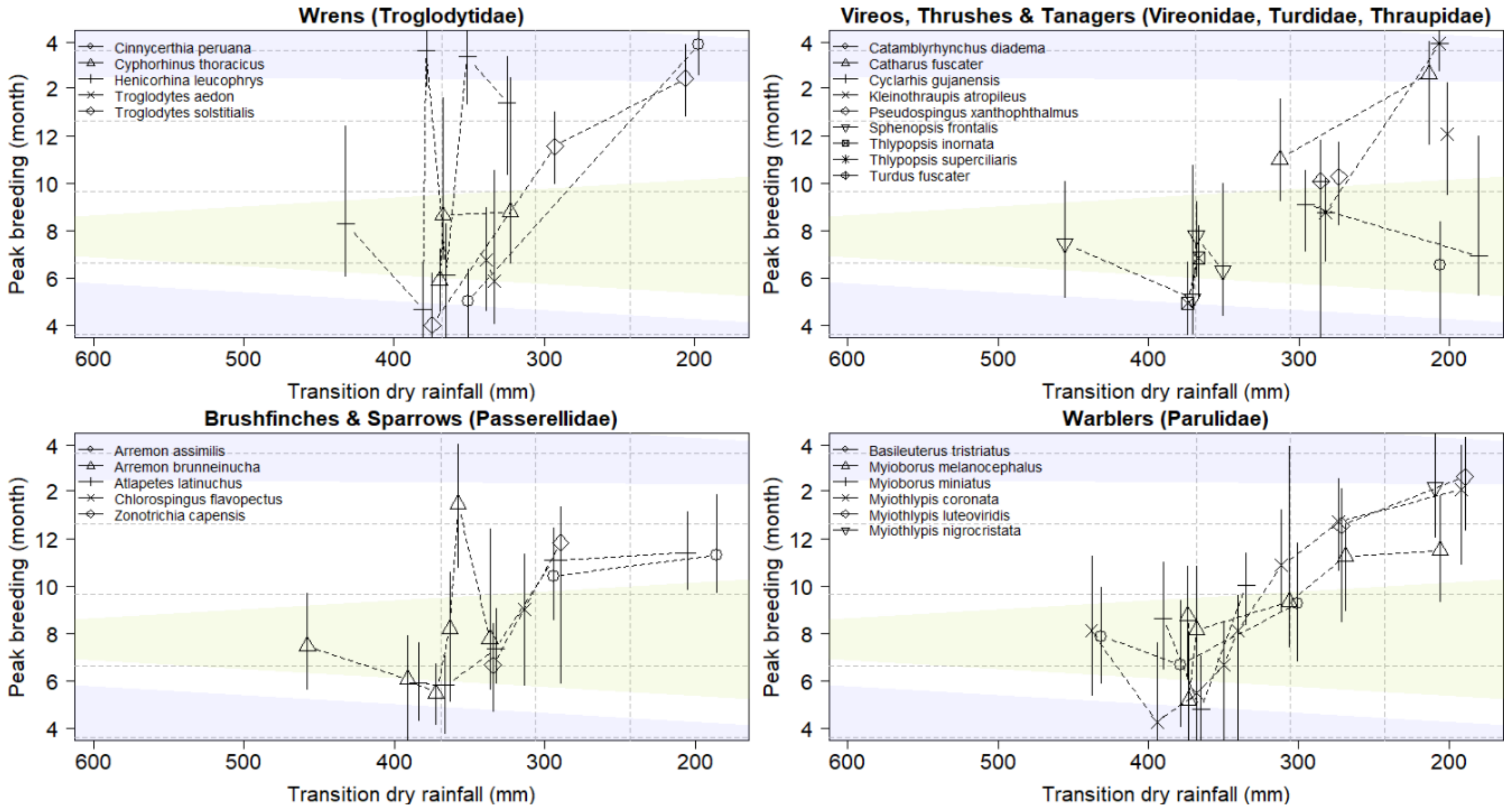

Figure S15. Spatial variation in reproductive timing of insectivorous and omnivorous species (oscine)

Shifts in peak timing by species for oscine insectivores and omnivores. Dashed lines connect the same species across landscapes and error bars represent peak length relative to wet (blue) and dry seasons (tan).

SI Extended Tables

Taxonomic scope

Table S8. Genera of bird-pollinated flowers and bird-dispersed fruit

Andean flowering and fruiting species detected on point-transects by family and genera. Plant phenology was monitored at 562 points on 51 visits across elevation and rainfall gradients in northern Peru, 2016–2019. Preliminary number of species identified using >10 000 photographs. Trees not included in analysis of understory fruit (T).

				Occurrence by elevation (m)						
Family	Genus	#Sp	Bird[1]	1850	2010	2310	2520	2740	2780	3100
Piperaceae	*Piper*	9	D	1	1	1	1	1	1	1
Siparunaceae	*Siparuna*	5	D	1	1	1	1	1	1	1
Lauraceae	*Lauraceae*^{T}	15	D	1			1	1	1	1
Chloranthaceae	*Hedyosmum*	2	D		1	1	1	1	1	1
Araceae	*Anthurium*	6	PD	1	1	1	1	1	1	
Alstroemeriaceae	*Bomarea*	8	PD	1		1	1	1	1	1
Smilacaceae	*Smilax*	2	D		1	1		1		1
Orchidaceae	*Eleanthus*	1	P	1			1	1		
	Fernandezia	1+	P					1	1	
Arecaceae	*Ceroxylon*^{T}	3	D			1			1	
Zingiberaceae	*Renealmia*	1	PD		1					
Bromeliaceae	*Aechmea*	1	P	1			1			
	Guzmania	2	P	1			1	1		
	Pitcairnia	1	P	1		1	1			
	Tillandsia	5	P	1	1	1	1	1	1	1
Berberidaceae	*Berbera*	1	D							1
Proteaceae	*Oreocallis*	1	P						1	1
Grossulariaceae	*Ribes*	1+	P							1
Fabaceae	*Erythrina*	1	P	1	1	1				
	Lupinus	1+	P							1
Polygalaceae	*Monnina*	1+	P						1	1
Rosaceae	*Hesperomeles*	2	D						1	1
	Rubus	2	D		1		1		1	1
Moraceae	*Ficus*^{T}	4	D	1	1	1	1			
Urticaceae	*Urera*	1	D	1						
Cucurbitaceae	*Gurania*	2	PD	1		1				
Begoniaceae	*Begonia*	4	P					1		
Elaeocarpaceae	*Vallea*^{T}	1	D							1
Clusiaceae	*Clusia*	2	PD		1				1	1
Hypericaceae	*Vismia*	1	D			1	1			
Passifloraceae	*Passiflora*	5	PD		1		1			1
Salicaceae	*Neosprucea*^{T}	1	D	1						
Onagraceae	*Fuchsia*	7	PD	1	1	1	1	1	1	1
Myrtaceae	*Myrcianthes*^{T}	1	D		1	1				
Melastomataceae	*Axinaea*	3	PD				1	1	1	1
	Brachyotum	6	P					1		1
	Meriania	4	P	1			1			1
	Miconia	18	D	1	1	1	1	1	1	1
	Tibouchina	2	P		1					
Anacardiaceae	*Toxicodendron*^{T}	1	D	1						
Meliaceae	*Ruagea*^{T}	2	D					1	1	
Malvaceae	*Abutilon*	1	P		1					

[1] P = bird-pollinated flowers, D = bird-dispersed fruit, PD = both, only identified to genus (+).

Table S8. Continued

Family	Genus	#Sp	Bird[1]	Occurrence by elevation (m) 1850	2010	2310	2520	2740	2780	3100
Tropaeolaceae	*Tropaeolum*	2	P					1	1	
Loranthaceae	*Aetanthus*	1	PD					1		
	Tristerix	1	PD							1
Phytolaccaceae	*Phytolacca*	1	D		1					
Loasaceae	*Nasa*	2	P							1
Cornaceae	*Cornus*	1	D		1		1			1
Polemoniaceae	*Cantua*	1	P				1			
Primulaceae	*Myrsine*	3	D		1		1			1
Styracaceae	*Styrax*[T]	2	D		1					
Actinidiaceae	*Saurauia*[T]	1	D	1			1	1		
Ericaceae	*Bejaria*	1	P							1
	Cavendishia	1	PD							1
	Disterigma	1	P							1
	Gaultheria	4	PD							1
	Macleania	1	PD					1	1	
	Psammisia	1	PD	1	1	1	1	1		
	Thibaudia	1	PD		1					
Rubiaceae	*Rubiaceae spp.*	1	PD	1			1	1		
	Cinchona	1	P					1		
	Elaeagia	2	D			1		1		
	Faramea	3	PD		1	1	1	1		
	Hoffmania	3	PD	1	1	1	1			
	Holtonia[T]	1	D		1					
	Palicourea	12	PD	1	1	1	1	1	1	1
	Psychotria	6	D	1	1	1	1	1		
Gentianaceae	*Macrocarpaea*	1	P						1	1
Solanaceae	*Cestrum*	1	P							1
	Solanum	13	D	1		1	1	1	1	1
	Trianaea	1	P			1				
Gesneriaceae	*Drymonia*	3	P	1	1	1		1		
Plantaginaceae	*Stemodia*	1	P						1	
Acanthaceae	*Aphelandra*	2	P				1		1	
	Dicliptera	1	P		1					
Bignoniaceae	*Delastoma*	1	P					1		
	Pithecoctenium	1	P		1					
Verbenaceae	*Citharexylum*[T]	1	PD	1		1		1		
	Duranta	1	PD						1	1
Lamiaceae	*Clinopodium*	1	P							1
	Salvia	3	P			1			1	1
Campanulaceae	*Burmeistera*	1	P		1					
	Centropogon	11	P		1	1	1	1	1	1
	Siphocampylus	2	P				1			
Asteraceae	*Barnadesia*	1	P						1	1
	Mutisia	1	P	1		1				
Adoxaceae	*Viburnum*	4	D		1	1		1	1	1
Araliaceae	*Oreopanax*	2	D			1	1	1		
	Schefflera[T]	2	D				1	1	1	

[1] P = bird-pollinated flowers, D = bird-dispersed fruit, PD = both, only identified to genus (+).

Table S9. Genera of nectarivores and frugivores.

Number of bird species, captures, and events by family and genera for nectarivores and frugivores across elevations. Models for 105 populations of 47 species in the Andes of northern Peru, 2015–2019. Complete spreadsheets with phenology metrics by population, input sampling effort (reproductive events/captures) and graphs of phenology models for each species across elevations available on Figshare (Newell et al. 2026).

					Species models by elevation (m)							
Family	*Genus*	Spp	Caps	Events	1700	1850	2010	2310	2520	2740	2780	3100
					Nectarivores							
Trochilidae	*Phaethornis*	1	15	11	1							
	Doryfera	1	24	8				1	1			
	Schistes	1	11	4		1						
	Heliangelus	2	95	46							2	1
	Adelomyia	1	555	192	1	1	1	1	1	1	1	1
	Aglaiocercus	1	45	16		1			1			
	Lesbia	1	12	3							1	
	Chalcostigma	1	57	13						1	1	
	Metallura	1	168	64								1
	Eriocnemis	2	146	47					1	1		1
	Aglaeactis	1	16	8								1
	Coeligena	4	769	333	1	1	2	1	2	1	2	2
	Lafresnaya	1	54	29							1	1
	Heliodoxa	2	34	11		1	1					
Thraupidae	*Diglossa*	2	113	84				1	1	1	1	2
					Frugivores							
Trogonidae	*Trogon*	1	12	11					1			1
Ramphastidae	*Aulacorhynchus*	1	12	13		1			1			
	Andigena	1	5	5							1	
Pipridae	*Chloropipo*	1	195	65	1	1		1				
	Masius	1	7	3	1							
Cotingidae	*Pipreola*	2	97	77		1		1		1	2	
	Ampelion	1	3	2								1
Tyrannidae	*Mionectes*	1	496	151	1	1	1	1	1		1	
	Elaenia	3	75	32		2	1	2			1	1
	Zimmerius	1	23	15		1	1					
Turdidae	*Myadestes*	1	70	46	1	1	1	1				
	Turdus	3	72	19		1	1	1			1	
Thraupidae	*Anisognathus*	2	42	18							1	1
	Sporathraupis	1	28	17							1	
	Tangara	2	51	22		1					1	1

Table S10. Genera of insectivores and omnivores.

Number of bird species, captures, and events by family and genera for insectivores and omnivores across elevations. Models for 196 populations of 86 species in the Andes of northern Peru, 2015–2019. Complete spreadsheets with phenology metrics by population, input sampling effort (reproductive events/captures) and graphs of phenology models for each species across elevations available on Figshare (Newell et al. 2026).

					Species models by elevation (m)							
Family	*Genus*	Spp	Caps	Events	1700	1850	2010	2310	2520	2740	2780	3100
					Insectivores (suboscine)							
Caprimulgidae	*Nyctidromus*	1	5	3		1						
Picidae	*Picumnus*	1	5	3		1						
	Leuconotopicus	1	17	11					1	1	1	
	Veniliornis	1	5	4								1
Thamnophilidae	*Thamnophilus*	3	83	47	1			1		2	1	1
	Dysithamnus	1	124	74		1	1		1			
	Myrmotherula	1	57	31	1	1	1					
	Drymophila	1	20	12					1	1		
Conopophagidae	*Conopophaga*	1	54	30	1	1						
Grallariidae	*Grallaricula*	2	100	78		1		2	1		1	
Rhinocryptidae	*Scytalopus*	4	90	61	1	1		1	1	1	1	1
Furnariidae	*Xiphorhynchus*	1	14	7			1					
	Lepidocolaptes	1	56	23				1	1	1		1
	Pseudocolaptes	1	18	13							1	1
	Premnornis	1	198	103	1	1	1	1	1	1		
	Syndactyla	2	151	65		2	1	1	1	1		
	Thripadectes	1	7	5							1	
	Premnoplex	1	90	28	1		1	1	1			
	Margarornis	1	54	20					1	1		1
	Cranioleuca	2	38	16							1	1
	Synallaxis	2	124	85		1		1	1	1	1	1
Tyrannidae	*Leptopogon*	1	8	3			1					
	Pogonotriccus	1	16	9		1	1					
	Pseudotriccus	1	14	3						1		
	Hemitriccus	1	54	17							1	1
	Poecilotriccus	1	15	10			1	1				
	Tolmomyias	1	9	5				1				
	Pyrrhomyias	1	26	16						1	1	
	Mecocerculus	1	40	23							1	1
	Anairetes	1	20	11							1	1
	Phyllomyias	2	21	16		1					1	
	Myiophobus	1	38	19				1	1			
	Silvicultrix	3	109	41						1	2	2
	Ochthoeca	3	49	18							1	2
					Insectivores (oscine)							
Vireonidae	*Cyclarhis*	1	25	16							1	1
Troglodytidae	*Troglodytes*	2	82	40			1	1		1	1	1
	Cinnycerthia	1	41	16							1	
	Henicorhina	1	377	181	1	1	1	1	1	1		
	Cyphorhinus	1	60	27		1		1	1			
Turdidae	*Catharus*	1	86	27				1			1	
Passerellidae	*Chlorospingus*	1	9	7				1				

					Species models by elevation (m)							
Family	*Genus*	Spp	Caps	Events	1700	1850	2010	2310	2520	2740	2780	3100
					Insectivores (oscine)							
Parulidae	*Basileuterus*	1	77	39	1	1		1				
	Myiothlypis	3	1235	524	1	1	1	1	1	1	3	2
	Myioborus	2	253	130		1	1	2	1	1	1	1
Thraupidae	*Kleinothraupis*	1	24	8							1	
	Sphenopsis	1	29	16		1			1	1		
Thraupidae	*Thlypopsis*	1	31	11							1	1
	Conirostrum	1	6	4								1
	Diglossa	3	190	118				1		1	3	3
	Catamblyrhynchus	1	29	23							1	
	Pseudospingus	1	9	6								1
					Omnivores							
Columbidae	*Leptotila*	1	6	3		1						
	Zentrygon	1	10	7							1	
Strigidae	*Glaucidium*	1	14	7							1	1
Turdidae	*Turdus*	1	20	11								1
Passerellidae	*Arremon*	2	268	147	1	1	1	1	1	1	1	1
	Zonotrichia	1	22	11				1				1
	Atlapetes	1	124	63			1		1	1	1	1
Thraupidae	*Chlorornis*	1	6	5							1	

Model selection results

Table S11. Trait models for spatial variation in phenology

Model selection results for functional traits hypothesized to explain avian reproductive phenology [N_{F1} = 251 or N_{F2} = 127 for bimodal]. Phylogeny was included as a nested random effect at the family and genus level (1|family/genus/species) with repeated measures by landscape. Models with ΔAIC_c <2 are considered equivalent.

Model	K	AICc	ΔAICc	ModelLik	AICcWt	LL	Cum.Wt
			Peak timing				
Trophic guild	8	2939.70	0.00	1.00	1.00	-1461.55	1.00
Diet inverts (%)	7	2951.26	11.56	0.00	0.00	-1468.40	1.00
Nesting strategy	8	2963.91	24.21	0.00	0.00	-1473.66	1.00
Null model	6	2965.22	25.52	0.00	0.00	-1476.44	1.00
Roofed nest	7	2965.94	26.24	0.00	0.00	-1475.74	1.00
			Bimodal peaks				
Diet inverts (%)	5	129.29	0.00	1.00	0.60	-59.38	0.60
Trophic guild	7	130.85	1.56	0.46	0.28	-57.92	0.88
Null model	5	133.12	3.84	0.15	0.09	-61.30	0.97
Roofed nest	6	135.17	5.89	0.05	0.03	-61.21	1.00

Table S12. Spatial model selection for nectarivores

Model selection results by spatial location, rainfall, and functional traits [N_{F1} = 46 or N_{F2} = 27 for bimodal]. Phylogeny was included as a nested random effect at the family and genus level (1|family/genus/species) with repeated measures by landscape. Models with ΔAIC_c <2 are considered equivalent. Range indicates months for seasonal metrics.

Model	K	AICc	ΔAICc	ModelLik	AICcWt	LL	Cum.Wt
			Peak timing				
Latitude + Longitude	8	482.02	0.00	1.00	0.26	-231.06	0.26
Flowers 10-12	7	483.13	1.12	0.57	0.15	-233.09	0.41
Rain 7-9	7	483.98	1.96	0.38	0.10	-233.52	0.51
Cloud 6-9	7	484.21	2.20	0.33	0.09	-233.63	0.59
Body mass	7	484.39	2.38	0.30	0.08	-233.72	0.67
Null model	6	484.44	2.42	0.30	0.08	-235.14	0.75
Flower timing	7	485.39	3.37	0.19	0.05	-234.22	0.80
Flowers 1-3	7	485.57	3.55	0.17	0.04	-234.31	0.84
Rain 10-12	7	486.31	4.29	0.12	0.03	-234.68	0.87
Flowers 4-6	7	486.59	4.57	0.10	0.03	-234.82	0.90
Roofed nest	7	486.65	4.63	0.10	0.03	-234.85	0.93
Flowers 7-9	7	487.20	5.19	0.07	0.02	-235.13	0.95
Elevation	7	487.23	5.22	0.07	0.02	-235.14	0.96
Wet timing	8	487.85	5.83	0.05	0.01	-233.98	0.98
Rain 4-6	8	488.64	6.62	0.04	0.01	-234.37	0.99
Rain 1-3	8	489.12	7.10	0.03	0.01	-234.61	1.00
Dry timing	8	490.15	8.13	0.02	0.00	-235.13	1.00

Table S13. Spatial model selection for frugivores

Model selection results by spatial location, rainfall, and functional traits [N_{F1} = 40 or N_{F2} = 16 for bimodal]. Phylogeny was included as a nested random effect at the family and genus level (1|family/genus/species) with repeated measures by landscape. Models with ΔAIC_c <2 are considered equivalent. Range indicates months for seasonal metrics.

Model	K	AICc	ΔAICc	ModelLik	AICcWt	LL	Cum.Wt
			Peak timing				
Rain 7-9	7	442.36	0.00	1.00	0.90	-212.43	0.90
Elevation	7	447.50	5.14	0.08	0.07	-215.00	0.97
Null model	6	452.30	9.94	0.01	0.01	-218.88	0.97
Latitude + Longitude	8	452.54	10.19	0.01	0.01	-215.95	0.98
Rain 4-6	8	453.30	10.95	0.00	0.00	-216.33	0.98
Rain 10-12	7	454.11	11.76	0.00	0.00	-218.31	0.98
Fruit timing	7	454.15	11.79	0.00	0.00	-218.33	0.99
Roofed nest	7	454.70	12.34	0.00	0.00	-218.60	0.99
Fruit 7-9	7	454.79	12.43	0.00	0.00	-218.64	0.99
Fruit 10-12	7	455.00	12.64	0.00	0.00	-218.75	0.99
Cloud 6-9	7	455.03	12.67	0.00	0.00	-218.77	0.99
Body mass	7	455.14	12.78	0.00	0.00	-218.82	1.00
Fruit 4-6	7	455.15	12.79	0.00	0.00	-218.82	1.00
Fruit 1-3	7	455.22	12.86	0.00	0.00	-218.86	1.00
Rain 1-3	8	456.73	14.38	0.00	0.00	-218.04	1.00
Wet timing	8	458.23	15.87	0.00	0.00	-218.79	1.00
Dry timing	8	458.38	16.02	0.00	0.00	-218.87	1.00

Table S14. Spatial model selection for insectivores and omnivores

Model selection results by spatial location, rainfall, and functional traits [N_{F1} = 165 or N_{F2} = 77 for bimodal]. Phylogeny was included as a nested random effect at the family and genus level (1|family/genus/species) with repeated measures by landscape. Models with ΔAIC_c <2 are considered equivalent. Range indicates months for seasonal metrics.

Model	K	AICc	ΔAICc	ModelLik	AICcWt	LL	Cum.Wt
			Peak timing				
Arthropods dry threshold	9	1932.94	0.00	1.00	0.66	-956.89	0.66
Latitude + Longitude	8	1934.35	1.41	0.49	0.33	-958.71	0.99
Rain 4-6	7	1942.09	9.16	0.01	0.01	-963.69	0.99
Arthropods 7-9	7	1943.66	10.72	0.00	0.00	-964.47	1.00
Wet timing	7	1946.36	13.43	0.00	0.00	-965.83	1.00
Rain 7-9	7	1947.50	14.56	0.00	0.00	-966.39	1.00
Elevation	7	1948.39	15.45	0.00	0.00	-966.84	1.00
Null model	6	1948.42	15.48	0.00	0.00	-967.94	1.00
Arthropod timing	7	1949.17	16.23	0.00	0.00	-967.23	1.00
Roofed nest	7	1949.18	16.24	0.00	0.00	-967.23	1.00
Dry timing	7	1949.54	16.60	0.00	0.00	-967.41	1.00
Arthropods 10-12	7	1949.57	16.64	0.00	0.00	-967.43	1.00
Body mass	7	1950.13	17.20	0.00	0.00	-967.71	1.00
Rain 10-12	7	1950.35	17.41	0.00	0.00	-967.82	1.00
Rain 1-3	7	1950.53	17.59	0.00	0.00	-967.91	1.00
Arthropods 4-6	7	1950.57	17.63	0.00	0.00	-967.93	1.00
Arthropods 1-3	7	1950.59	17.65	0.00	0.00	-967.94	1.00
Cloud 6-9	7	1950.60	17.66	0.00	0.00	-967.94	1.00
			Bimodal peaks				
Arthropods 4-6	6	99.17	0.00	1.00	0.62	-42.99	0.62
Cloud 6-9	6	103.04	3.86	0.14	0.09	-44.92	0.71
Rain 10-12	6	103.22	4.05	0.13	0.08	-45.01	0.80
Rain 1-3	6	104.97	5.80	0.06	0.03	-45.89	0.83
Wet timing	6	105.56	6.38	0.04	0.03	-46.18	0.86
Null model	5	106.17	7.00	0.03	0.02	-47.66	0.88
Rain 7-9	5	106.28	7.11	0.03	0.02	-47.72	0.89
Arthropod timing	6	106.40	7.22	0.03	0.02	-46.60	0.91
Dry timing	6	106.64	7.46	0.02	0.01	-46.72	0.93
Arthropods 7-9	6	106.68	7.50	0.02	0.01	-46.74	0.94
Arthropods 1-3	6	106.71	7.53	0.02	0.01	-46.75	0.95
Rain 4-6	6	108.05	8.88	0.01	0.01	-47.43	0.96
Body mass	6	108.12	8.95	0.01	0.01	-47.46	0.97
Latitude + Longitude	7	108.20	9.02	0.01	0.01	-46.29	0.98
Elevation	6	108.30	9.13	0.01	0.01	-47.55	0.98
Roofed nest	6	108.51	9.34	0.01	0.01	-47.66	0.99
Arthropods 10-12	6	108.52	9.35	0.01	0.01	-47.66	0.99
Nesting timing	6	108.53	9.35	0.01	0.01	-47.66	1.00

Table S15. Spatial model selection for rainfall by month

Interaction between diet and 90-day rainfall accumulation [N_{F1} = 251 or N_{F2} = 127 for bimodal]. Models were run for all months of the year to select rainfall accumulation time periods. Top models were included in the final analysis. Phylogeny was included as a nested random effect at the family and genus level (1|family/genus/species) with repeated measures by landscape. Models with ΔAIC_c <2 are considered equivalent. Range indicates months for seasonal metrics.

Model	K	AICc	ΔAICc	ModelLik	AICcWt	LL	Cum.Wt
			Peak timing				
Diet * Rain 4-6	11	3008.20	0.00	1.00	0.97	-1492.55	0.97
Diet * Rain 5-7	11	3015.56	7.36	0.03	0.02	-1496.23	1.00
Diet * Rain 6-8	11	3022.77	14.57	0.00	0.00	-1499.83	1.00
Diet * Rain 7-9	11	3022.89	14.69	0.00	0.00	-1499.89	1.00
Diet * Rain 3-5	11	3023.44	15.24	0.00	0.00	-1500.17	1.00
Diet * Rain 2-4	11	3033.33	25.13	0.00	0.00	-1505.11	1.00
Diet * Rain 1-3	11	3039.32	31.12	0.00	0.00	-1508.11	1.00
Diet * Rain 11-1	11	3041.11	32.91	0.00	0.00	-1509.00	1.00
Diet * Rain 9-11	11	3041.20	33.00	0.00	0.00	-1509.05	1.00
Diet * Rain 8-10	11	3042.39	34.19	0.00	0.00	-1509.64	1.00
Diet * Rain 12-2	11	3042.47	34.27	0.00	0.00	-1509.68	1.00
Diet * Rain 10-12	12	3044.00	35.80	0.00	0.00	-1509.34	1.00
Null model	8	3044.85	36.65	0.00	0.00	-1514.13	1.00
			Bimodal peaks				
Diet + Rain 10-12	8	127.02	0.00	1.00	0.33	-54.86	0.33
Diet + Rain 12-2	8	127.88	0.86	0.65	0.22	-55.29	0.55
Diet + Rain 1-3	8	129.41	2.39	0.30	0.10	-56.06	0.65
Diet + Rain 9-11	8	129.77	2.76	0.25	0.08	-56.24	0.73
Diet + Rain 11-1	8	130.63	3.61	0.16	0.05	-56.66	0.78
Null model	7	130.85	3.83	0.15	0.05	-57.92	0.83
Diet + Rain 8-10	8	130.91	3.89	0.14	0.05	-56.81	0.88
Diet + Rain 2-4	8	131.58	4.56	0.10	0.03	-57.14	0.91
Diet + Rain 5-7	8	132.85	5.83	0.05	0.02	-57.78	0.93
Diet + Rain 6-8	8	132.85	5.84	0.05	0.02	-57.78	0.95
Diet + Rain 3-5	8	132.89	5.87	0.05	0.02	-57.79	0.97
Diet + Rain 7-9	8	132.91	5.90	0.05	0.02	-57.81	0.98
Diet + Rain 4-6	8	133.09	6.07	0.05	0.02	-57.90	1.00

Table S16. Temporal model selection by trophic guild.

Model selection results by diet for lagged effects of rainfall and resources on reproductive timing and effort (peak magnitude). Phylogeny was included as a nested random effect at the family and genus level (1|family/genus/species) with repeated measures by landscape. Models with ΔAIC_c <2 are considered equivalent.

Model	K	AICc	ΔAICc	ModelLik	AICcWt	LL	Cum.Wt
Nectarivores (*N* = 62)							
Peak timing							
Null model	7	209.12	0.00	1.00	0.25	-96.52	0.25
Rain 180-days	8	210.25	1.13	0.57	0.14	-95.76	0.40
Rain 360-days	8	210.25	1.13	0.57	0.14	-95.77	0.54
Rain 90-days	8	210.63	1.52	0.47	0.12	-95.96	0.66
Rain 150-days	8	211.21	2.09	0.35	0.09	-96.25	0.75
Rain 120-days	8	211.26	2.14	0.34	0.09	-96.27	0.84
Rain 30-days	8	211.28	2.16	0.34	0.09	-96.28	0.93
Rain 60-days	8	211.63	2.51	0.29	0.07	-96.45	1.00
Peak magnitude							
Null model	7	524.44	0.00	1.00	0.25	-254.18	0.25
Rain 90-days	8	525.37	0.93	0.63	0.16	-253.33	0.41
Rain 360-days	8	525.78	1.34	0.51	0.13	-253.53	0.54
Rain 180-days	8	525.79	1.35	0.51	0.13	-253.54	0.66
Rain 150-days	8	526.19	1.75	0.42	0.10	-253.73	0.77
Rain 60-days	8	526.32	1.88	0.39	0.10	-253.80	0.87
Rain 120-days	8	527.07	2.63	0.27	0.07	-254.18	0.93
Rain 30-days	8	527.08	2.64	0.27	0.07	-254.18	1.00
Frugivores (*N* = 22)							
Peak timing							
Null model	7	76.95	0.00	1.00	0.63	-27.47	0.63
Rain 120-days	8	81.61	4.66	0.10	0.06	-27.27	0.69
Rain 90-days	8	81.68	4.73	0.09	0.06	-27.30	0.75
Rain 60-days	8	81.92	4.97	0.08	0.05	-27.42	0.80
Rain 360-days	8	81.98	5.03	0.08	0.05	-27.45	0.85
Rain 150-days	8	82.01	5.06	0.08	0.05	-27.47	0.90
Rain 30-days	8	82.02	5.07	0.08	0.05	-27.47	0.95
Rain 180-days	8	82.02	5.07	0.08	0.05	-27.47	1.00
Peak magnitude							
Rain 90-days	8	197.96	0.00	1.00	0.38	-85.44	0.38
Rain 60-days	8	198.91	0.95	0.62	0.23	-85.92	0.61
Rain 120-days	8	199.56	1.61	0.45	0.17	-86.24	0.78
Rain 180-days	8	201.21	3.26	0.20	0.07	-87.07	0.85
Rain 150-days	8	201.41	3.45	0.18	0.07	-87.16	0.92
Null model	7	201.83	3.87	0.14	0.05	-89.91	0.98
Rain 360-days	8	204.81	6.86	0.03	0.01	-88.87	0.99
Rain 30-days	8	204.86	6.91	0.03	0.01	-88.89	1.00
Insectivores[1] (*N* = 97)							
Peak timing							
Arthropods 30-days	8	370.13	0.00	1.00	0.54	-176.25	0.54
Rain 180-days	10	372.15	2.01	0.37	0.20	-174.79	0.74
Rain 120-days	10	373.66	3.53	0.17	0.09	-175.55	0.83
Arthropods 90-days	8	373.68	3.54	0.17	0.09	-178.02	0.92
Rain 150-days	10	375.29	5.16	0.08	0.04	-176.37	0.96
Arthropods 180-days	8	376.21	6.08	0.05	0.03	-179.29	0.99
Null model	8	378.96	8.83	0.01	0.01	-180.66	1.00

Model	K	AICc	ΔAICc	ModelLik	AICcWt	LL	Cum.Wt
Rain 90-days	10	380.84	10.70	0.00	0.00	-179.14	1.00
Rain 60-days	10	383.44	13.31	0.00	0.00	-180.44	1.00
Rain 30-days	10	383.48	13.35	0.00	0.00	-180.46	1.00
			Peak magnitude				
Arthropods 90-days	8	804.65	0.00	1.00	0.82	-393.51	0.82
Rain 150-days	10	809.93	5.28	0.07	0.06	-393.69	0.88
Null model	8	810.62	5.97	0.05	0.04	-396.49	0.92
Rain 180-days	8	811.89	7.24	0.03	0.02	-397.13	0.94
Rain 120-days	8	812.12	7.47	0.02	0.02	-397.24	0.96
Arthropods 30-days	10	812.37	7.72	0.02	0.02	-394.91	0.98
Rain 30-days	10	813.37	8.72	0.01	0.01	-395.41	0.99
Arthropods 180-days	10	814.27	9.61	0.01	0.01	-395.85	1.00
Rain 60-days	10	816.38	11.73	0.00	0.00	-396.91	1.00
Rain 90-days	10	816.43	11.77	0.00	0.00	-396.93	1.00

[1]Omnivores were not included with insectivores because models fit the data poorly.

Table S17. Model selection for confirmatory path analysis

Support for fitted model with links selected based on *d*-separation tests of model fit (Shipley 2013) compared to a saturated model (all links), as well as the most parsimonious link structure (fewest links). Confirmatory path analysis models fit the data based on Fisher's C tests ($P > 0.05$) using piecewiseSEM (Lefcheck 2016) including repeated measures with crossed random intercepts for phylogeny (1|family/genus/species) + (1|landscape) with landscapes weighted equally in the analysis. Models with ΔAIC_c <2 are considered equivalent.

Model	K	AIC d-sep	ΔAIC d-sep	AICcWt	Fisher's C	*df*	*P*-val
Nectarivores shift (N_{F0} = 50)							
Indirect	27	68.48	0.00	0.82	14.48	12	0.27
Saturated	32	71.53	3.05	0.18	7.53	2	0.02
Parsimonious	21	99.15	30.67	0.00	57.15	24	0.00
Frugivores shift (N_{F0} = 55)							
Indirect	30	66.48	0.00	0.90	6.48	6	0.37
Saturated	32	70.91	4.43	0.10	6.91	2	0.03
Parsimonious	21	121.76	55.28	0.00	79.76	24	0.00
Insectivores/omnivores shift (N_{F0} = 196)							
Indirect	20	50.45	0.00	1.00	10.45	8	0.24
Saturated	23	61.55	173.81	0.00	15.55	2	0.00
Parsimonious	17	85.52	208.57	0.00	51.52	14	0.00
Insectivores/omnivores bimodal (N_{F2} = 77)							
Indirect	25	62.82	0.00	0.97	12.82	16	0.69
Saturated	31	69.88	7.06	0.03	7.88	2	0.02
Parsimonious	20	142.35	79.53	0.00	102.35	24	0.00

SI Extended Acknowledgements/Collaborators in Perú

The idea for this project came out of work by FLN in Honduras thanks to S. Glowinski, along with foundational training in bird ageing and molt with A. Leppold, B. Mulvihill, and R. Leberman at Powdermill Avian Research Center. We are grateful to staff at the Florida Museum and University of Bern for support of work-life balance to complete this work. We would like to thank the many collaborators in Perú who contributed support and assistance throughout including the following organizations and individuals:

LANDHOLDERS

La Red de Áreas de Conservación Privada de Amazonas (Red AMA).

José de la Torre, presidente**,** Perico Heredia, vicepresidente**,** Leyda Gueiler Rimarachín Cayatopa, secretaria.

Área de Conservación Privada de Bosque Berlín, Bagua Grande.

Ricardo Rimarachín, Carmela Cayatopa, Leyda Gueiler Rimarachín Cayatopa, Victoria Rimarachín Cayatopa

Comunidad Campesina de Beirut, Corosha, Bongára.

Edwin Guiva, presidente, Blanca Guivin, Carmen Guiva, Demasio Edkin y Teodora, Homero "Pancho" Lopez, Lucas Vegas, Marcos Guadelupe, Maria Llaja

Área de Conservación Privada de Huiquilla, Choctámal.

José de la Torre, Miguel Cruz

Comunidad Campesina de Levanto.

Presidentes de la comunidad: Asuncion Torres, Emerehildo Salombavilde, Don Santos, y Alejandro Cruz

Comunidad Campesina de Mayno.

Don Teodoro, presidente de la comunidad

Fundo Ecológico Don Diego, Pomacochas, y Nuevo Guallulo.

Dario Cotrina, Diego Poclín Tuesta, Ernesto Arajo, Francisca Tuestadet, Joel Poclín, Doña Juana, Paulino Arajo

Área de Conservación Privada Bosque de Palmeras de Ocol, Molinopampa.

Elmira Muñoz Jil, Irma Cruz Viaya, Marleny Servan Cruz, Nixon Pilco Melendez, Orfita Mori Cachay, Pablo Mori Cachay, Patricia Rimachi Diaz, Rosa Amelia Diaz Perea

Comunidad Campesina de San Lorenzo.

Aparicio Medina Vasquez, presidente de la comunidad, Efijenio Katpo, Juan José Chicano, Kelmar Valle, Secundino Rojas, Vincente Chavez y esposa.

Comunidad Campesina de Velapata.

Mario Tuesta Vargas y familia

FOOD, LODGING, & GUIDES

Comunidad Campesina de Beirut, Corosha, Bongára.

Asociación Turística de Beirut: Adelayda Chosguild Tioloc, Alberto Saul Benavides Gonzales, Bernardina Mirano Mas, Graciola Campos de la Cruz, Jorge Juarez, Judith Oyarce Ehasquibol, Lizeth Arista Aybar, Lucas Vega Jr., Maria Herlit Goñas Sopla, Miriarb Portacarrero Lápiz, Neli Montenegro Llaja, Rosario Gómez Montenegro, Sarai Cieza Goñaas, Silvia Valle Rojas, Victor Ramos, Yuly Rojas Diaz, Zoilita del Pilar Torres Jauregui, Zoyla Marina Goñas de Cieza

Área de Conservación Privada de Bosque Berlín, Bagua Grande.

Leyda Gueiler Rimarachín Cayatopa, Victoria Rimarachín Cayatopa

Área de Conservación Privada de Huiquilla, Choctámal.

Alejandro Cuipal Chuquisuta, Alsira Chavez Iop, Alejo and Jonny Cuipal Chavez

Comunidad Campesina de Levanto.

Jorge Mendoza

Fundo Ecológico Don Diego, Pomacochas and Nuevo Guallulo.

Diego Poclín Pinedo, Elda Diaz Gamuro

Área de Conservación Privada Bosque Palmeras de Ocól, Molinopampa.

Asociación de Las Mujeres de ACP Palmeras de Ocol: Dani Juanita Diaz Rojas, Diana Carina Mori Servan, Ena Carmela Tamallo Diaz, Graciela Tafur Maldonado, Marlene Servan Cruz, Marta Rojas Gomez, Olger Callilgos Acosta, Pablo Mori Cachay, Pablito Mori Servan, Patricia Rimachi Diaz, Rosa Amelia Diaz Perea, Teunilo Calampa Santijan, Wilmer Vicaya Sopla

Comunidad Campesina de San Lorenzo.

Felicita Vasquez Altamirano, Idelfia Ruiz Villanueva, Rodolfo Banda, Wilson Loaisa

Comunidad Campesina de Velapata.

Genarita Cruz y pareja

Bosque de Protección Alto Mayo, Venceremos

Ivonne Paico Vera, Jhonny Ramos, Jolo Elias Cercado Cabrera, Jorge Luis Altantara Veía, and many other guardaparques.

TRANSPORTATION
Humberto Vilca, Dago (Bagua Grande), Anival (Levanto), Orlando (Levanto), Noé (Levanto), Cleo (Chóctamal), Jhonny Cuipal (Choctámal), Justino (Choctámal),Orlando Cruz (Choctámal).

PERMITS
Lizeth Natali Cayo Rodriguez, Marco A. Enciso (SERFOR), Priscilla Pellisier (CORBIDI), Thomas Valqui (CORBIDI), Silvia Ivonne Paico Vera (BPAM).

WEATHER DATA
Ivonne Paico Vera & park guards (BPAM), Wagner Guzman Castillo (IIAP), Períco Heredia Arce (Red AMA), Rolando Salas López & Elgar Barboza Castillo (Universidad Toribio Rodriguez de Mendoza INDES-CES). Communities & Red AMA: Homero Francisco Lopez, Maria Llaja; Diego Poclín Pinedo, Diego Poclín Tuesta, Elda Díaz Guamuro, Vincente Chavez & family, Leyda Gueiler & Victoria Rimarachín Cayatopa, Ricardo Rimarachín, Camila Cayatopa, Pablo Mori Cachay, Marlene Servan Cruz, Diana Carina & Pablito Mori Servan, Humberto & Segundo Vilca, Jose La Torre, Alsirra Cuipal Chuquisuta, Alejo Chavez.

OTHER
Fernando Angulo Pratolongo, Antonio García Bravo (CORBIDI), Carlos Altamirano (Wankanki), Wagner Guzmán Castillo, Lizette Mendez Fasabi, Perico Heredia Arce, Emperatriz Muñoz, Imanol Martin Gonzalez, Marina Cruz Santíllán Trigoso, José Llaja Soplin & Dona Regalado Fernandez (Chachapoyas Backpackers), Karen Marie Pedersen, Maddy Stokes (Peace Corps)

PHOTO CREDITS
Pollination, water loss, and fruit development
Photos by Felicity Newell of *Bomarea*, Geometrid larvae, *Hedyosmum*, Amazonas Perú

Entomopathogens
Photo by Andreas Kay, Ecuador
Source: https://www.flickr.com/photos/andreaskay/44264582075

Frugivores, nectarivores, insectivores
Photos by Ian Ausprey of Thraupidae, Trochilidae, Furnariidae, Amazonas, Perú

Diglossa
Photos by Felicity Newell of *Diglossa cyanea*, Amazonas, Perú
